# Evidence for Pathogen‐Driven Selection Acting on *HLA‐DPB1* in Response to *Plasmodium falciparum* Malaria in West Africa

**DOI:** 10.1002/ece3.70933

**Published:** 2025-02-24

**Authors:** Thomas Goeury, Ndeye Faye, Pascale Gerbault, Viktor Černý, Eric Crubézy, Jacques Chiaroni, Hacene Brouk, Lydie Brunet, Maxime Galan, Natasja G. de Groot, José Manuel Nunes, Alicia Sanchez‐Mazas

**Affiliations:** ^1^ Laboratory of Anthropology, Genetics and Peopling History (AGP), Department of Genetics and Evolution University of Geneva Geneva Switzerland; ^2^ Institute of Archaeology of the Academy of Sciences of the Czech Republic Archaeogenetics Laboratory Czech Academy of Sciences Prague Czech Republic; ^3^ Institut Universitaire de France UMR5288 CNRS University of Toulouse III Paul Sabatier Toulouse France; ^4^ ADES UMR 7268 Aix Marseille University, EFS, CNRS Marseille France; ^5^ Service of Hemobiology and Blood Transfusion University Hospital Center Ibn Rochd of Annaba Faculty of Medicine Badji Mokhtar University of Annaba Annaba Algeria; ^6^ Transplantation Immunology Unit and National Reference Laboratory for Histocompatibility (UIT/LNRH) Geneva University Hospital Geneva Switzerland; ^7^ CBGP UMR 1062 INRAE IRD CIRAD Montpellier SupAgro University of Montpellier Montpellier France; ^8^ Department of Comparative Genetics and Refinement Biomedical Primate Research Centre (BPRC) Rijswijk the Netherlands; ^9^ Institute of Genetics and Genomics in Geneva (IGE3) University of Geneva Geneva Switzerland

**Keywords:** Africa, HLA, human molecular diversity, malaria, pathogen‐driven selection, *plasmodium falciparum*

## Abstract

African populations remain underrepresented in studies of human genetic diversity, despite a growing interest in understanding how they have adapted to the diverse environments they live in. In particular, understanding the genetic basis of immune adaptation to pathogens is of paramount importance in a continent such as Africa, where the burden of infectious diseases is a major public health challenge. In this study, we investigated the molecular variation of four Human Leukocyte Antigens (*HLA*) class II genes (*DRB1*, *DQA1*, *DQB1* and *DPB1*), directly involved in the immune response to parasitic infections, in more than 1000 individuals from 23 populations across North, East, Central and West Africa. By analyzing the *HLA* molecular diversity of these populations in relation to various geographical, cultural and environmental factors, we identified divergent genetic profiles for several (semi‐)nomadic populations of the Sahel belt as a signature of their unique demography. In addition, we observed significant genetic structuring supporting both substantial geographic and linguistic differentiations within West Africa. Furthermore, neutrality tests suggest balancing selection has been shaping the diversity of these four *HLA* class II genes, which is consistent with molecular comparisons between *HLA* genes and their orthologs in chimpanzees (*Patr*). However, the most striking observation comes from linear modeling, demonstrating that the prevalence of *Plasmodium falciparum*, the primary pathogen of malaria in Africa, significantly explains a large proportion of the nucleotide diversity observed at the *DPB1* gene. DPB1*01:01, a highly frequent allele in Burkinabé populations, is identified as a potential protective allele against malaria, suggesting that strong pathogen‐driven positive selection at this gene has shaped *HLA* variation in Africa. Additionally, two low‐frequency *DRB1* alleles, DRB1*08:06 and DRB1*11:02, also show significant associations with *P. falciparum* prevalence, supporting resistance to malaria is determined by multigenic and/or multiallelic combinations rather than single allele effects.

## Graphical abstract

This study analyzed HLA class II genes in over 1,000 individuals from 23 African populations, revealing genetic diversity influenced by geography, culture, and environment, with a significant link between *Plasmodium falciparum* prevalence and variation at the *DPB1* gene. The research identifies specific alleles, such as DPB1*01:01, as potentially protective against malaria, suggesting pathogen‐driven selection shapes immune gene diversity in Africa. It also suggests that resistance to malaria is determined by multigenic and/or multiallelic combinations rather than single allele effects.

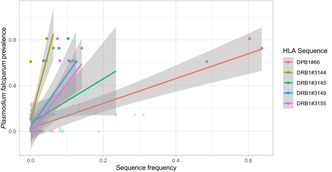

## 1 Introduction

Throughout human history, infectious diseases have spread across large geographical areas, with significant impact on human populations (Baker et al. 2017; Houldcroft and Underdown 2023), as exemplified by the recent COVID‐19 pandemic (Ashmore and Sherwood 2023). One such disease is malaria, with elevated mortality rates in geographical regions like inter‐tropical sub‐Saharan Africa (World Health Organization 2022). In these areas, prevention and control of malaria request the skills of a wide range of researchers involved in health‐related scientific fields, including immunogenetics. Paradoxically, sub‐Saharan African populations remain underrepresented in studies reporting human genetic diversity worldwide (Fatumo and Choudhury 2023; The 1000 Genomes Project Consortium 2015), more particularly for Human Leukocyte Antigen (*HLA*) genes—the Major Histocompatibility Complex (*MHC*) genes in humans—which are among the most polymorphic of our genome (Jin et al. 2018; Vandiedonck and Knight 2009) and play a crucial role in adaptive immunity.

In this context, our current research program, *HLA‐AFRICA*, is dedicated to the characterization and evolution of *HLA* molecular diversity in African populations. Besides our deep interest in reconstructing the complex history of these populations through the prism of genetics, our objective is to identify signatures of various evolutionary mechanisms that have shaped *HLA* genetic patterns across this continent, with a specific focus on adaptative immune responses to endemic pathogens such as *Plasmodium falciparum* (*P. falciparum*), the causative agent of the most severe form of malaria. In a previous study, based on two *HLA* class I genes (*HLA‐A* and *HLA‐B*), we showed that natural selection driven by *P. falciparum* significantly influenced *HLA‐B* genetic variation across African populations (Sanchez‐Mazas et al. 2017). Our results confirmed the association of HLA‐B*53:01, which has previously been suggested to have a protective effect in case–control studies (Hill et al. 1991), and also revealed a potential protective role conferred by another *HLA‐B* allele, B*78:01, belonging to the same functional *B07* supertype as B*53:01 (Sidney et al. 2008). Interestingly, among our closest relatives, bonobos are nearly free of the malaria parasite except at one of the 11 studied locations where bonobos occur in the wild (Liu et al. 2017). At this location, bonobos' *B07*‐like allotypes display a significantly higher frequency and are predominant among infected individuals (de Groot, Stevens, and Bontrop 2018; Wroblewski et al. 2023). A specific epitope (ls6) of the malaria pathogen (LSA‐1 protein), which is exclusively expressed at the liver‐stage of infection, serves as a potential target of CD8+ T cells restricted by *B07* allotypes. The presence of *B07* allotypes may thereby reduce the parasite burden spreading to erythrocytes and help prevent the development of severe malaria (Wroblewski et al. 2023).

While *HLA* class I molecules protect against the development of malaria through cytotoxic CD8+ T lymphocytotoxicity at the liver stage of *P. falciparum* infection (Aidoo and Udhayakumar 2000) and/or through interaction with Natural Killer cells (Tukwasibwe et al. 2020), *HLA* class II molecules trigger the production of specific antibodies (humoral response) against *P. falciparum* proteins at all stages of the parasitic infection (Fiorillo et al. 2017; Medhasi and Chantratita 2022; Meyer et al. 2006). To unravel additional signatures of malaria‐driven selection, we genotyped four *HLA* class II genes, *DRB1*, *DQA1*, *DQB1* and *DPB1*, in (almost) the same set of African populations previously analyzed for *HLA‐A* and *HLA‐B* (Sanchez‐Mazas et al. 2017). Here, we report the results of an extensive analysis of these polymorphic genes based on their exon 2 sequences. These exons encode the peptide‐binding region (PBR) of the *HLA* molecules (on the α chain for *DQA1* and on the β chain for *DRB1*, *DQB1* and *DPB1*), which is essential for the presentation of pathogenic peptides to CD4+ T cells, a crucial step of the humoral immune response. We performed various population genetic analyses on 23 population samples (comprising 1039–1248 individuals, depending on the gene) taking into account several geographical, cultural and environmental characteristics of these populations, including exposure to *P. falciparum*. This led to suggest specific hypotheses about the evolutionary mechanisms that have shaped the molecular diversity observed at each of these four *HLA* class II genes across the continent.

Our results show that, while the unique demographic histories of the populations studied have left significant signatures at all four *HLA* class II genes, this part of the *HLA* genomic region is also affected by both balancing and pathogen‐driven selection in response to malaria.

## 2 Materials and Methods

### 2.1 Population Samples and Ethics Approval

**FIGURE 1:**
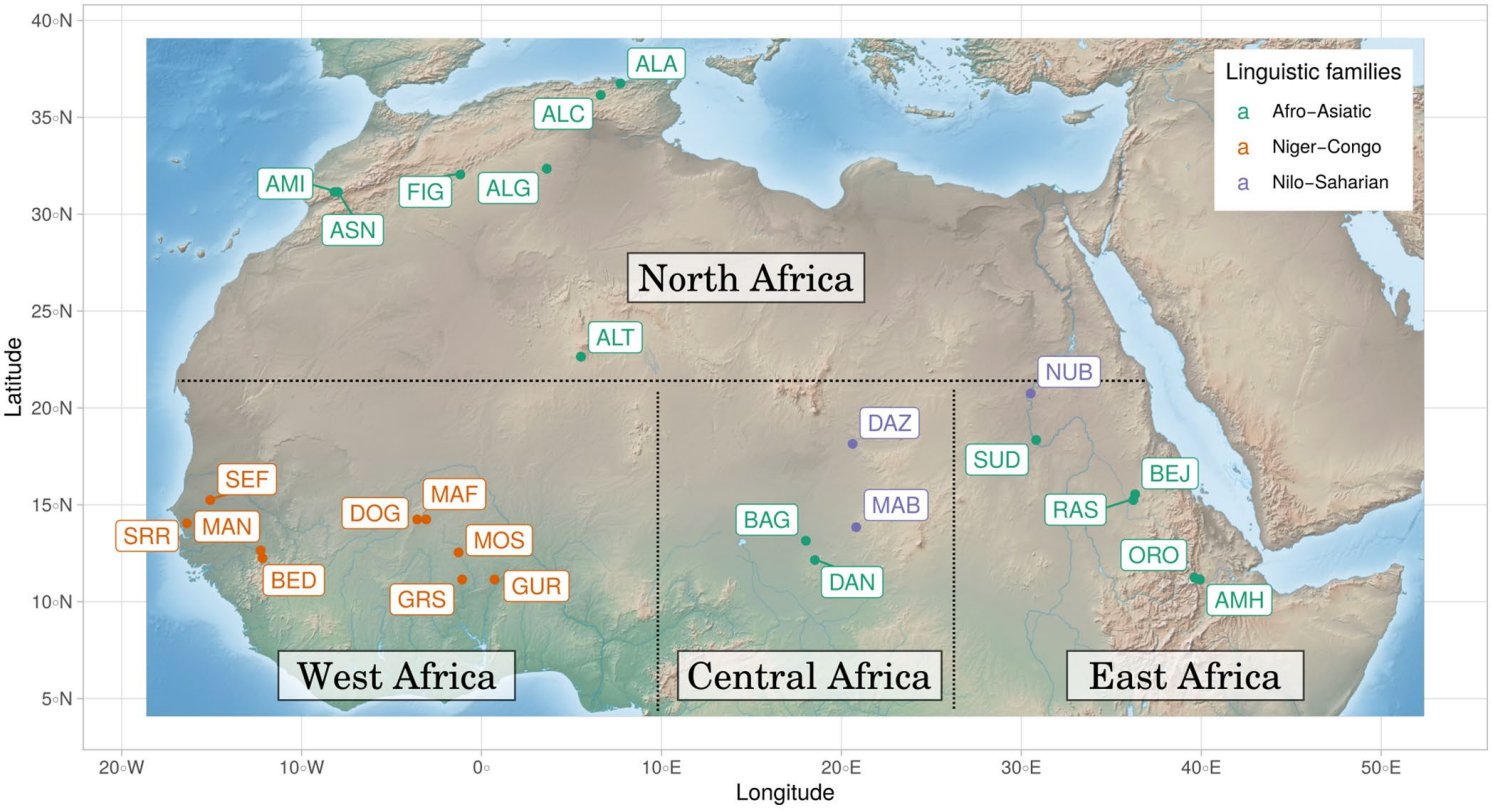
Map of the populations sampled. Map of the populations sampled and investigated in this study, with their sampling location and linguistic families. Dashed lines indicate the different geographical regions considered here. BED: Senegal‐Bedik; MAN: Senegal‐Mandenka; SRR: Senegal‐Serer; SEF: Senegal‐Fulani; DOG: Mali‐Dogon; MAF: Mali‐Fulani; GUR: BurkinaFaso‐Gurmantche; GRS: BurkinaFaso‐Gurunsi; MOS: BurkinaFaso‐Mossi; BAG: Chad‐BaggaraArabs; DAN: Chad‐Dangaleat; DAZ: Chad‐Daza; MAB: Chad‐Maba; AMH: Ethiopia‐Amhara‐(Keketeya); ORO: Ethiopia‐Oromo; BEJ: Sudan‐BejaHadendoa; NUB: Sudan‐Nubians; RAS: Sudan‐RashaaydaArabs; SUD: Sudan‐SudaneseArabs; ALA: Algeria‐(Annaba); ALC: Algeria‐(Constantine); ALG: Algeria‐(Ghardaia); ALT: Algeria‐(Tamanrasset); AMI: Morocco‐Amazigh‐(Amizmiz); ASN: Morocco‐Amazigh‐(Asni); FIG: Morocco‐Amazigh‐(Figuig).

Details of the population samples are given in Table S1, including the principal investigator who collected the samples, the population name, short name, geographical information (region and coordinates), linguistic information (spoken language and associated linguistic family), lifestyle (sedentary, nomadic or semi‐nomadic) and sample sizes at the four *HLA* loci tested (*DRB1*, *DQA1*, *DQB1* and *DPB1*). All sampled individuals provided written or verbal informed consent to contribute biological material for the purpose of the study. The collected data were anonymized, and their use was approved by the Ethics Committee of the University of Geneva in Switzerland and the Swiss National Science Foundation. Only population samples with at least 20 individuals were considered for downstream analyses. Three populations (BED, MAF and ASN) did not meet this criterion for *DQB1*, therefore further analyses were not performed at this locus for these populations. World regions were defined according to the United Nations (UN) geo‐scheme and named according to Nunes et al. (2014). However, the Sudanese populations studied were grouped within East Africa rather than North Africa as they are much closer geographically to the East African populations studied. Figure 1 shows a map of the location, associated geographical region and linguistic family of all populations studied.

### 2.2 DNA Sequencing

The Mandenka (MAN) population was sampled by our team, as described in previous papers (Tiercy et al. 1992; Currat et al. 2002; Poloni et al. 1995; Graven et al. 1995; Dard et al. 1996; Sanchez‐Mazas et al. 2000; Martinson et al. 1995; Sabbagh et al. 2011; Goeury, Creary, Brunet, et al. 2018, Goeury, Creary, Fernandez‐Vina, et al. 2018). DNA of all other population samples was extracted in the appropriate collaborating laboratory (Table S1) and was then sent to Geneva for library preparation and next generation sequencing (NGS). Tagging and multiplexing methods designed by Galan et al. (Galan et al. 2010) for Roche 454 sequencing were used. This high‐throughput sequencing requires adaptors for the emPCR and 454 GS‐FLX pyrosequencing using Lib‐L Titanium Series reagents. This method was then used to amplify and sequence exons 2 of the four *HLA* class II genes *DRB1*, *DQA1*, *DQB1* and *DPB1*. For each gene, 3′446 to 3′458 samples (+12 control H_2_O) were amplified. A total of 306 to 504 replicates (8.8% to 14.6%) were used for protocol confirmation. Sequencing was performed by Beckman Coulter Genomics (Genomic Services, Danvers, MA, USA).

### 2.3 Reads Processing

Reads were filtered using Mothur (Schloss et al. 2009) with a minimal PhredScore of 30. Sequencing results were processed using *MADaM* software, described in our companion paper (Goeury, Nunes, and Sanchez‐Mazas n.d.), using the following parameters: T_1_ threshold of 50 reads, BLAST (Altschul et al. 1990) performed using a reference sequence for each locus (provided by M. Galan) and an eValue of 1e‐10, sequence lengths between 200 and 400 bp. For each locus, t‐distributed stochastic neighbor embedding (t‐SNE) (van der Maaten and Hinton 2008) was performed using perplexity values of 30, 40 and 50, with 5 different starting points each time (to mitigate the risk of a local minimum). Clustering was achieved with DBSCAN (Ester, Kriegel, and Xu 1996) and ε parameter between 0.3 and 3.5 (*DRB1*), 5.5 (*DQA1*), 3.8 (*DQB1*) and 4.3 (*DPB1*), with a step of 0.1.

Sequencing results for *DQB1* contained reads from *DQB2* exon 2 also amplified by the PCR primers because of the high similarity between these genes (Lenormand et al. 2012). Markovian filter (in *MADaM*) was thus trained on 931 *DQB1* and 5 *DQB2* sequences (the only ones available at the time) to filter out the *DQB2* reads. Similarly, PCR for *DRB1* also amplified exon 2 sequences of the paralogous *HLA* genes *DRB3*, *DRB4*, *DRB5*, *DRB6* and *DRB7* (Satta, Mayer, and Klein 1996). These reads were filtered out using a Markovian filter trained on 2′038 *DRB1* and *DRB3* sequences, 50 *DRB4*, 43 *DRB5*, 3 *DRB6* and 2 *DRB7* sequences (downloaded on 15/02/2020 from the IPD‐IMGT/HLA database v3.35 (Robinson et al. 2015)). *DRB3* sequences were removed by hand.

Markovian filter rejected respectively 183′221 and 130′149 reads (11.4% and 7.8% of total reads) for *DRB1* and *DQB1* (reads identified as sequences of non‐targeted loci: *DRB4*, *DRB*5, *DRB*6, *DRB*7 and *DQB2*). Table S2 shows the total number of reads, rejected reads, assigned reads, variants, genotyped samples, alleles found as well as the accuracy of the method (computed using the replicates) and the potential sources of error per locus.

The method shows robust results, with accuracy up to 98.5% (*DPB1*). The lowest scores, of 83.9% (*DQB1*) and 91.1% (*DQA1*) are due, for *DQB1*, to a residual presence of *DQB2* variants, which were not filtered out by the Markovian filter (due to the very low number of *DQB2* sequences used to train the filter) and, for *DQA1*, to an under‐amplifying variant (up to a factor 10 compared to the other variants). These loci were then thoroughly eye‐checked to resolve these issues.

To avoid considering (mistakenly) artifacts as true variants, only alleles present in at least two individuals (replicates excluded) were kept. Singleton alleles, which represented less than 1% at each locus (0.2% at *DRB1*& *DRB3*; 0.9% at *DQA1*; 0.2% at *DQB1* and 0.3% at *DPB1*) were considered as false positives (artefacts wrongly detected as true variants).

### 2.4 Sequence Names

*HLA* alleles are named in the IPD‐IMGT/HLA database by considering the variability both within and outside exon 2 (Robinson et al. 2015). These allele names are composed of the prefix *HLA*‐, the locus name in capital letters followed by a star and ending by the allele name, for example, HLA‐DRB1*13:04. To avoid confusion, we have adopted a specific nomenclature to distinguish between the IPD‐IMGT/HLA named alleles (hereafter named “*HLA* nominal alleles*”*) and the *HLA* exon 2 sequences reported here (hereafter named “*HLA* alleles” or “*HLA* sequences”) that we referred to with the locus name followed with a number sign “#” and the sequence number assigned by *MADaM*, for example, *DRB1#3135*. Table S3 reports the list of possible *HLA* nominal alleles associated with each *HLA* sequence identified in this study, indicating the 1st, 2nd and/or 3rd field levels of resolution of the *HLA* nominal alleles depending on the sequence variants identified.

### 2.5 Sequence Sub‐Regions

*HLA* exon 2 sequences, which encode the peptide‐binding region (*PBR*) of the *HLA* molecules, include a set of codons that make up the antigen recognition site (ARS), a putative target of balancing selection (Hughes and Nei 1988, 1989). As NGS generated precise DNA sequences, three different sets of nucleotides were distinguished for several analyses: “Whole Exon 2”, “ARS” and “non‐ARS”, corresponding, respectively, to the entire exon 2 sequence (to account for the diversity of the sequence as a whole), to the exon 2 codons coding for the ARS according to the positions defined by Reche and Reinherz (2003), and to the exon 2 codons not coding for the ARS. Table S4 shows the sequence lengths of the three sets of nucleotides per locus.

### 2.6 Population Genetics Analyses

#### 2.6.1 HLA Frequencies, Hardy–Weinberg and Linkage Disequilibrium

The frequencies of *HLA* exon 2 sequences, hereafter named allele frequencies, were estimated for each *HLA* locus using an Expectation–Maximization (EM) algorithm (Table S5, Figure S1). Due to the high level of polymorphism at *HLA* genes, a minimal threshold of 20 individuals was considered for the analyses, leading us to exclude the *DQB1* data for 3 populations (BED, MAF and ASN). Hardy–Weinberg Equilibrium (HWE) was then tested at each locus by comparing the likelihood of the observed allele frequency distribution under two models, i.e. HWE and inbreeding. The number of times HWE was rejected ranges from none (no population samples rejected HWE for *DPB1*) to nine (nine populations rejected HWE for *DRB1*). Population samples for which at least two loci rejected HWE (i.e., samples ALA, ALG and DOG) were excluded from the subsequent comparative analyses. In this case, HWE deviation may indeed be due to demographic factors (e.g., small population size, inbreeding, gene flow), which would also affect the frequency estimate at the other loci (the EM algorithm assumes HWE). On the other hand, HWE deviation at a single locus is more likely to indicate natural selection at that locus without affecting the frequency estimates at the other loci, which can then be used as controls. Most analyses were therefore performed on 23 populations (excluding 3 populations due to HWE deviation) and, in the case of *DQB1*, on 20 populations (excluding 3 additional populations with a too small sample size at that locus). The EM algorithm was also used to estimate the frequencies of each pair of exon 2 sequences at two different *HLA* loci (hereafter named two‐locus haplotype frequencies) and their linkage disequilibrium (*LD*) was tested by means of standardized residuals (Table S6). Global linkage disequilibrium (*GLD*), e.g. the association between each pair of loci, was also tested by a non‐parametric approach (Figure S2) (Nunes 2016). All analyses were performed with gene[rate] computer tools available on *hla‐net.eu* (Nunes et al. 2014).

#### 2.6.2 Genetic Distances and Population Structure

F_ST_‐derived Reynolds coefficient *Θ*_*w*_ (Reynolds, Weir, and Cockerham 1983) between populations were computed at each locus with arlequin v3.5 (Excoffier and Lischer 2010) and used as genetic distances to plot non‐metric multidimensional scaling (nMDS) analyses, using the R software library “vegan” (Oksanen 2019). In these plots, population pairs displaying non‐significant pairwise F_ST_'s according to the permutation test implemented in arlequin v3.5 are linked together with a dashed line. A possible relationship between genetic (Reynolds coefficient *Θ*_w_) and geographic (computed using the R software library “geosphere” (Hijmans 2019a)) distances was assessed through Mantel tests (Smouse, Long, and Sokal 1986) using the R software “ade4” library. Analysis of molecular variance (AMOVA (Excoffier, Smouse, and Quattro 1992)) estimating the 3 fixation indexes Φ_ST_ (percentage of molecular variance explained by inter‐population differentiation), Φ_SC_ (percentage of molecular variance explained by inter‐population differentiation within groups) and Φ_CT_ (percentage of molecular variance explained by inter‐population differentiation between groups) was performed to test possible population structures according to four factors, i.e. geography (using the four pre‐defined geographical regions West Africa, Central Africa, East Africa and North Africa), linguistics (using the three represented linguistic families Niger‐Congo, Afro‐Asiatic and Nilo‐Saharan), lifestyle (using two categories, sedentary and (semi‐)nomadic, i.e. without distinction between semi‐nomadic and nomadic due to low numbers of populations in each category) and strong or weak exposure to *P. falciparum* (using a prevalence threshold ≥ 5% and < 5%, respectively).

#### 2.6.3 Genetic Diversity and Selective Neutrality

Five distinct genetic diversity indexes were estimated for each *HLA* locus, i.e. allelic richness (*ar*) and estimated heterozygosity under HWE (*He*), based on the allele frequency data; and gene diversity (*π*), number of polymorphic sites (*S*) and Tajima's *D* (Tajima 1989), based on the DNA sequence data. To compute *ar*, we used the rarefaction method (El Mousadik and Petit 1996; Hurlbert 1971) estimating the number of alleles that would be detected if all sample sizes were as small as the smallest sample size in this study (i.e., 21, 31, 23 and 32 individuals for *DRB1*, *DQA1*, *DQB1* and *DPB1*, respectively). *He* was computed using the *hla‐net.eu* gene[rate] tools (Nunes et al. 2014) and the 3 molecular indexes *π*, *S* and *D* were computed both on the Whole Exon 2 and independently on the ARS and non‐ARS sub‐regions of each locus, using arlequin v3.5 (Excoffier and Lischer 2010). Statistical comparisons between loci or sequence sub‐regions were performed using Kruskal‐Wallis tests with *fdr* (Benjamini and Hochberg 1995) correction for multiple tests. For these comparisons, *π* and *S* were first divided by the sequence lengths (provided in Table S4) to consider the nucleotide diversity per site (*π*_*n*_) and the number of polymorphic sites per site (*S*_*n*_). To assess selective neutrality on the frequency data, we used the Bootstrapped Ewens‐Watterson‐Slatkin test, hereafter BEWS (Slatkin 1994), where deviation from neutrality was evaluated using a *p*Value interval: if the lowest (resp. highest) bound is below 0.025 (resp. above 0.975), neutrality is rejected towards an excess of heterozygosity (resp. homozygosity). Based on the DNA sequence data, Tajima's neutrality test (Tajima 1989) was performed. As this test is two‐sided, *p*Values were adjusted using “*p*‐Adj = 2*(1 – *p*Value)” for pValues above 0.5 and “*p*‐Adj = 2*pValue” for *p*Values below 0.5. Then, Benjamini‐Hochberg (1995) correction for multiple testing was applied, as implemented in R (Team R core 2013). The ratio of non‐synonymous to synonymous substitutions *dN*/*dS* was computed using mega v10.1.8 (Kumar et al. 2018) on both the Whole Exon 2 sequences and the ARS and non‐ARS sub‐regions and tested for each population. Selective neutrality was also assessed by means of a McDonald‐Kreitman (MK) test performed with dnasp (Rozas et al. 2017) using *HLA* and chimpanzees *Patr* orthologous exon 2 sequences from 25 (*DRB1*), 29 (*DQA1* and *DQB1*) and 20 (*DPB1*) common chimpanzees ( *Pan troglodytes* ) from the Biomedical Primate Research Centre (BPRC, Rijswijk, NL) (Otting, de Groot, and Bontrop 2019). To test for significant differences of *dN, dS* and *dN/dS* between humans and chimpanzees, a bootstrap procedure consisting of 1000 re‐sampling with replacement of the 20 to 23 human values was performed for each locus and nucleotide set (Whole Exon 2 sequences and ARS and non‐ARS sub‐regions). Then, the *dN*, *dS* and *dN/dS* chimpanzee values were compared to these resampled distributions.

#### 2.6.4 HLA Associations With *P. Falciparum* Prevalence

We tested the association between the *HLA* allele frequencies at each locus and the prevalence of the malaria parasite *P. falciparum* for which prevalence data were downloaded from the Malaria Atlas Project (https://data.malariaatlas.org/maps (Bhatt et al. 2015)). As the population samples were collected in the 1990's, prevalence measures for the year 2000 (the oldest available) were used and named *pfpr2000* hereafter. All *pfpr2000* values were extracted from the raster data file using the raster library (Hijmans 2019b) for R. To account for a possible effect of geographic distance on the allele frequency distributions, these distributions were corrected for geography by fitting a linear model explaining the frequencies of each allele as a function of the geographic distance to Addis Ababa (the capital of Ethiopia), and only the residuals (the part of the allele frequencies unexplained by spatial distance) were kept for subsequent analyses. Similarly to what was done by Sanchez‐Mazas et al. (2017), three correlation coefficients (i.e., Pearson cor., Spearman ρ and Kendall τ) were computed between the allele frequencies corrected for geography and the *pfpr2000*, and tested for significance using pValues given by the *cor.test* R function. As *DPB1* displayed both a strong association with *pfpr2000* (at least one of the three estimated correlation coefficients being statistically significant and above 0.5) and a very high frequency in some populations, linear modeling was performed for this locus to assess putative relationships between *pfpr2000* and three statistics known to capture distinct information from the site frequency spectrum, that is, *π*, *S* and Tajima's *D*. Specific relationships between populations, malaria prevalence and *HLA* alleles were also shown by means of a correspondence analysis (*CA*) (Benzécri 1973; Greenacre 1984) performed on *HLA* sequence frequencies using the R package ade4 (Bougeard and Dray 2018; Chessel, Dufour, and Thioulouse 2004; Dray, Dufour, and Chessel 2007; Dray and Dufour 2007).

## 3 Results

### 3.1 Population Differentiations at the Four HLA Class II Loci

**FIGURE 2:**
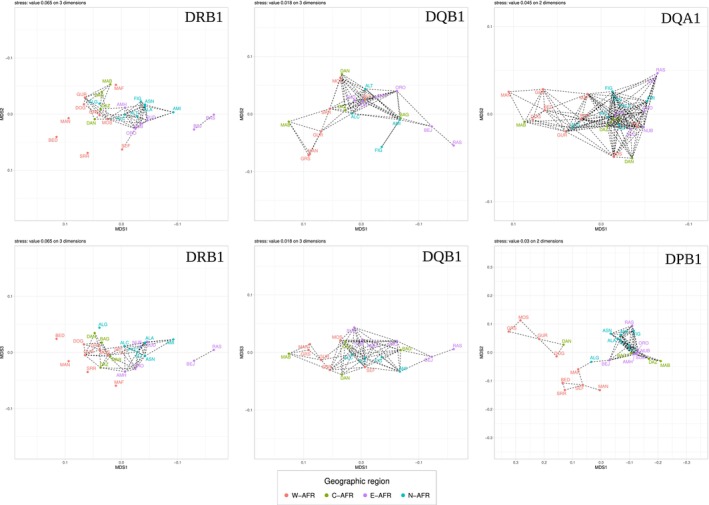
Non‐Metric Multidimensional Scaling analyses (nMDS). Non‐Metric Multidimensional Scaling analyses (nMDS) performed on Reynolds distances *Θ*_*w*_ computed for each locus. The nMDS for *DQA1* and *DPB1* (top right and bottom right, respectively) were computed on two axes, whereas the nMDS for *DRB1* and *DQB1* required a third axis to reduce the Stress value (left for *DRB1* with top: Axes 1 and 2 and bottom: Axes 1 and 3; middle for *DQB1* with top: Axes 1 and 2 and bottom: Axes 1 and 3, respectively). Dashed lines between two populations indicate non‐significant *Θ*_*w*_'s. Gridlines are spaced 0.05 apart in all plots to highlight the scale differences between the loci. Colors correspond to geographic regions, that is, W‐AFR: West Africa; C‐AFR: Central Africa; E‐AFR: East Africa and N‐AFR: North Africa. Short population names correspond to BED: Senegal‐Bedik; MAN: Senegal‐Mandenka; SRR: Senegal‐Serer; SEF: Senegal‐Fulani; MAF: Mali‐Fulani; GUR: BurkinaFaso Gurmantche; GRS: BurkinaFaso‐Gurunsi; MOS: BurkinaFaso‐Mossi; BAG: Chad‐BaggaraArabs; DAN: Chad‐Dangaleat; DAZ: Chad‐Daza; MAB: Chad‐Maba; AMH: Ethiopia‐Amhara‐(Keketeya); ORO: Ethiopia‐Oromo; BEJ: Sudan‐BejaHadendoa; NUB: Sudan‐Nubians; RAS: Sudan‐RashaaydaArabs; SUD: Sudan‐SudaneseArabs; ALC: Algeria‐(Constantine); ALT: Algeria‐(Tamanrasset); AMI: Morocco‐Amazigh‐(Amizmiz); ASN: Morocco‐Amazigh‐(Asni); FIG: Morocco‐Amazigh‐(Figuig).

All populations studied share the great majority of frequent alleles (i.e., alleles reaching a frequency ≥ 10% in at least one population, according to the classification based on (Sanchez‐Mazas et al. 2024)) at all studied *HLA* loci, often with smooth frequency variations within and/or between distinct geographical regions (Table S5, Figure S1). Consequently, many pairs of populations are genetically undifferentiated, as emphasized by dashed lines on the nMDS plots (Figure 2). Similarly, there is no significant correlation between genetic and geographic distances at any locus when we consider the whole geographical area studied (Mantel tests: pValues > 0.05), indicating that a simple model of isolation‐by‐distance does not explain population differentiations at this (sub)continental level.

Instead, some populations stand out in (almost) all nMDS plots, their divergent genetic profiles being a possible consequence of their unique demographic history. First, two Sudanese (semi‐)nomadic populations, RAS and BEJ, show outlier positions at both *DRB1* and *DQB1* and, to a lesser extent, *DQA1*, due to (very) high frequencies of *DRB1#3136* (35%–36%), *DQB1#*2901 (49%–57%) and *DQA1#8* (30%) in both populations, being otherwise close to North (more particularly AMI and FIG) and East (e.g., ORO) Africans. Due to their relatively small size related to their characteristic nomadic lifestyle, their divergence likely results from faster genetic drift compared to neighboring populations. Interestingly, *DRB1#3136*, *DQB1#*2901 and *DQA1#08* include the *HLA* nominal alleles DRB1*07:01, DQB1*02:01/DQB1*02:02 and DQA1*02:01, respectively, which are classified as universally frequent or very frequent at the global scale (Sanchez‐Mazas et al. 2024) and could thus easily drift towards more extreme frequencies in small populations.

Second, West Africans tend to differ from populations living in other geographical regions (mainly at the *DRB1* and *DPB1* loci), but are also genetically diverse (Figure S1, Figure 2). At the *DRB1* locus, the three Senegalese populations –BED, MAN and SRR– show high frequencies of *DRB1#3135*, which is rare or absent in the other populations. At the *DPB1* locus, the Burkinabé populations –MOS, GRS and GUR– and the Malian DOG population exhibit very high frequencies of *DPB1#66*, whereas the Senegalese populations –MAN, BED, SSR and SEF– and the Malian MAF population exhibit very high frequencies of *DPB1#64* (and/or *DPB1#76* in SEF and MAF). These patterns markedly distinguish these two population subgroups both from each other and from (almost) all other African populations. Interestingly, *DPB1* displays a much higher level of differentiation between populations (*Φst* up to 11%) compared to the other loci (*Φst* ~ 4%–6%) (AMOVA, Figure S3). This explains why the scales of the nMDS axes are twice as large for *DPB1* as for the other loci (Figure 2), highlighting the greater differentiation of West Africans for this gene. The AMOVA also reveals that, for all loci except *DQA1*, population differentiations are significantly structured (based on significant *Φ*_*CT*_) by both geographical location (as described above for West Africans) and linguistic family (as explained by MAN, BED, SSR and MAF languages belonging to the Atlantic/Mande branches, and MOS, GRS and GUR languages to the Volta‐Congo branch of the Niger‐Congo phylum, respectively). For *DRB1* and *DQB1*, populations are also significantly structured by lifestyle (as mentioned for the (semi‐)nomadic RAS and BEJ), and for *DPB1*, by exposure to *P. falciparum* (Figure S3).

### 3.2 Genetic Diversity Across the HLA Class II Region

**FIGURE 3:**
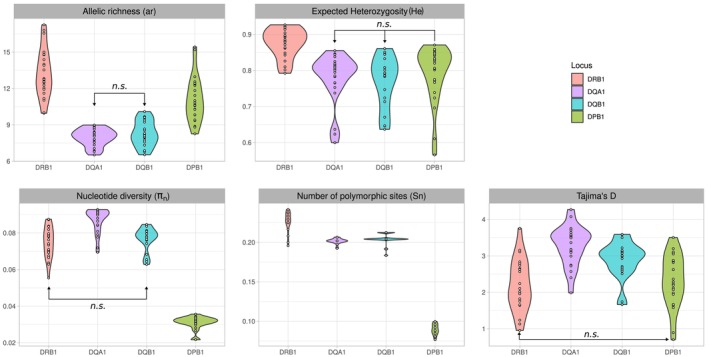
Allelic and molecular diversity indexes. Top: Violin plots of the allelic richness (*ar*) and expected heterozygosity (*He*). Bottom: Violin plots of the average nucleotide diversity per site (*π*_*n*_), average number of polymorphic sites (*S*_*n*_) and Tajima's *D* distributions. All statistics are computed for the four *HLA* loci and 23 populations under study (20 for *DQB1*). Arrows with “*n.s.”* indicate non‐significant differences between two distributions (Kruskal‐Wallis test, α = 0.05, f*dr* correction (Benjamini and Hochberg 1995), see Table 1 for adjusted Pvalues).

Diversity indexes have been estimated at each locus on either allele frequencies (allelic richness *ar* and expected heterozygosity *He*) or on exon 2 sequences (nucleotide diversity *π*_*n*_, number of polymorphic sites *S*_*n*_ and Tajima's *D*, hereafter named together *molecular diversity indexes*). *DRB1* and *DPB1* exhibit significantly higher *ar* compared to *DQA1* and *DQB1*, which do not differ from each other for this statistic (Table 1, Figure 3, Table S7); *He* is also significantly higher for *DRB1* as compared to the other three loci, which do not differ from one another. A few populations exhibit a particularly low *He* (≤ 0.7) at one or several loci, namely the West African GRS (*DQB1*, *DPB1*), MOS (*DPB1*), MAN (*DQA1*, *DQB1*) and BED (*DQA1*), the Central African MAB (*DQA1*, *DQB1*) and the East‐African (semi‐nomadic) RAS (*DQB1*, *DPB1*) (Table S7, Figure S4).

**TABLE 1:** Diversity indexes.

(A) Locus (Exon2)	ar ± σ	He ± σ	π_n_ ± σ	S_n_ ± σ	D ± σ
DRB1	13.2 ± 2.0	0.88 ± 0.04	0.076 ± 0.008	0.228 ± 0.013	2.242 ± 0.677
DQA1	8.4 ± 0.7	0.78 ± 0.07	0.087 ± 0.007	0.201 ± 0.003	3.290 ± 0.558
DQB1	8.3 ± 1.1	0.77 ± 0.07	0.077 ± 0.007	0.205 ± 0.007	2.894 ± 0.511
DPB1	11.1 ± 1.9	0.79 ± 0.08	0.031 ± 0.004	0.090 ± 0.007	2.27 ± 0.749

(B) adj. *p*					
Loci pair	ar	He	π_n_	S_n_	D
DRB1/DPB1	1.86e‐03	2.35e‐05	1.26e‐07	1.06e‐07	7.30E‐01
DQB1/DPB1	8.26e‐06	4.82e‐01	1.26e‐07	9.83e‐08	8.69E‐03
DQA1/DPB1	7.34e‐07	5.70e‐01	1.26e‐07	9.83e‐08	1.49e‐4
DRB1/DQB1	2.55e‐07	1.01e‐05	6.65^e^‐01	1.34e‐05	5.85e‐3
DRB1/DQA1	2.55e‐07	1.12e‐05	8.40^e^‐05	7.28e‐06	1.49e‐4
DQA1/DQB1	6.65e‐01	4.82e‐01	8.40^e^‐05	1.33e‐04	2.23e‐2

*Note:* (A) Average allelic richness (ar), expected heterozygosity (He), nucleotide diversity (pn), number of polymorphic sites per site (Sn) and Tajima's D computed on the 20 populations analyzed for the 4 loci on the whole exon 2. Every value is given with one standard deviation. (B) Adjusted pValues (fdr) of the Kruskal Wallis rank sum tests performed between each pair of loci for each of these five molecular diversity indexes.

Regarding the molecular diversity indexes, remarkably low *π*_*n*_ and *S*_*n*_ are observed for *DPB1* compared to the other three loci (Table 1, Figure 3). Tajima's *D* is positive (with overall highest values for *DQA1*) and significant for most of the populations (although most often so at *DQA1*–95%‐ and *DQB1*–90%‐ compared to *DPB1*–70%‐ and *DRB1–*52%‐, Table 2). This primarily indicates balancing selection at all genes studied. When considering ARS and non‐ARS sub‐regions, the molecular diversity indexes are significantly higher for ARS than for non‐ARS across all loci (especially *DRB1* and *DQB1*), except for Tajima's *D* at *DQA1* (Figure S5, Table S8).

**TABLE 2:** Bootstrapped‐Ewens‐Watterson‐Slatkin (BEWS) and Tajima's D neutrality tests.

Region	Population	DRB1 (23 populations)				DQA1 (26 populations)				DQB1 (20 populations)				DPB1 (26 populations)			
		BEWS	Tajima's D			BEWS	Tajima's D			BEWS	Tajima's D			BEWS	Tajima's D		
			Whole Exon 2	ARS	Non‐ARS		Whole Exon 2	ARS	Non‐ARS		Whole Exon 2	ARS	Non‐ARS		Whole Exon 2	ARS	Non‐ARS
W‐AFR	BED	0.78–0.95	0.95	1.39	0.25	0.74–0.88	2.72*	2.74*	2.44*					0.26–0.71	3.1**	3.03**	2.54*
W‐AFR	DOG					0.03–0.12	3.25***	2.85**	3.06**					0.31–0.55	2.93**	3.15**	2.25*
W‐AFR	MAN	**0.02**–0.13	2.02	2.42*	1.19	0.20–0.44	2.99*	2.78*	2.72*	0.56–0.85	3.09*	3.14**	2.44*	0.23–0.53	3.5**	4.28**	2.31*
W‐AFR	SRR	0.04–0.64	2.24*	2.62*	1.46	0.22–0.58	3.49***	3.44**	2.95*	0.58–0.91	3.07**	3.15**	2.51*	0.31–0.75	2.11*	2.95*	1.3
W‐AFR	SEF	0.05–0.54	2.65*	2.6*	2.36*	**0.00**–0.09	3.71***	3.24**	3.57**	0.17–0.57	3.59**	3.7**	2.89*	0.06–0.48	2.36*	3.38**	1.47
W‐AFR	MAF	0.38–**1.00**	1.82	2.05	1.28	0.05–0.51	3.5***	3.28**	3.47**					0.14–0.54	2.62*	3.21*	1.88
W‐AFR	GUR	**0.00**–0.42	1.13	1.28	0.78	0.23–0.77	2.57*	2.37*	2.43*	0.33–0.74	2.51*	2.56*	2.1*	0.80–0.96	1.64	1.59	1.41
W‐AFR	GRS	**0.00**–0.44	1.64	1.95	1.06	0.26–0.80	2.4*	2.38*	2.17*	0.47–0.88	1.66	1.75	1.31	0.99–**0.99**	0.7	0.69	0.59
W‐AFR	MOS	**0.00**–0.28	1.66	1.89	1.15	0.10–0.62	2.75*	2.34*	2.71*	**0.02**–0.50	2.63*	2.71*	2.14*	0.90–**0.98**	0.9	0.49	1.01
C‐AFR	BAG	**0.01**–0.47	1.96	2.21*	1.38	0.08–0.62	3.54***	3.14**	3.18**	0.30–0.78	3.14**	3.49**	2.26*	0.53–0.92	2.82*	2.89*	2.29*
C‐AFR	DAN	**0.01**–0.35	1.66	1.7	1.39	0.15–0.55	3.09**	2.57*	2.87**	0.09–0.50	3.22**	3.33**	2.58*	**0.02**–0.44	2.86*	2.57*	2.53*
C‐AFR	DAZ	0.62–0.93	1.24	1.61	0.61	0.09–0.53	3.17**	2.78*	3.29**	0.17–0.56	2.93**	3.13**	2.22*	0.62–0.93	1.58	2.44*	0.91
C‐AFR	MAB	0.41–0.92	1.96	2.2*	1.43	0.89–**0.98**	1.99	1.64	1.98	0.80–0.97	1.74	1.87	1.31	0.36–0.81	1.67	1.82	1.29
E‐AFR	AMH	**0.01**–0.28	2.74*	2.94*	2.03	**0.01**–0.13	4.08***	3.52**	3.92***	**0.00**–0.10	3.58**	3.69**	2.87*	0.34–0.77	3.09*	3.2**	2.46*
E‐AFR	ORO	0.20–0.95	1.76	2.14*	1.1	0.14–0.80	3.17**	2.85*	3.04**	0.32–0.96	3.14**	3.14***	2.7*	0.53–0.91	2.23*	2.24*	1.9
E‐AFR	BEJ	0.60–0.91	3.16**	3.82**	1.94	0.12–0.69	3.57***	3.35**	3.11**	0.37–0.76	3.01**	3.08**	2.47*	0.04–0.48	3.2**	3.34**	2.58*
E‐AFR	NUB	**0.02**–0.66	2.63*	2.92*	1.93	**0.00**–0.11	3.64***	3.17**	3.31**	0.06–0.50	3**	3.39**	2.09	0.25–0.72	2.63*	2.78*	2.08
E‐AFR	RAS	0.95–**0.99**	3.75**	4.33***	2.45*	0.09–0.51	3.55**	3.42**	3.25**	0.67–0.89	2.58*	2.65*	2.09*	0.96–0.97	3.05*	2.85*	2.59*
E‐AFR	SUD	**0.00**–0.67	2.57*	3.03*	1.73	**0.01**–0.22	3.58***	3.04**	3.3**	**0.02**–0.35	2.67*	3.06**	1.81	0.60–0.96	2.07*	2.82*	1.39
N‐AFR	ALA					**0.02**–0.13	4.40***	3.77***	4.00***					0.08–0.50	2.90**	3.51***	2.06*
N‐AFR	ALC	0.10–0.84	2.11*	2.19*	1.73	0.14–0.57	3.36***	2.88*	3.3**	0.13–0.60	2.99**	3.31**	2.17*	0.39–0.85	1.98	2.82*	1.23
N‐AFR	ALG					0.06–0.22	3.44***	2.95**	3.32***					**0.00**–0.04	3.40***	3.63***	2.68**
N‐AFR	ALT	**0.00**–0.54	2.02*	2.36*	1.37	**0.02**–0.31	3.12**	2.79*	3**	**0.01**–0.35	3.12**	3.15**	2.65*	0.14–0.76	1.94	2.31*	1.47
N‐AFR	AMI	0.30–0.83	3.11**	3.77**	1.94	0.05–0.37	3.75***	3.33**	3.59**	0.40–0.93	2.71*	3.01**	2	0.77–0.96	2.19*	3.03*	1.4
N‐AFR	ASN	0.27–0.93	2.79*	3.19**	1.95	0.08–0.48	3.52**	3.35**	3.46**					0.28–0.77	2.29*	2.64*	1.72
N‐AFR	FIG	0.19–0.83	2.84*	3.02*	2.21*	0.03–0.20	4.27***	3.75**	4.06***	0.13–0.54	3.5**	3.58**	2.82*	0.38–0.69	2.88*	3.05*	2.2
Tot‐neut‐reject (%)		12 (52%)	12 (52%)	16 (70%)	3 (13%)	7 (27%)	22 (95%)	22 (95%)	22 (95%)	4 (20%)	18 (90%)	18 (90%)	15 (75%)	4 (15%)	16 (70%)	19 (83%)	7 (30%)
Het‐excess (%)		10 (43%)				6 (23%)				4 (20%)				2 (8%)			
Hom‐excess (%)		2 (9%)				1 (4%)				0 (0%)				2 (8%)			

*Note:* Results of neutrality tests performed on the four loci for each population, except in case of HWE deviation (DOG, ALA, ALG at DRB1 and DQB1) or insufficient sample size (BED, MAF, ASN at DQB1). Regions correspond to W‐AFR: West Africa; C‐AFR: Central Africa; E‐AFR: East‐Africa and N‐AFR: North Africa. See Table S1 for short names correspondences. BEWS is the pValue interval of the Bootstrapped Ewens‐Watterson‐Slatkin Test: if the lowest (resp. highest) bound is below 0.025 (resp. above 0.975), the neutrality is rejected towards an excess of heterozygotes (resp. homozygotes) and the *p*Value is indicated with bold font. Tajima's D is given according to 3 nucleotides sets, Whole Exon 2: the whole exon 2 sequence; ARS: only codons coding for the Antigen Recognition site; non‐ARS: only codons not coding for the Antigen Recognition Site. Tajima's D significance (after fdr correction (Benjamini and Hochberg 1995)) corresponds to: ‘*’ < 0.05, ‘**’ < 0.01 and ‘***’ < 0.001. Tot‐neut‐reject (%): total number (and percentage) of populations rejecting neutrality for each test; Het‐excess (%) and Hom‐excess (%): number (and percentage) of rejections towards an excess of heterozygotes and homozygotes, respectively.

### 3.3 Linkage Disequilibrium Between the Four HLA Class II Genes

Strong linkage disequilibrium is observed between *DRB1*, *DQA1* and *DQB1*, with 80% to 95% of the populations showing significant *GLD* between these loci, and 6% to 10% of their individual haplotypes being in significant *LD* (Figure S2, Table S6). As expected from the shorter physical distance that separates them relatively to *DRB1*, the strongest linkage disequilibrium among these three genes lies between *DQA1* and *DQB1*, which encode the α and β chains of the *HLA‐DQ* molecule, respectively. In contrast, only 30%–35% of populations display significant *GLD* between *DPB1* and the other three loci, and only 1% of haplotypes involving a *DPB1* allele are in significant *LD*.

### 3.4 Deviations From Selective Neutrality

According to the BEWS neutrality test, *DRB1* exhibits a substantial number of significant rejections towards an excess of heterozygosity (43%, compared to ~20% at *DQA1* and *DQB1* and only 4% at *DPB1*, Table 2), which parallels the high and significant *He* observed at this locus (Table 1 and Figure 3). This supports balancing selection in the form of heterozygosity advantage affecting *DRB1* and, to a lesser degree, other *HLA* class II genes (Buhler and Sanchez‐Mazas 2011; Solberg et al. 2008). Rejections of neutrality towards an excess of homozygosity (explained either by directional selection or genetic drift) are also observed in a few populations, namely the West African GRS and MOS (at *DPB1*), the West African MAF (at *DRB1*) and the (semi‐)nomadic RAS (at both *DRB1* and *DPB1*), in addition to the Central African MAB (at both *DQA1* and *DQB1*). As mentioned above, the great majority of populations tested exhibit significant positive Tajima's *D* values at ARS codons of all *HLA* loci (Table 2), suggesting an effect of balancing selection more specifically related to the peptide‐binding function of the *HLA* molecules. This is not the case, however, for the West African GUR, GRS and MOS populations, at *DRB1* and *DPB1*, and the Central African MAB population, at *DQA1*, *DQB1* and *DPB1*. In addition, significant Tajima's *D* are also observed at non‐ARS codons of *DQA1* and *DQB1* for a majority (75%–95%) of populations (Table 2).

Molecular comparisons between humans and chimpanzees reveal no fixed nucleotide differences between the two species at any of the four *HLA* genes, indicating an absence of species‐specific substitutions at these loci and making it impossible to perform the *MK* test (Table 3). This supports an excess of within‐species polymorphism compared to between‐species divergence at the four *MHC* loci, as expected under balancing selection. *DRB1* and *DQB1* exhibit the greatest *dN* for ARS codons in humans, which is not seen at *DQB1* in chimpanzees. By contrast, *dS* is much greater at *DQA1* and null at *DPB1* for ARS codons in the two species. Overall, *dN/dS* is greater at *DPB1* in humans (Figure S6, Table S9), which goes alongside the greater proportion of non‐synonymous differences at this locus between the two species (Table 3).

**TABLE 3:** Contingency tables for McDonald‐Kreitman test.

DRB1			DQA1		
	Fixed	Polymorphic		Fixed	Polymorphic
Synonymous	0	11	Synonymous	0	17
Non‐synonymous	0	31	Non‐synonymous	0	23
Significance	n.a		Significance	n.a	

DQB1			DPB1		
	Fixed	Polymorphic		Fixed	Polymorphic
Synonymous	0	17	Synonymous	0	3
Non‐synonymous	0	37	Non‐synonymous	0	30
Significance	n.a		Significance	n.a	

*Note:* Contingency tables for McDonald‐Kreitman test (MKT) based on HLA (23 populations except for DQB1 tested for 20 populations) and Patr data. Each table gives, for one locus, the number of Synonymous and Non‐Synonymous differences, that are either fixed or polymorphic (a fixed nucleotide site between species is a site at which all sequences in one species contain nucleotide variants that are not in the second species). The number of HLA/Patr sequences used for computations are DRB1: 2392/50; DQA1: 2492/58; DQB1: 2078/58 and DPB1: 2496/40. Significance could not be assessed (n.a) due to the lack of fixed substitutions in each species.

### 3.5 Associations With Plasmodium Falciparum Prevalence

**FIGURE 4:**
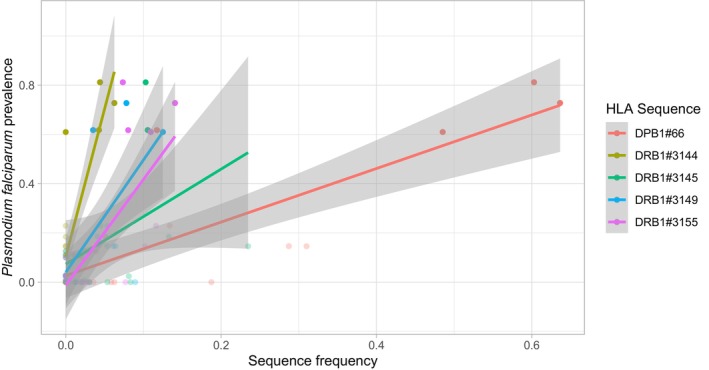
*HLA* sequences associated with *Plasmodium falciparum* prevalence. For the 5 *HLA* alleles identified as strongly correlated with *Plasmodium falciparum* prevalence (*pfpr2000*, Table S10.1), relationship between the observed frequency in each of the 23 population (20 for *DQB1*) and *pfpr2000* at their sampling location. Three of these alleles, namely DPB1#66, DRB1#3144 and DRB1#3155, remain significantly associated with *pfpr2000* after *fdr* correction for multiple testing. See Table S3 for the correspondence between sequence names and nominal *HLA* alleles.

**FIGURE 5:**
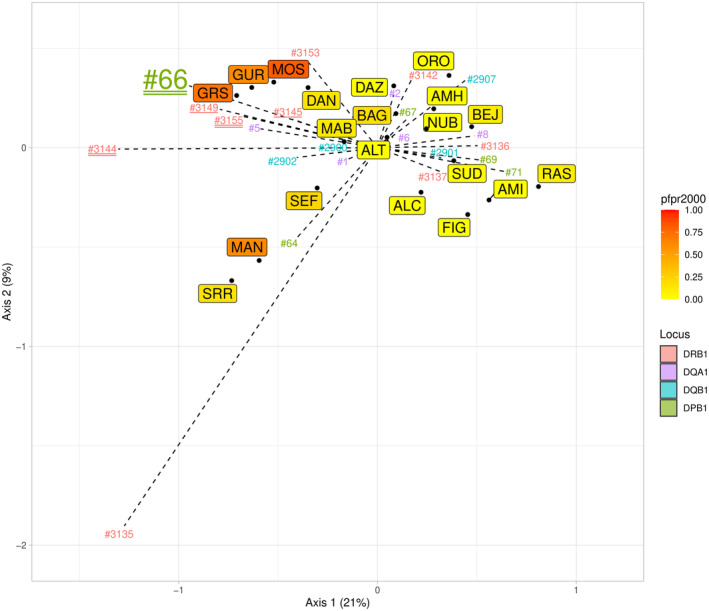
Correspondence analysis. Correspondence analysis (axes 1 and 2) performed on the allele frequencies of 20 populations (BED, MAF and ASN were excluded due to the lack of data at *DQB1*). Axes 1 and 2 capture 21% and 9% of the total variance, respectively. The continuous color scale *pfpr2000* corresponds to *Plasmodium falciparum* prevalence in year 2000 at the sampling locations of each population represented. For the sake of clarity, allele names are reported without the locus prefix but are colored according to the locus (pink: *DRB1*; violet: *DQA1*; blue: *DQB1*; green: *DPB1*). All alleles with frequencies above 20% in at least one population as well as all alleles displaying a significant association with *pfpr2000* either before (underlined) or after (double underlined) *fdr* correction for multiple testing are represented. Allele DPB1#66 is shown in a larger format because of its very high frequencies in some populations. See Table S3 for the correspondence between sequence names and nominal *HLA* alleles.

Five *HLA* alleles (*DPB1#*66, *DRB1#3144*, *DRB1#3145*, *DRB1#3149*, and *DRB1#3155*) show strong associations with *pfpr2000*, that is, at least one of the three estimated correlation coefficients being statistically significant and above 0.5 (Table S10.1, Figure 4). However, only three of these alleles (*DPB1#66, DRB1#3144* and *DRB1#3155*) still display significant associations after correction for multiple testing, and only one of them (*DPB1#66*) exhibits very high frequencies in West African populations strongly exposed to malaria (49%–64% in the MOS, GUR and GRS, respectively, compared to maximum frequencies of 6%–14% for the other two alleles, Table S10.2). This suggests a strong significant positive selection for one *DPB1* allele, *DPB1#66*, and a significant, though more moderate, positive selection for two *DRB1* alleles, *DRB1#3144* and *DRB1#3155*, in response to malaria. These results are well illustrated by the *CA* plot shown in Figure 5. This graph also reveals that, in contrast to MOS, GUR and GRS, the West African Senegalese SRR and MAN populations (and, to a lesser extent, SEF) are characterized by high frequencies of two alleles, *DPB1#64* and *DRB1#3135*, that are not associated with *pfpr2000*. Interestingly, these two sequences include the *HLA* nominal alleles DPB1*17:01 and DRB1*13:04, respectively, which are classified as locally (very) frequent in sub‐Saharan Africa (Sanchez‐Mazas et al. 2024) and were tentatively suggested to confer immune protection to other pathogenic strains in West Africa (Goeury, Creary, Brunet, et al. 2018).

The hypothesis of positive selection driving the evolution of *DPB1* in a malaria‐endemic environment is supported by the *AMOVA* analysis (Figure S3) revealing, as mentioned above, that *DPB1* is the only locus showing a significant genetic structure among population groups based on exposure to *P. falciparum* (*Φ*_*CT*_ = 0.046, *p* < 0.05).

As the *DPB1* locus revealed the strongest signals of association with *P. falciparum* prevalence, the hypothesis of pathogen‐driven selection was more directly tested by applying a linear model. To do so, the three diversity indices *Θ*_*π*_, *Θ*_*S*_ (i.e., the population parameter *Θ* estimated either by the mean number of pairwise nucleotide differences between sequences ‐*π*‐ or by the number of segregating sites ‐S‐, respectively) and Tajima's *D* (≅ *Θ*_*π*_—*Θ*_*S*_) were first calculated on the two sets of nucleotides ARS and non‐ARS of *DPB1* for each of the 20 populations retained for the analyses. Linear models were then fitted to investigate their relationships with *pfpr2000*. Equations ((1), (2), (3)), indicated below, show that three significant negative relationships are found, namely for Tajima's *D* on ARS codons and for *Θ*_*π*_ on both ARS and non‐ARS codons:

(1) DARS=−1.67×pfpr2000+2.90

(2)

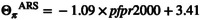



(3)

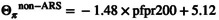

Strong significant associations (*p* << 0.01) are observed for *Θ*_*π*_^*ARS*^ and *Θ*_*π*_^*non‐ARS*^, with the *R* ^*2*^ coefficients also indicating that *pfpr2000* alone explains a great proportion of the observed molecular variation at *DPB1* exon 2 (*R* ^2^ = 39% and 45% for *Θ*_*π*_^*ARS*^ and *Θ*_*π*_^*non‐ARS*^, respectively, Table S10.3).

## 4 Discussion

Africa is considered today as the homeland of our species, *Homo sapiens* , with a fully modern anatomy emerging between 200′000 and 300′000 years ago, according to the fossil record (Hublin et al. 2017; Vidal et al. 2022). Modern humans migrated to Eurasia perhaps as early as 210′000 years ago (Harvati et al. 2019), although successful establishment out of Africa presumably occurred much later (Bergström et al. 2021; López, van Dorp, and Hellenthal 2016). Populations migrating out of Africa likely underwent multiple founder effects that led to the loss of a great part of the genetic diversity present in the ancestral African population, explaining why Africa currently holds the greatest genetic diversity (The 1000 Genomes Project Consortium 2015). In‐depth studies of this remarkable African diversity have been undertaken to enhance our understanding on how human populations expanded across this continent and adapted to its variable environments (Bird et al. 2023; Černý et al. 2018; Fan et al. 2023; Wonkam and Adeyemo 2023). However, more investigations of this diversity are needed, particularly to address biomedical issues (Sirugo, Williams, and Tishkoff 2019). Indeed, the burden of infectious diseases is still dramatically high in Africa (Boutayeb 2010; World Health Organization 2023), and only thorough analyses of the genetic background of African populations may allow unraveling genetically‐based disease susceptibilities, including those related to *HLA* genes (Fortes‐Lima et al. 2022; Sanchez‐Mazas 2020), and the development of targeted health care solutions, such as vaccines.

To date, however, our knowledge of *HLA* molecular diversity in African populations remains limited, particularly for *HLA* class II genes, which play a major role in parasitic infections. In this context, we sought to explore the molecular variation of these polymorphisms across Africa to identify, among other mechanisms shaping their diversity, putative associations with malaria, the most devastating parasitic disease in Africa (World Health Organization 2023; Varo, Chaccour, and Bassat 2020). The sequencing of four *HLA* class II genes in more than 1000 individuals from 23 populations across Western (Senegal, Mali, Burkina Faso), Central (Chad), Eastern (Ethiopia, Sudan) and Northern (Algeria and Morocco) Africa evidenced significant signals of both balancing and pathogen‐driven selection acting on this genomic region, especially DPB1, the most centromeric gene.

### 4.1 Demographic and Geographic Signals Revealed by HLA Class II Genetic Patterns

The study of *HLA* class II molecular variation across Africa reveals distinct patterns, likely shaped (in part) by specific demographic histories or geographic differentiations of the populations studied.

Firstly, several (semi‐)nomadic populations that are included in our dataset stand out in various analyses. Consistent with previous observations made on *HLA* class I loci (Sanchez‐Mazas et al. 2017), the pastoral Beja (BEJ) and Rashaayda (RAS) populations of Sudan (the latter being descendants of a population that migrated from the Arabian peninsula in the 19th century) exhibit several alleles at very high frequencies that make them appear as outliers on the nMDS plots (Figure 2). These observations highlight the significant genetic structure based on lifestyle found for *DRB1* and *DQB1* (AMOVA, *Φ*_*CT*_ of 0.016 and 0.023, respectively, *p* < 0.05, Figure S3). RAS, along with the nomadic Fulani from Mali (MAF), also exhibit an excess of homozygosity at *DRB1*, although this locus displays a significantly greater heterozygosity than the other class II genes (Table 1, Figure 3) and deviates from neutrality towards an excess of heterozygosity in many populations (BEWS test, Table 2). BEJ, RAS and two other (semi‐)nomadic populations, the Amazigh from Morocco (FIG) and the Fulani from Senegal (SEF), also exhibit significant global linkage disequilibrium (*GLD*) at all contiguous *HLA* class II loci pairs (Figure S2). This is an unexpected finding for *DQB1* ~ *DPB1*, as *DPB1* is genetically distant from the other loci due to one or several recombination hotspots near the *TAP2* genes (Cullen et al. 1997; Jeffreys, Kauppi, and Neumann 2001; Martin, Mann, and Carrington 1995). All these (i.e., some extreme allele frequencies, reduced genetic diversity and elevated linkage disequilibrium) probably result from small populations undergoing rapid genetic drift.

Secondly, West African populations show marked differentiations in the four *HLA* loci studied, particularly for *DRB1* and *DPB1*, where they often appear as outliers on the nMDS plots and show significant differentiations (*F*_*st*_'s) from populations of other regions (Figure 2). The greatest differentiation of West Africans is found for *DPB1*, which clearly distinguishes two population subgroups and displays greater *Φ*_*CT*_'s than the other loci when genetic structure is tested in relation to either geographical regions or linguistic families (Figure S3). These two West African subgroups are indeed represented, on the one hand, by the Burkinabé Gurunsi (GRS), Gurmantché (GUR) and Mossi (MOS) and the Malian Dogon (DOG), all of whom speak languages of the Volta‐Congo branch of the Niger‐Congo phylum; and, on the other hand, by the Senegalese Mandenka (MAN), Serer (SER) Bedik (BED) and Fulani (SEF and MAF, from Senegal and Mali, respectively), who speak languages of the Mandé (for MAN) and Atlantic (for the others) branches of Niger‐Congo (Eberhard, Gary, and Charles 2019). Within the latter subgroup, MAN, SER and BED also differ at *DRB1* from all other African populations due to a high frequency of *DRB1#3135*, a sequence corresponding to the nominal allele HLA‐DRB1*13:04 that likely arose through gene conversion (Goeury, Creary, Brunet, et al. 2018) and is frequent in an area corresponding to the Mande and Atlantic branches of Niger‐Congo (from Senegal and Gambia to Sierra Leone). Interestingly, at a wider genomic level (based on 2.2 million SNPs), West Africans also present varying proportions of two genomic components between West Atlantic populations (from Senegal, Gambian and Sierra Leone), on one side, and West‐Central populations (from Burkina Faso and Nigeria), on the other side (Triska et al. 2015). Overall, these results suggest that West Africans underwent substantial genetic differentiations during their geographical dispersal, which also parallels the linguistic diversification of Niger‐Congo languages into Mande, Atlantic and Volta‐Congo subfamilies (Eberhard, Gary, and Charles 2019).

In addition, as we previously demonstrated that the *HLA‐B* locus was significantly driven by positive selection in response to malaria in this geographical region (Sanchez‐Mazas et al. 2017; Černý et al. 2018), we investigated possible effects of natural selection on the *HLA* class II genes.

### 4.2 Evidence of Different Selective Regimes for the Four HLA Class II Loci

Models of balancing selection in the form of heterozygote advantage (Doherty and Zinkernagel 1975) or divergent allele advantage (which considers variable fitness of heterozygotes depending on the molecular distance of the carried alleles) (Wakeland et al. 1990; Pierini and Lenz 2018) are generally accepted as the main evolutionary processes explaining the high levels of heterozygosity usually observed in the *HLA* (more generally *MHC*) region. This study supports this as it shows clear signals of balancing selection, although not to the same extent, at the four *HLA* class II loci analyzed (BEWS and Tajima's *D* tests, Table 2, Figure 3). Balancing selection probably explains the lack of genetic differentiation (i.e., non‐significant *Fst*'s, Figure 2) between many of the populations included in our study despite the extensive geographical area covered, as this selective regime is (although not in all cases (Brandt et al. 2018)) expected to maintain shared polymorphisms among populations. However, our results also suggest that the four *HLA* class II loci have evolved under varying or additional types of natural selection, as discussed below.

*DRB1* and *DQB1* are similar in several molecular aspects, that is, they share comparable nucleotide diversities *π*_*n*_ (overall, but also at either ARS or non‐ARS codons, Figure 3, Table 1B, Figure S5, Table S8C) and display both much greater *π*_*n*_ and much greater non‐synonymous substitution rates (*dN*) at ARS than at non‐ARS codons, compared to the other loci (Figure S6, Table S9.1). As also observed for *HLA* class I (Bitarello, Francisco, and Meyer 2016), *DRB1* and *DQB1* molecules have accumulated a very substantial amount of amino acid variation at their peptide‐binding sites, supporting heterozygous advantage or divergent allele advantage modes of evolution.

By contrast, *DQA1*—the only locus encoding the α‐chain of the *HLA* molecule in this study—is very peculiar in that it exhibits a high rate of synonymous substitutions *dS* (mostly at ARS codons), resulting in a very low *dN/dS* ratio (< 1) compared to the other genes (Figure S6, Tables S9.2, S9.3), but also higher proportions of significant Tajima's *D*, both at ARS and non‐ARS codons (Table 2). These findings indicate that *DQA1* evolved according to different mechanisms compared to the *HLA* genes encoding the β‐chains, even though both α and β chains participate to the peptide‐binding pockets of the *HLA* class II molecules and are possible targets of natural selection (Hammer et al. 2015; Sarri et al. 2018; Valencia et al. 2022; Zerva et al. 1996). Interestingly, by comparing the genomes of 50 First Nations from Canada (25 modern and 25 predating European colonization), Lindo et al. (2016) suggested a possible change in the selection regime for *DQA1*, shifting from positive to negative selection following European colonization. Our results are compatible with *DQA1* undergoing greater constraints reducing the functional diversity (i.e., *dN* vs *dS*) of the α‐chain. Peculiar selective pressures are also known to affect the generation of *HLA‐DQ* αβ heterodimers, some allele combinations leading to unstable molecules (Kwok, Nepom, and Raymond 1995; Kwok et al. 1993).

Interestingly, *dN/dS* is very low at both *DQA1* and *DQB1* in chimpanzees, and not only at *DQA1* as is the case in humans (Figure S6, Table S9.3). Therefore, as an alternative to a shift affecting the mode of evolution of *DQA1*, as discussed above, an earlier negative selection acting on *DQB1* in humans might have been relaxed through time towards a greater advantage of diversity, explaining the pronounced *dN* difference for the ARS codons of this locus between the two species (Figure S6, Table S9.1). A great divergence of *Patr‐DQB1* from its ortholog in humans was previously observed based on several genetic diversity indexes (Vangenot et al. 2020). A tentative hypothesis is that an increase in parasitic diseases, particularly since the advent of agriculture in the Neolithic period (Carter and Mendis 2002), drove the diversification of some *HLA* class II genes that were still poorly diversified (i.e., *DQB1*) in humans, as they expanded demographically. Therefore, although the overall evolutionary patterns of the *MHC* region appear to be conserved in humans and chimpanzees (Vangenot et al. 2020) with more polymorphism than divergence between the two species as expected under balancing selection, some molecular differences may represent relevant signatures of their distinct demographic histories, as was proposed for *MHC* class I genes in relation to past bottlenecks in chimpanzees (Otting, de Groot, and Bontrop 2019; de Groot et al. 2002).

However, a particularly important contribution of our work is the demonstration of substantial positive selection shaping the molecular diversity of *DPB1* and, to a lesser extent, *DRB1*, in addition to balancing selection. The frequencies of 5 *HLA* alleles of these two loci are found to be significantly associated with *Plasmodium falciparum* prevalence, even when taking geographic distance into account. For three of them, *DPB1#66, DRB1#3144* and *DRB1#3155*, these associations remain significant after correction for multiple testing (Table S10.1). *DPB1#66*, corresponding to the *HLA* nominal allele DPB1*01:01, reaches particularly high frequencies in Volta‐Congo‐speaking populations from Burkina Faso (Figure 4, Figure S1), leading to a significant excess of homozygosity at the *DPB1* locus in two of these populations (Table 2). We hypothesize that this allele became highly prevalent due to positive selection driven by its protective effect against malaria. In agreement with this hypothesis, May et al. found a higher frequency of HLA‐DPB1*01:01 among Gabonese individuals expressing a mild form of malaria compared to those individuals with a severe form (May et al. 1999). A putative pathogen‐driven positive selection acting on *HLA‐DPB1* is also consistent with it being the only locus showing a significant *Φ*_*CT*_ (of 0.046, *p* < 0.05) when testing *HLA* genetic structure in relation to exposure to *P. falciparum* (Figure S3). This finding aligns with the results of the linear modeling, which reveals that *P. falciparum* prevalence significantly explains a very large part of *DPB1* molecular diversity (Table S10.3). This challenges the conclusions drawn by other studies suggesting that the *DPB1* locus is neutral (or affected by ancient balancing selection (Buhler and Sanchez‐Mazas 2011)) in view of its L‐shaped allele frequency distributions in most populations and very few significant rejections of neutrality (Buhler and Sanchez‐Mazas 2011; Solberg et al. 2008; Sanchez‐Mazas 2001). By contrast, the frequencies of *DRB1#3144* and *DRB1#3155*, which correspond to the *HLA* nominal alleles DRB1*08:06 and DRB1*11:02, would have increased more smoothly, e.g. from the standing genetic variation at the onset of the disease, as was suggested for *HLA‐B* alleles (Sanchez‐Mazas et al. 2017). Interestingly, these two alleles were considered as donor and recipient, respectively, in a gene conversion event that gave rise to DRB1*13:04 (Goeury, Creary, Brunet, et al. 2018; Hill et al. 1992). This allele is currently the most frequent *DRB1* allele in the Mande and Atlantic‐speaking populations of West Africa. This was proposed to be the result of recent positive selection (Goeury, Creary, Brunet, et al. 2018; Hill et al. 1992), although no specific selective advantage of this allele has yet been identified. Both functional studies and epidemiological surveys are essential to gain a better understanding of the complex interplay between *HLA* variants and diseases, particularly in pathogen‐rich environments such as those of sub‐Saharan Africa.

As 11 population samples (namely GUR, GRS, MOS, BAG, DAN, DAZ, MAB, BEJ, NUB, RAS, SUD) analyzed in the present work were also included in our previous *HLA* class I study (Sanchez‐Mazas et al. 2017), we investigated whether the presumed protective alleles that we identified in the two studies (formerly B*53:01, and now DPB1*01:01, DRB1*08:06 and DRB1*11:02) belonged to an extended *HLA* class I—class II haplotype. Interestingly, we identified that, of the total number of 54 individuals carrying B*53:01 in our previous study, 32 also carry DPB1*01:01, representing a substantial proportion (59%) of co‐occurrences. However, among these, the highest co‐occurrences with alleles at the other class II loci (which lie between *HLA‐B* and *DPB1*) were observed for 21 DQA1*05:05, 16 DQB1*03:01/DQB1*03:19 and 6 DRB1*11:01 individuals and even less so for DRB1*08:06 and DRB1*11:02 (the two *DRB1* alleles significantly associated with *P. falciparum* prevalence), consequently ruling out the existence of an extended *HLA* class I—class II protective haplotype. Furthermore, in the present study, we do not find any significant linkage disequilibrium including *DPB1#66* (DPB1*01:01) across the four *HLA* class II loci (Table S6), which is not surprising given the recombination hot spots that lie between *DPB1* and the other *HLA* genes.

Instead, HLA‐B*53:01, DPB1*01:01, DRB1*08:06 and DRB1*11:02 likely confer malaria protection in an independent and complementary way. Consistent with this hypothesis, it has been proposed that *HLA‐B*‐mediated protection to malaria acts via immune inhibition through *KIR* receptors (*B53* being a *Bw4* inhibitory ligand to *KIR*) rather than via (or in addition to) pre‐erythrocytic T‐cell immune responses (Digitale et al. 2021; Norman et al. 2013), whereas *HLA* class II molecules are thought to trigger antibody‐restricted immunity across all stages of the parasitic infection (Fiorillo et al. 2017; Medhasi and Chantratita 2022; Meyer et al. 2006). Likewise, among *HLA* class II genes, *DPB1* and *DRB1* may play complementary roles, e.g. by presenting distinct peptide repertoires to T‐cells. Based on the *generalist* versus *specialist* view of *MHC*‐mediated protection to infectious pathogens (Kaufman 2018, 2020), DPB1*01:01 may be more fastidious (i.e., binding a narrow range of peptides conferring strong protection) and DRB1*08:06 and DRB1*11:02 more promiscuous (i.e., binding a wider range of peptides conferring weaker protection) in malaria resistance. This is supported by the observed allele frequencies (high for DPB1*01:01, low for DRB1*08:06 and DRB1*11:02, Figure 4), the very low nucleotide diversity of *DPB1* (Figure 3), which indicates that this locus encodes functionally very similar molecules, and the higher heterozygosity of *DRB1* (Table 1, Figure 3) often deviating from neutral expectations (Table 2), as expected under the heterozygote advantage model which promotes the preservation of many different alleles in populations (Penn, Damjanovich, and Potts 2002). Such functional complementarity between molecules of distinct *HLA* genes had previously been suggested for *HLA* class I loci under the model of *joint divergent asymmetric selection* based on peptide‐binding analyses (Buhler, Nunes, and Sanchez‐Mazas 2016; Di et al. 2020) using NetMHCpan bioinformatic tools (Reynisson et al. 2020). This model predicts that alleles from the three genes *HLA‐A*, *HLA‐B* and *HLA‐C*, by binding complementary peptide repertoires, would together confer an equivalent immune potential in all human populations, whereas alleles from a single *HLA* class I locus alone would not. An interesting extension of the present study would thus be to analyze *HLA* class II genes using a similar bioinformatic approach. This would require the completion of our current data sets for loci encoding the α‐chains of the *HLA* class II molecules (e.g., *DRA, DPA*) and the use of efficient peptide‐binding prediction tools for HLA class II αβ heterodimers, whose accuracy is only recently improving (Racle et al. 2023; Wang et al. 2024). With regard to immune responses against malaria, it is also understood that a wide range of non‐*HLA* variants of the genome, such as markers involved in hemoglobin disorders (e.g., *HbS*), red cell polymorphisms (e.g., *GYP Dantu*) or enzymopathies (e.g., *G6pD*) play a major role in our defense against this disease (Kariuki and Williams 2020; Kariuki et al. 2020).

These findings support that resistance to diseases (as exemplified here by malaria) is determined by multigenic and/or multiallelic combinations rather than by single allele effects, and that *HLA* class II genes contribute significantly to these complex mechanisms.

## Author Contributions

**Thomas Goeury:** formal analysis (lead), investigation (equal), methodology (equal), software (lead), visualization (equal), writing – original draft (equal). **Ndeye Faye:** investigation (equal), writing – review and editing (equal). **Pascale Gerbault:** investigation (equal), writing – review and editing (equal). **Viktor Černý:** resources (equal), writing – review and editing (equal). **Eric Crubézy:** resources (equal). **Jacques Chiaroni:** resources (equal). **Hacene Brouk:** resources (equal). **Lydie Brunet:** data curation (equal). **Maxime Galan:** software (equal). **Natasja G. de Groot:** investigation (equal), writing – review and editing (equal). **José Manuel Nunes:** investigation (equal), methodology (equal), validation (equal), writing – review and editing (equal). **Alicia Sanchez‐Mazas:** conceptualization (lead), funding acquisition (lead), investigation (lead), project administration (lead), supervision (lead), validation (lead), writing – original draft (lead).

## Conflicts of Interest

The authors declare no conflicts of interest.

## Supporting information


Figures S1–S6.

Tables S1–S10.

## Data Availability

Raw sequence data, individual genotypes and related metadata are available on the public FAIR repository yareta.unige.ch (https://doi.org/10.26037/yareta:3hafz2shyjfoterxpxrbh6htgq) and scripts on gitlab.unige.ch (https://gitlab.unige.ch/asm‐laboratory/DPB1‐malaria).
