# Supplementary material for: Evidence for Pathogen‐Driven Selection Acting on HLA‐DPB1 in Response to Plasmodium falciparum Malaria in West Africa
Source: Ecol Evol. 2025 Feb 24;15(2):e70933. doi: 10.1002/ece3.70933 (PMC11850448; doi:10.1002/ece3.70933)
Supplement: Supplementary file 1 — Figures S1–S6. [file ECE3-15-e70933-s001.pdf]

Supplementary Figures

Evidence for pathogen-driven selection acting on *HLA-DPB1* in response to *Plasmodium falciparum* malaria in West Africa

Thomas Goeury[1], Ndeye Faye[1], Pascale Gerbault[1], Viktor Černý[2], Eric Crubézy[3], Jacques Chiaroni[4], Hacene Brouk[5], Lydie Brunet[1,6,§], Maxime Galan[7], Natasja G. de Groot[8], José Manuel Nunes[1,9] & Alicia Sanchez-Mazas[1,9 *]

[1] Laboratory of Anthropology, Genetics and Peopling history (AGP), Department of Genetics and Evolution, University of Geneva, Geneva, Switzerland

[2]Archaeogenetics Laboratory, Institute of Archaeology of the Academy of Sciences of the Czech Republic, Czech Academy of Sciences, Prague, Czech Republic

[3] Institut universitaire de France, UMR5288 CNRS, University of Toulouse III Paul Sabatier, Toulouse, France

[4]ADES UMR 7268, Aix Marseille University, EFS, CNRS, Marseille, France

[5] Service of Hemobiology and Blood Transfusion, University Hospital Center Ibn Rochd of Annaba, Faculty of Medicine, Badji Mokhtar University of Annaba, Algeria

[6] Transplantation Immunology Unit and National Reference Laboratory for Histocompatibility (UIT/LNRH), Geneva University Hospital, Switzerland

[§] Present address: Thermo Fisher Scientific Inc., e-mail: lydie.brunet@thermofisher.com

[7] CBGP UMR 1062, INRAE, IRD, CIRAD, Montpellier SupAgro, University of Montpellier, Montpellier, France

[8] Department of Comparative Genetics and Refinement, Biomedical Primate Research Centre (BPRC), Rijswijk, The Netherlands

[9] Institute of Genetics and Genomics in Geneva (IGE3), University of Geneva, Geneva, Switzerland

* Corresponding author: Alicia Sanchez-Mazas (ORCID ID: https://orcid.org/0000-0002-7714-2432), Laboratory of Anthropology, Genetics and Peopling history (AGP), Department of Genetics and Evolution, University of Geneva, 30 quai Ernest-Ansermet, 1205 Geneva, Switzerland; e-mail: alicia.sanchez-mazas@unige.cg, phone: +4122 379 6984.

[§] Present address: Thermo Fisher Scientific Inc., e-mail: lydie.brunet@thermofisher.com

Supplementary Figure S1: Stacked frequencies

Distribution of the *HLA* allele frequencies (*HLA* alleles being named with sequence numbers) at loci *DRB1*, *DQA1*, *DQB1* and *DPB1*. Only alleles with a frequency equal or above 10% in at least one population are displayed, «other» grouping the alleles whose frequencies never reach 10%. Short population names correspond to SRR: Senegal-Serer; MAN: Senegal-Mandenka; BED: Senegal-Bedik; SEF: Senegal-Fulani; MAF: Mali-Fulani; MOS: BurkinaFaso-Mossi; GRS: BurkinaFaso-Gurunsi; GUR: BurkinaFaso-Gurmantche; BAG: Chad-BaggaraArabs; DAN: Chad-Dangaleat; DAZ: Chad-Daza; MAB: Chad-Maba; ORO: Ethiopia-Oromo; AMH: Ethiopia-Amhara-(Keketeya); NUB: Sudan-Nubians; SUD: Sudan-SudaneseArabs; RAS: Sudan-RashaaydaArabs; BEJ: Sudan-BejaHadendoa; AMI: Morocco-Amazigh-(Amizmiz); ASN: Morocco-Amazigh-(Asni); FIG: Morocco-Amazigh-(Figuig); ALT: Algeria-(Tamanrasset) and ALC: Algeria-(Constantine). Bands below the population names indicate the geographical region where these populations dwell, W-AFR: West Africa; C-AFR: Central Africa; E-AFR: East Africa and N-AFR: North Africa. See Supplementary Table S3 for the correspondence between sequence names and nominal *HLA* alleles.

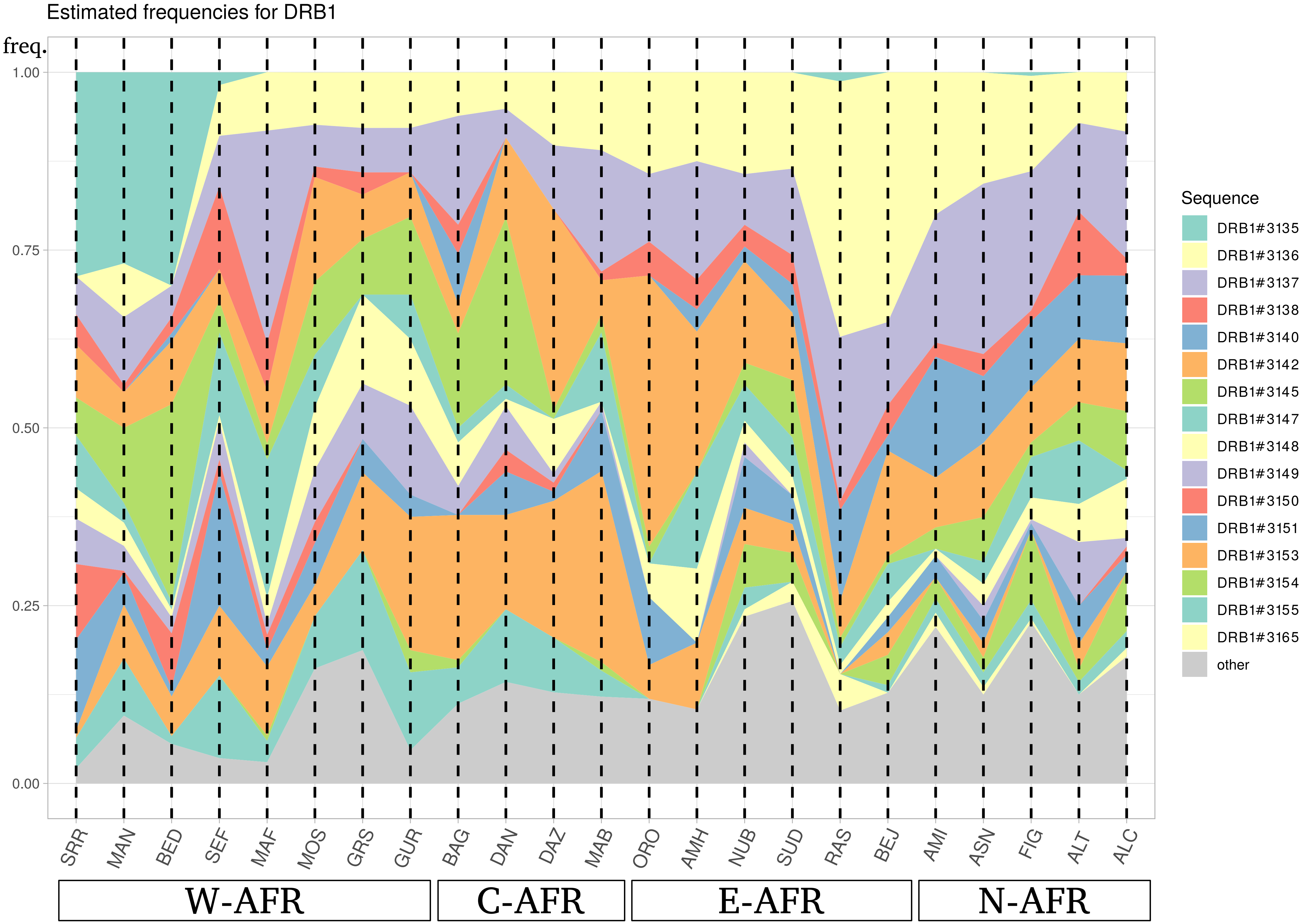

Estimated frequencies for DRB1
freq.
1.00
0.75
0.50
0.25
0.00
SRR
MAN
BED
SEF
MAF
MOS
GRS
GUR
BAG
DAN
DAZ
MAB
ORO
AMH
NUB
SUD
RAS
BEJ
AMI
ASN
FIG
ALT
ALC
W-AFR
C-AFR
E-AFR
N-AFR
Sequence
DRB1#3135
DRB1#3136
DRB1#3137
DRB1#3138
DRB1#3140
DRB1#3142
DRB1#3145
DRB1#3147
DRB1#3148
DRB1#3149
DRB1#3150
DRB1#3151
DRB1#3153
DRB1#3154
DRB1#3155
DRB1#3165
other

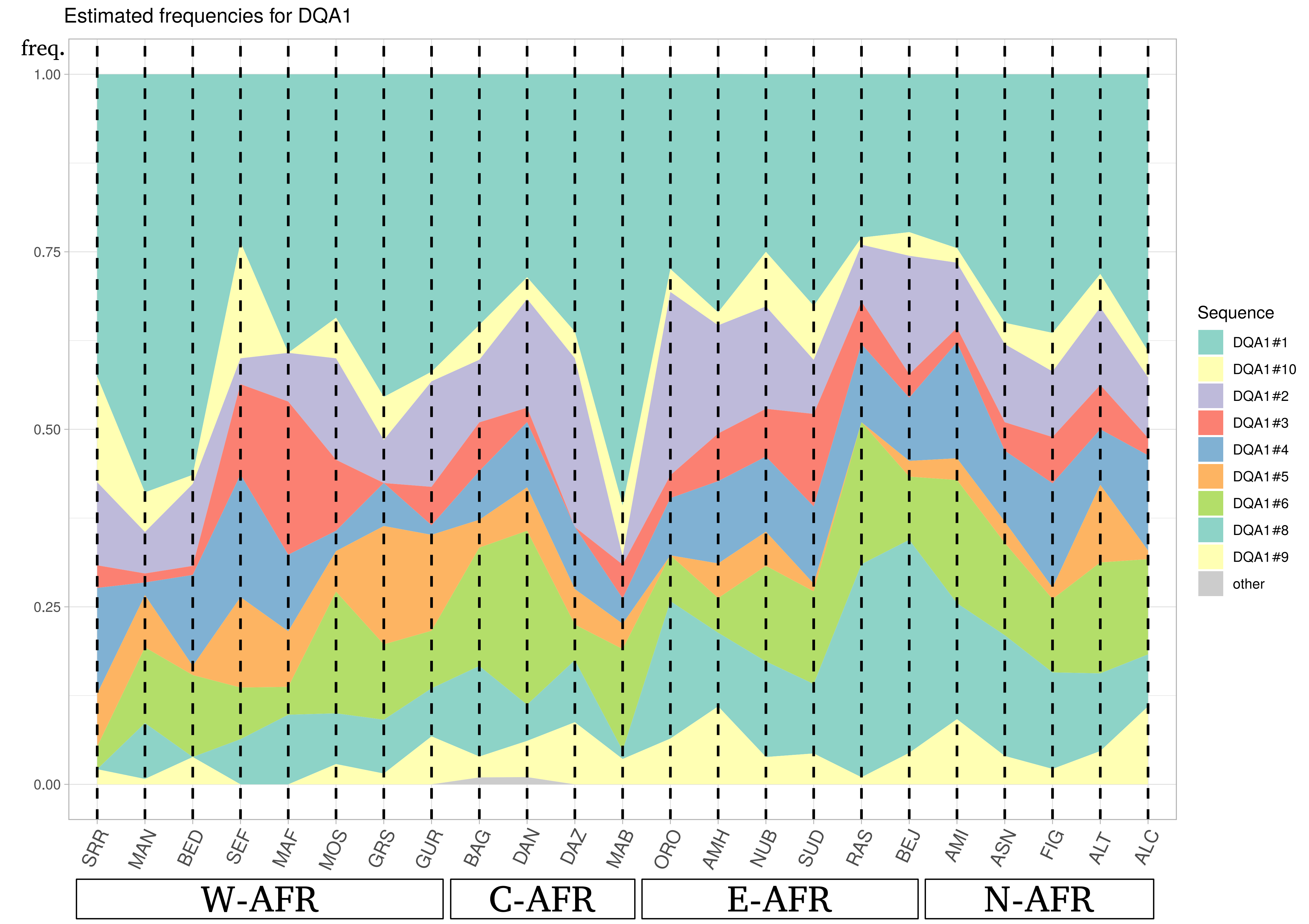
Estimated frequencies for DQA1
freq.
1.00
0.75
0.50
0.25
0.00
SRR
MAN
BED
SEF
MAF
MOS
GRS
GUR
BAG
DAN
DAZ
MAB
ORO
AMH
NUB
SUD
RAS
BEJ
AMI
ASN
FIG
ALT
ALC
W-AFR
C-AFR
E-AFR
N-AFR
Sequence
DQA1#1
DQA1#10
DQA1#2
DQA1#3
DQA1#4
DQA1#5
DQA1#6
DQA1#8
DQA1#9
other

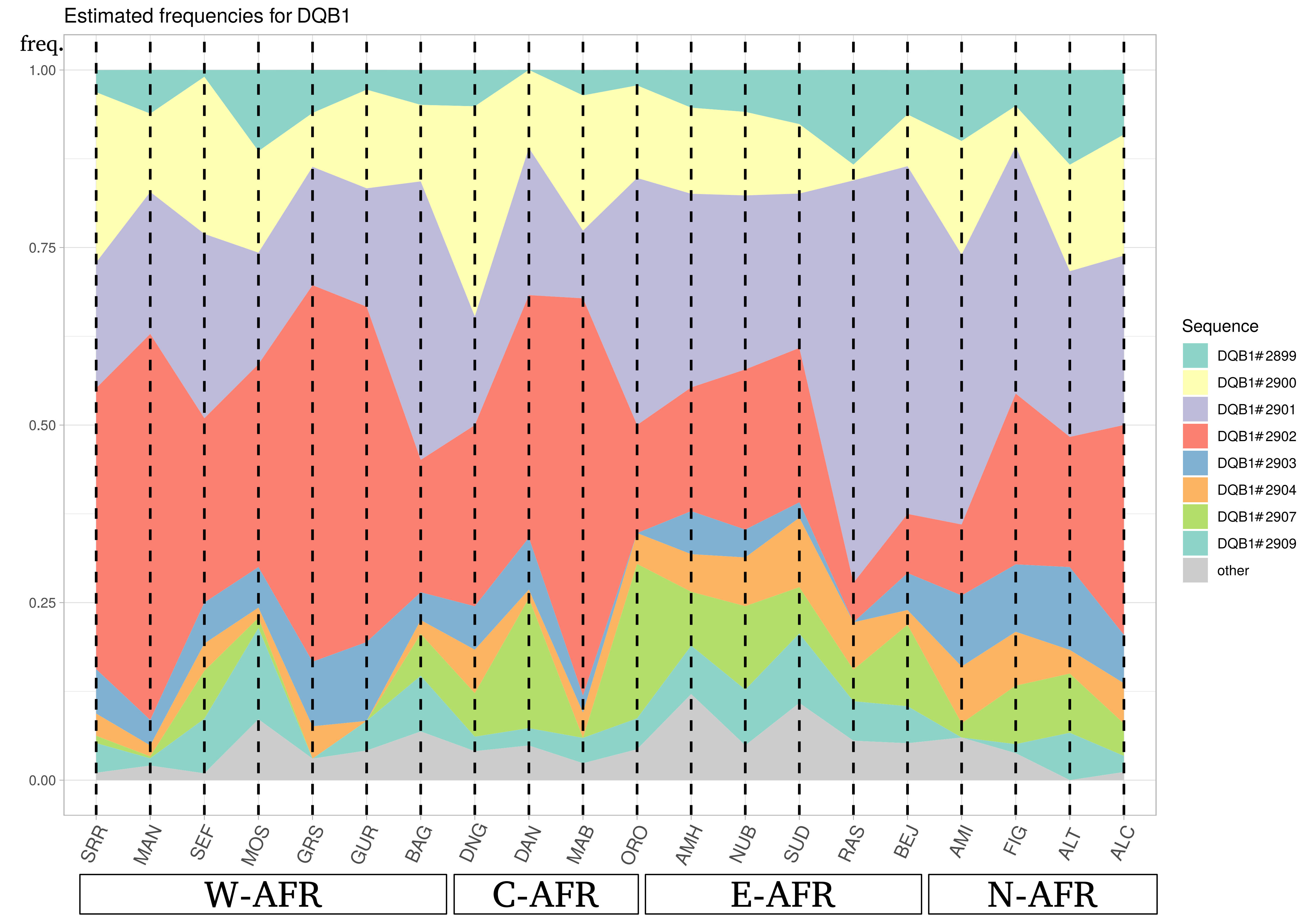
Estimated frequencies for DQB1
freq.
1.00
0.75
0.50
0.25
0.00
SRR
MAN
SEF
MOS
GRS
GUR
BAG
DNG
DAN
MAB
ORO
AMH
NUB
SUD
RAS
BEJ
AMI
FIG
ALT
ALC
W-AFR
C-AFR
E-AFR
N-AFR
Sequence
DQB1#2899
DQB1#2900
DQB1#2901
DQB1#2902
DQB1#2903
DQB1#2904
DQB1#2907
DQB1#2909
other

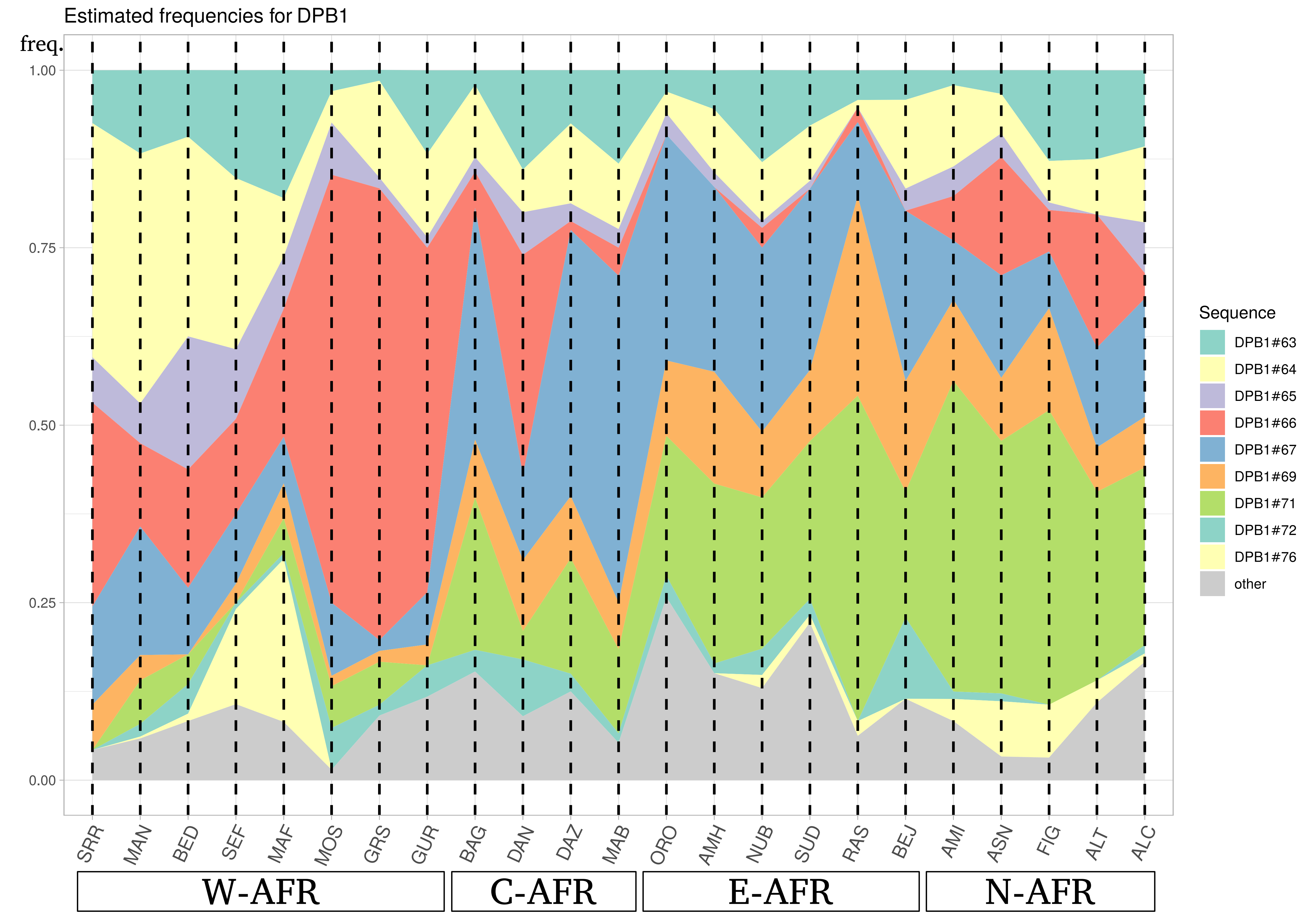

Estimated frequencies for DPB1
freq.
1.00
0.75
0.50
0.25
0.00
SRR
MAN
BED
SEF
MAF
MOS
GRS
GUR
BAG
DAN
DAZ
MAB
ORO
AMH
NUB
SUD
RAS
BEJ
AMI
ASN
FIG
ALT
ALC
W-AFR
C-AFR
E-AFR
N-AFR
Sequence
DPB1#63
DPB1#64
DPB1#65
DPB1#66
DPB1#67
DPB1#69
DPB1#71
DPB1#72
DPB1#76
other

Supplementary Figure S2: Linkage Disequilibrium

A) Results of the non-parametric Global Linkage Disequilibrium (*GLD*) test for the pairs of neighboring *HLA* loci *DRB1~DQA1*, *DQA1~DQB1* and *DQB1~DPB1* in each population tested. Values above 95 (in red) indicate a significant linkage disequilibrium.

B) Upper diagonal: for each pair of *HLA* loci studied, ratio of the number of populations that exhibit significant Global Linkage Disequilibrium (*GLD*, quantile of the non-parametric test of 95 or more) versus the total number of populations tested at these loci (20 per locus). Lower diagonal: for each pair of *HLA* loci studied, ratio (and percentage) of the number of individual haplotypes in significant linkage disequilibrium (*LD*) versus the total number of tested haplotypes (see Supplementary Table S6 for the list of haplotypes in significant *LD* in each population tested).

A)

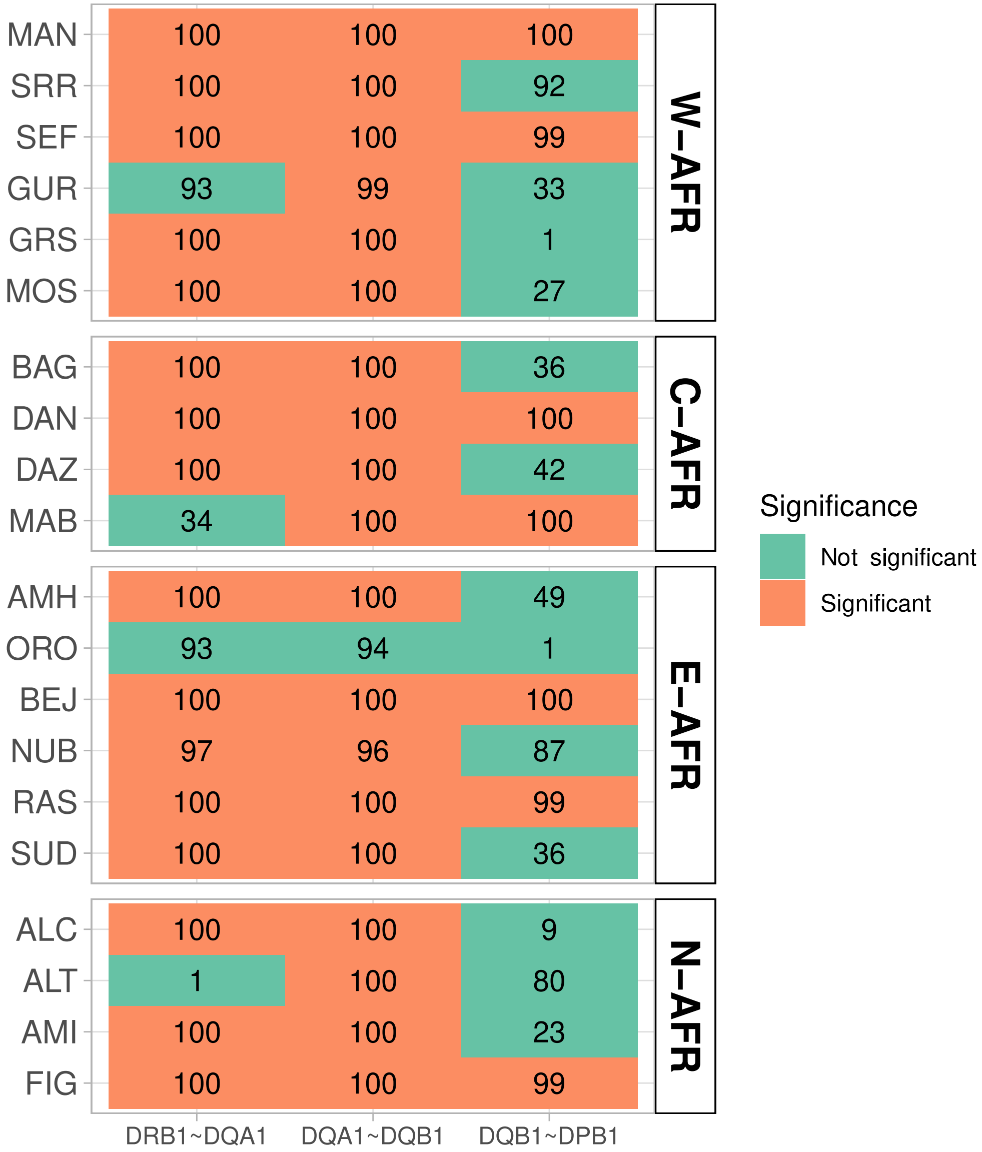

B)

	DRB1	DQA1	DQB1	DPB1
**DRB1**	–	16/20 (80%)	17/20 (85%)	6/20 (30%)
**DQA1**	75/1155 (6%)	–	19/20 (95%)	6/20 (30%)
**DQB1**	76/1111 (7%)	78/793 (10%)	–	7/20 (35%)
**DPB1**	15/1323 (1%)	11/974 (1 %)	10/929 (1%)	–

Supplementary Figure S3: Analysis of Molecular Variance (AMOVA)

Distributions of Φ_{ST} (dots), Φ_{SC} (oblique lines) and Φ_{CT} (crossed lines) fixation indexes at each *HLA* locus studied, where groups of populations are formed according to one of four factors investigated, i.e. geographic region, linguistic family, lifestyle and exposure to *Plasmodium falciparum*. Only populations with sample sizes greater or equal to 20 individuals at all four loci were included. Φ values are indicated on the top of the bars, and « *n.s.* » indicates a Φ non significantly different from zero (α=0.05) after correction for multiple testing (*false discovery rate*). The population groups considered in each category (with corresponding numbers of populations in parentheses) are West Africa (6), Central Africa (4), East Africa (6) and North Africa (4), for « Geographic region »; Niger-Congo (6), Afro-Asiatic (11) and Nilo-Saharan (3), for « Linguistic family »; (semi-)Nomadic (8) and Sedentary (12), for « Lifestyle »; and Strong (*pfpr2000*$\geq$0.05, 11 populations) and Weak (*pfpr2000*<0.05, 9 populations), for « Exposure to *Plasmodium falciparum* ».

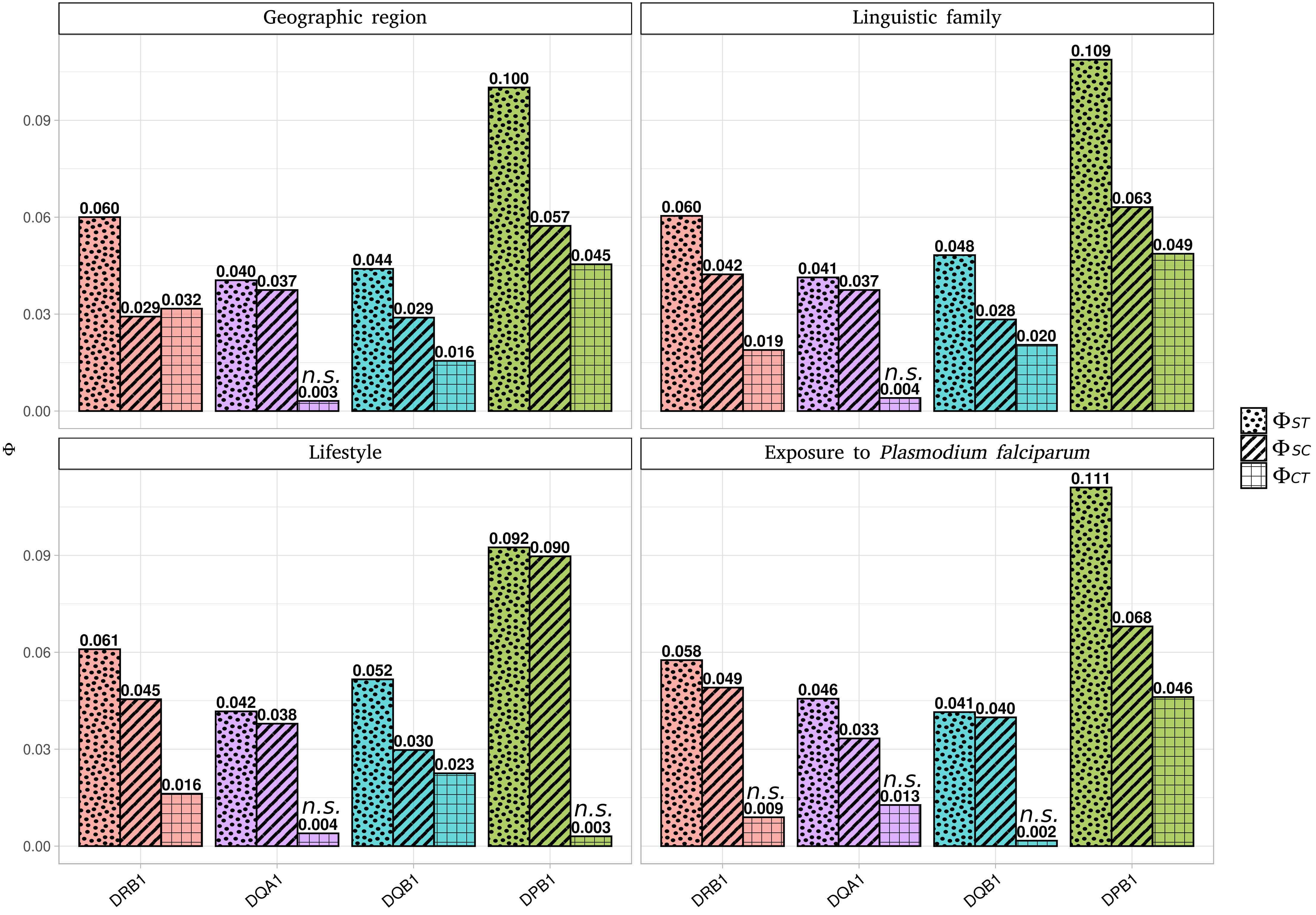

Geographic region
Linguistic family
Lifestyle
Exposure to *Plasmodium falciparum*
Φ
0.00
0.03
0.06
0.09
DRB1
DQA1
DQB1
DPB1
Φ_{ST}
Φ_{SC}
Φ_{CT}
0.060
0.029
0.032
0.040
0.037
n.s.
0.003
0.044
0.029
0.016
0.100
0.057
0.045
0.060
0.042
0.019
0.041
0.037
n.s.
0.004
0.048
0.028
0.020
0.109
0.063
0.049
0.061
0.045
0.016
0.042
0.038
n.s.
0.004
0.052
0.030
0.023
0.092
0.090
n.s.
0.003
0.058
0.049
n.s.
0.009
0.046
0.033
n.s.
0.013
0.041
0.040
n.s.
0.002
0.111
0.068
0.046

Supplementary Figure S4: Heterozygosity heatmap

Heatmap of the estimated heterozygosity (color scale and numerical values) of each population at each locus (empty cells correspond to 3 populations for which sample sizes were below 20 individuals). The « mean±sd » row and column give the average heterozygosity ± one standard deviation at each locus and in each population. W-AFR: West Africa; C-AFR: Central Africa; E-AFR: East Africa and N-AFR: North Africa. Short population names correspond to BED: Senegal-Bedik; MAN: Senegal-Mandenka; SRR: Senegal-Serer; SEF: Senegal-Fulani; MAF: Mali-Fulani; GUR: BurkinaFaso Gurmantche; GRS: BurkinaFaso-Gurunsi; MOS: BurkinaFaso-Mossi; BAG: Chad-BaggaraArabs; DAN: Chad-Dangaleat; DAZ: Chad-Daza; MAB: Chad-Maba; AMH: Ethiopia-Amhara-(Keketeya); ORO: Ethiopia-Oromo; BEJ: Sudan-BejaHadendoa; NUB: Sudan-Nubians; RAS: Sudan-RashaaydaArabs; SUD: Sudan-SudaneseArabs; ALC: Algeria-(Constantine); ALT: Algeria-(Tamanrasset); AMI: Morocco-Amazigh-(Amizmiz); ASN: Morocco-Amazigh-(Asni); FIG: Morocco-Amazigh-(Figuig).

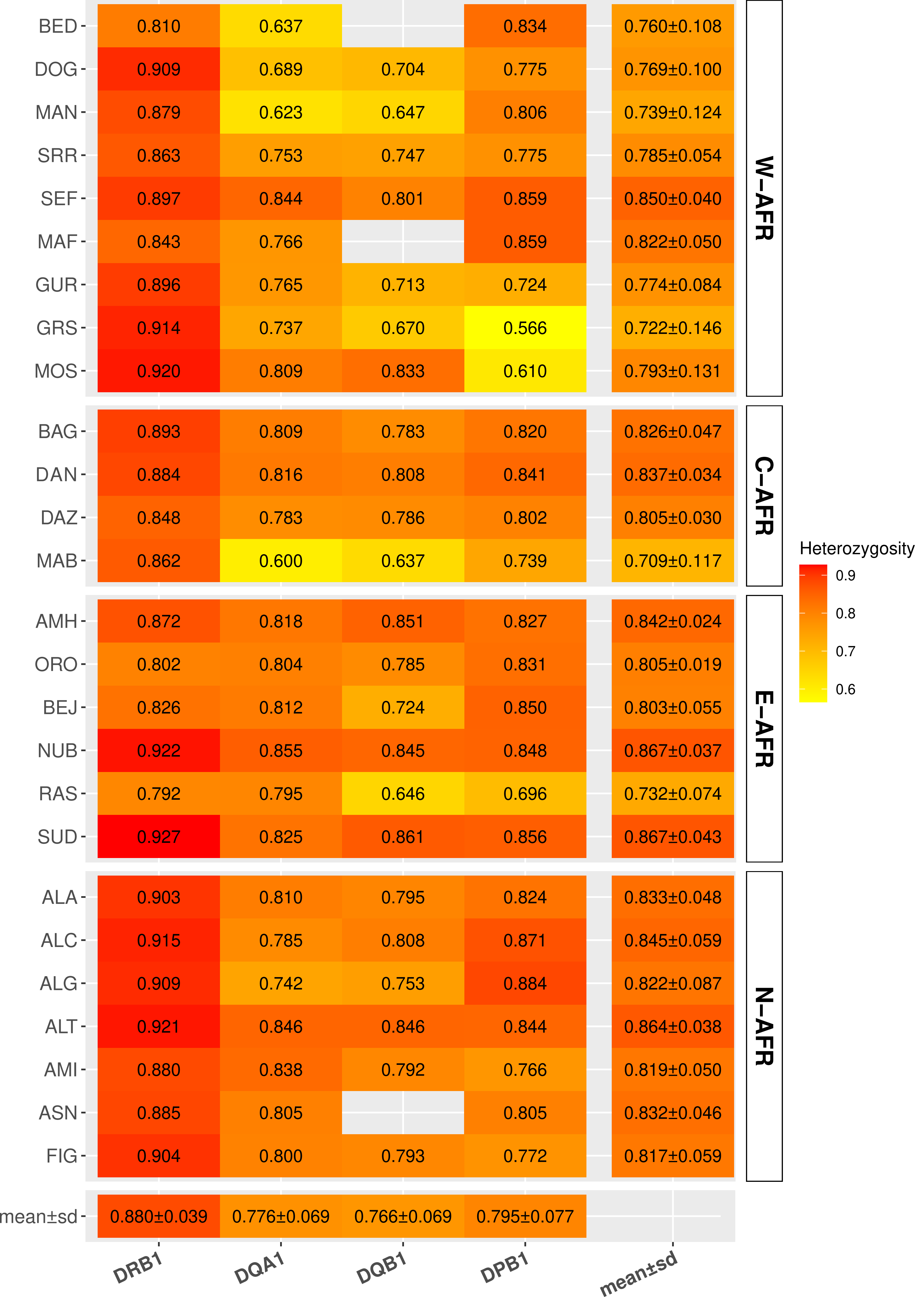

BED 0.810 0.637 0.834 0.760±0.108
DOG 0.909 0.689 0.704 0.775 0.769±0.100
MAN 0.879 0.623 0.647 0.806 0.739±0.124
SRR 0.863 0.753 0.747 0.775 0.785±0.054
SEF 0.897 0.844 0.801 0.859 0.850±0.040
MAF 0.843 0.766 0.859 0.822±0.050
GUR 0.896 0.765 0.713 0.724 0.774±0.084
GRS 0.914 0.737 0.670 0.566 0.722±0.146
MOS 0.920 0.809 0.833 0.610 0.793±0.131
W–AFR
BAG 0.893 0.809 0.783 0.820 0.826±0.047
DAN 0.884 0.816 0.808 0.841 0.837±0.034
DAZ 0.848 0.783 0.786 0.802 0.805±0.030
MAB 0.862 0.600 0.637 0.739 0.709±0.117
C–AFR
AMH 0.872 0.818 0.851 0.827 0.842±0.024
ORO 0.802 0.804 0.785 0.831 0.805±0.019
BEJ 0.826 0.812 0.724 0.850 0.803±0.055
NUB 0.922 0.855 0.845 0.848 0.867±0.037
RAS 0.792 0.795 0.646 0.696 0.732±0.074
SUD 0.927 0.825 0.861 0.856 0.867±0.043
E–AFR
ALA 0.903 0.810 0.795 0.824 0.833±0.048
ALC 0.915 0.785 0.808 0.871 0.845±0.059
ALG 0.909 0.742 0.753 0.884 0.822±0.087
ALT 0.921 0.846 0.846 0.844 0.864±0.038
AMI 0.880 0.838 0.792 0.766 0.819±0.050
ASN 0.885 0.805 0.805 0.832±0.046
FIG 0.904 0.800 0.793 0.772 0.817±0.059
N–AFR
mean±sd 0.880±0.039 0.776±0.069 0.766±0.069 0.795±0.077
DRB1
DQA1
DQB1
DPB1
mean±sd
Heterozygosity
0.9
0.8
0.7
0.6

Supplementary Figure S5: molecular diversity indexes at ARS and non-ARS codons

Violin plots of the average nucleotide diversity (π_n), average number of polymorphic sites (S_n) and Tajima's D distributions computed at ARS (dark colour) and non-ARS (light colour) codons of each *HLA* locus (20 populations considered for all loci). Significance tests (Kruskal-Wallis test, α=0.05, with *fdr* correction (Benjamini & Hochberg, 1995)) have been performed between ARS and non-ARS codons within each locus, as well as between either ARS or non-ARS codons across different loci. Arrows with "*n.s.*" indicate non-significant differences between two distributions (see Supplementary Table S8).

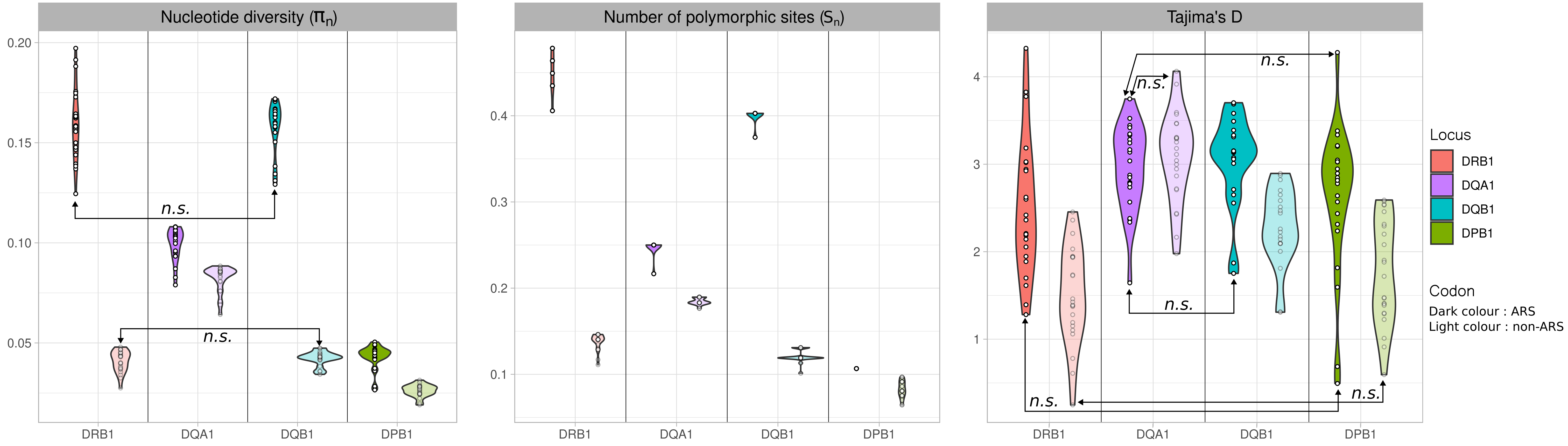

Nucleotide diversity (π_n)
0.20
0.15
0.10
0.05
n.s.
n.s.
DRB1
DQA1
DQB1
DPB1
Number of polymorphic sites (S_n)
0.4
0.3
0.2
0.1
DRB1
DQA1
DQB1
DPB1
Tajima's D
4
3
2
1
n.s.
n.s.
n.s.
n.s.
n.s.
DRB1
DQA1
DQB1
DPB1
Locus
DRB1
DQA1
DQB1
DPB1
Codon
Dark colour : ARS
Light colour : non-ARS

Supplementary Figure S6: *dN*, *dS* and *dN/dS* distributions for humans and chimpanzees

Violin plots of the rate of non-synonymous substitutions (*dN*, left panel), rate of synonymous substitutions (*dS*, center panel) and ratio between the two (*dN/dS*, right panel) for the human populations (white dots, 23 populations for *DRB1*, *DQA1* and *DPB1* and 20 for *DQB1*) and the chimpanzees cohort from the BPRC (white triangles) at each of the four *HLA* (humans) and *Patr* (chimpanzees) loci studied. Colors indicate the locus and black / grey tones the ARS / non-ARS codons of each locus, respectively. « *n.s* » indicates non-significant differences (estimated with 1’000 bootstraps, see Materials & Methods for details) between humans and chimpanzees.

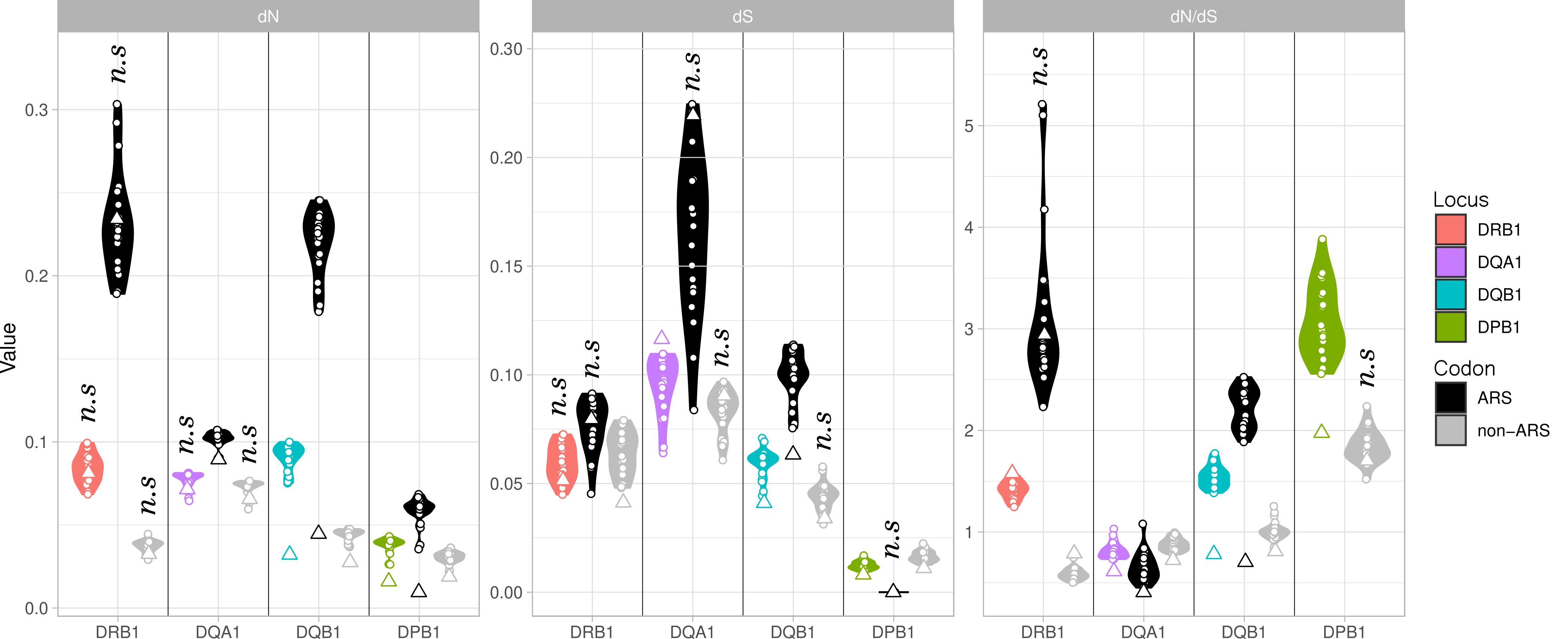
dN
dS
dN/dS
Value
DRB1
DQA1
DQB1
DPB1
n.s
Locus
DRB1
DQA1
DQB1
DPB1
Codon
ARS
non−ARS
