# Supplementary material for: Evidence for Pathogen‐Driven Selection Acting on HLA‐DPB1 in Response to Plasmodium falciparum Malaria in West Africa
Source: Ecol Evol. 2025 Feb 24;15(2):e70933. doi: 10.1002/ece3.70933 (PMC11850448; doi:10.1002/ece3.70933)
Supplement: Supplementary file 2 — Tables S1–S10. [file ECE3-15-e70933-s002.pdf]

Supplementary Tables

Evidence for pathogen-driven selection acting on *HLA-DPB1* in response to *Plasmodium falciparum* malaria in West Africa

Thomas Goeury[1], Ndeye Faye[1], Pascale Gerbault[1], Viktor Černý[2], Eric Crubézy[3], Jacques Chiaroni[4], Hacene Brouk[5], Lydie Brunet[1,6,§], Maxime Galan[7], Natasja G. de Groot[8], José Manuel Nunes[1,9] & Alicia Sanchez-Mazas[1,9 *]

[1] Laboratory of Anthropology, Genetics and Peopling history (AGP), Department of Genetics and Evolution, University of Geneva, Geneva, Switzerland

[2]Archaeogenetics Laboratory, Institute of Archaeology of the Academy of Sciences of the Czech Republic, Czech Academy of Sciences, Prague, Czech Republic

[3] Institut universitaire de France, UMR5288 CNRS, University of Toulouse III Paul Sabatier, Toulouse, France

[4]ADES UMR 7268, Aix Marseille University, EFS, CNRS, Marseille, France

[5] Service of Hemobiology and Blood Transfusion, University Hospital Center Ibn Rochd of Annaba, Faculty of Medicine, Badji Mokhtar University of Annaba, Algeria

[6] Transplantation Immunology Unit and National Reference Laboratory for Histocompatibility (UIT/LNRH), Geneva University Hospital, Switzerland

[§] Present address: Thermo Fisher Scientific Inc., e-mail: lydie.brunet@thermofisher.com

[7] CBGP UMR 1062, INRAE, IRD, CIRAD, Montpellier SupAgro, University of Montpellier, Montpellier, France

[8] Department of Comparative Genetics and Refinement, Biomedical Primate Research Centre (BPRC), Rijswijk, The Netherlands

[9] Institute of Genetics and Genomics in Geneva (IGE3), University of Geneva, Geneva, Switzerland

* Corresponding author: Alicia Sanchez-Mazas (ORCID ID: https://orcid.org/0000-0002-7714-2432), Laboratory of Anthropology, Genetics and Peopling history (AGP), Department of Genetics and Evolution, University of Geneva, 30 quai Ernest-Ansermet, 1205 Geneva, Switzerland; e-mail: alicia.sanchez-mazas@unige.cg, phone: +4122 379 6984.

[§] Present address: Thermo Fisher Scientific Inc., e-mail: lydie.brunet@thermofisher.com

Supplementary Table S1: Summary data of the sampled populations

Collector	Population	Short name	Geographic data		Linguistic data		Lifestyle	Sample sizes (number of individuals) typed at each HLA locus			
			Region	Lat / Long	Language	Family		DRB1	DQA1	DQB1	DPB1
VC	Senegal-Bedik	BED	W-AFR	12.3 / -12.2	Bedik	NC	S	45	39	3*	48
AGP	Senegal-Mandenka	MAN	W-AFR	12.7 / -12.3	Mandenka	NC	S	199	197	195	196
VC	Senegal-Serer	SRR	W-AFR	14.1 / -16.4	Serere	NC	S	47	47	48	47
VC	Senegal-Fulani	SEF	W-AFR	15.3 / -15.1	Fulfulde	NC	N	56	55	52	56
JC	Mali-Dogon	DOG	W-AFR	14.3 / -3.6	Dogon	NC	S	147	150	143	151
JC	Mali-Fulani	MAF	W-AFR	14.3 / -3.1	Fulfulde	NC	N	67	51	18*	61
VC	BurkinaFaso-Gurmantche	GUR	W-AFR	11.2 / 0.7	Gurmantche	NC	S	32	37	36	34
VC	BurkinaFaso-Gurunsi	GRS	W-AFR	11.2 / -1.1	Gurunsi	NC	S	32	33	33	33
VC	BurkinaFaso-Mossi	MOS	W-AFR	12.6 / -1.3	Mossi	NC	S	34	35	35	34
VC	Chad-BaggaraArabs	BAG	C-AFR	13.2 / 18.0	Arab	AA	N	49	51	51	49
VC	Chad-Dangaleat	DAN	C-AFR	12.2 / 18.5	Dangaleat	AA	S	49	49	49	50
VC	Chad-Daza	DAZ	C-AFR	18.2 / 20.6	Daza	NS	SN	39	40	41	40
VC	Chad-Maba	MAB	C-AFR	13.9 / 20.8	Maba	NS	S	41	42	42	38
EC	Ethiopia-Amhara-(Keketeya)	AMH	E-AFR	11.2 / 39.9	Amharic	AA	S	48	82	66	73
EC	Ethiopia-Oromo	ORO	E-AFR	11.3 / 39.6	Oromo	AA	S	21	31	23	33
VC	Sudan-BejaHadendoa	BEJ	E-AFR	15.6 / 36.3	Beja	AA	N	47	45	48	48
VC	Sudan-Nubians	NUB	E-AFR	20.8 / 30.5	Nubian	NS	S	49	52	51	54
VC	Sudan-RashaaydaArabs	RAS	E-AFR	15.3 / 36.2	Arab	AA	SN	39	50	45	48
VC	Sudan-SudaneseArabs	SUD	E-AFR	18.4 / 30.8	Arab	AA	S	37	46	46	45
HB	Algeria-(Annaba)	ALA	N-AFR	36.8 / 7.7	Arab	AA	S	108	113	109	111
HB	Algeria-(Constantine)	ALC	N-AFR	36.2 / 6.6	Arab	AA	S	42	41	44	42
HB	Algeria-(Ghardaia)	ALG	N-AFR	32.4 / 3.6	Tamazight	AA	S	83	80	82	82
HB	Algeria-(Tamanrasset)	ALT	N-AFR	22.7 / 5.5	Tamasheq	AA	N	28	32	30	32
JMD	Morocco-Amazigh-(Amizmiz)	AMI	N-AFR	31.2 / -8.2	Tamazight	AA	SN	50	49	25	48
JMD	Morocco-Amazigh-(Asni)	ASN	N-AFR	31.2 / -8.0	Tamazight	AA	SN	48	50	0*	45
JMD	Morocco-Amazigh-(Figuig)	FIG	N-AFR	32.1 / -1.2	Tamazight	AA	SN	97	92	79	94

Collectors are Hacene Brouk (HB, University Badji Mokhtar, Annaba, Algeria), Jean-Michel Dugoujon (JMD, University Paul Sabatier Toulouse III, Toulouse, France), Viktor Černý (VC, Archeology Institute, Prague, Czech Republic), Jacques Chiaroni (JC, Établissement français du sang, Marseille, France), Eric Crubézy (EC, University Paul Sabatier Toulouse III, Toulouse, France) and the Laboratory of Anthropology, Genetics and Peopling history at Geneva (AGP). Population names are composed of the country followed either by the precise population name or, in parentheses, by the geographical location (town or village). Short names correspond to three letter codes for the populations. Column « Region » provides geographic regions as defined by the UN geoscheme and named according to (Nunes et al., 2014): W-AFR: West Africa; C-AFR: Central Africa; E-AFR: East Africa and N-AFR: North Africa. Populations from Sudan, although being geographically in North Africa, have been labeled as East African due to their closer geographic proximity with other East African peoples than North African. « Coordinates » correspond to the latitude and longitude of sampling locations (decimal units). « Lifestyle » correspond to sedentary (S), semi-nomad (SN) and nomad (N). Parentheses after the languages indicate the linguistic families: Afro-Asiatic (AA), Nilo-Saharan (NS) and Niger-Congo (NC). The four columns « Sampling sizes » give the numbers of available samples for the analyses (excluding ambiguous genotypes and replicates) at each locus. Loci tested for less than 20 individuals are indicated by a « * » and have been excluded from the analyses.

Supplementary Table S2: MADaM processing

	Locus	DRB1 (Exon2)	DQA1 (Exon2)	DQB1 (Exon2)	DPB1 (Exon2)
Reads	Total reads	1605818	1813556	1664894	1536091
	Rejected reads	712393	238750	628441	489565
	(markovian filter)	183221	0	130419	0
	(other filters)	529172	238750	498022	489565
	Assigned reads	893425	1574806	1036453	1046526
Genotypings	Nb. variants	318065	551581	336172	383339
	Nb. alleles	75	61	27	52
	Nb. samples	3010	3186	2769	2453
Reproducibility	Replicates	255/267	256/281	347/386	263/267
	Accuracy	95.5 %	91.1 %	83.9 %	98.5%
Source of error	Pre-computing	4	2	32	2
	MADaM	5	20	6	2
	Unknown	3	3	1	0

For each of the four loci, this table provides: « total reads »: the total number of reads available (for the three runs) after sequencing and filtering based on the PhredScore; « Rejected reads »: the number of reads rejected by MADaM, either by the markovian filter (row « (markovian filter) ») for DRB1 and DQB1 or by other (row « (other filters) ») filters such as size limit, BLAST or primers/tags incorrect match; « Assigned reads »: number of reads that have been successfully assigned to a sample; « Nb. variants »: number of assigned reads that are unique within a sample; « Nb. alleles »: number of unique reads that was predicted as true variants; « Nb. samples »: number of sample for which a genotype was obtained; « Replicates »: ratio of the number of matching pairs of replicates (identical genotypes) and the total number pairs of genotypes (for which a genotype is obtained for both samples of the pair); « Accuracy »: percentage of matching pairs of replicates among all usable pairs of replicates; The three rows « Source of error » give the potential origin of discrepancies between replicates: « Pre-computing » corresponds to errors due to (mostly) library preparation and sequencing, « MADaM » corresponds to errors during the reads processing by MADaM and « Unknown » to errors which can not be attributed to one of the previous sources.

Supplementary Table S3: List of HLA nominal alleles associated with each HLA Exon 2 sequence identified in this study. The following tables list all possible IMGT/HLA named alleles at the first, second and third field levels of resolution (database version 3.35.0, Robinson et al., 2015) associated to each HLA Exon 2 sequence identified in this study.

HLA-DRB1 Exon 2 sequence number	HLA-DRB1 Nominal Alleles		
	1st field	2nd field	3rd field
#3135	DRB1*13	DRB1*13:04	
#3136	DRB1*07	DRB1*07:01	DRB1*07:01:01
			DRB1*07:01:18
			DRB1*07:01:21
		DRB1*07:100	
		DRB1*07:33	
		DRB1*07:34	
		DRB1*07:56	
		DRB1*07:72	
		DRB1*07:79	
		DRB1*07:81	
		DRB1*07:85	
		DRB1*07:87N	
		DRB1*07:90	
		DRB1*07:92	
		DRB1*07:93	
		DRB1*07:95	
		DRB1*07:96	
#3137	DRB1*03	DRB1*03:01	DRB1*03:01:01
			DRB1*03:01:08
			DRB1*03:01:11
			DRB1*03:01:26
			DRB1*03:01:27
			DRB1*03:01:28
		DRB1*03:100:02	
		DRB1*03:104	
		DRB1*03:123	
		DRB1*03:124	
		DRB1*03:127	
		DRB1*03:132	

		DRB1*03:137	
		DRB1*03:144	
		DRB1*03:146	
		DRB1*03:147	
		DRB1*03:151	
		DRB1*03:152	
		DRB1*03:50	
		DRB1*03:68N	
		DRB1*03:83	
#3138	DRB1*04	DRB1*04:05	DRB1*04:05:01
			DRB1*04:05:03
			DRB1*04:05:04
			DRB1*04:05:19
			DRB1*04:05:20
		DRB1*04:213	
		DRB1*04:232	
		DRB1*04:245	
#3139	DRB1*14	DRB1*14:01	DRB1*14:01:01
			DRB1*14:01:04
		DRB1*14:113	
		DRB1*14:114	
		DRB1*14:125	
		DRB1*14:142	
		DRB1*14:157	
		DRB1*14:192	
		DRB1*14:202	
		DRB1*14:204	
		DRB1*14:207	
		DRB1*14:54	DRB1*14:54:01
			DRB1*14:54:06
			DRB1*14:54:07
		DRB1*15:01	DRB1*15:01:01
			DRB1*15:01:17
			DRB1*15:01:32
			DRB1*15:01:34
			DRB1*15:01:35
			DRB1*15:01:36

#3140	DRB1*15		DRB1*15:01:37
			DRB1*15:01:38
		DRB1*15:110	
		DRB1*15:123	
		DRB1*15:124	
		DRB1*15:132	
		DRB1*15:141	
		DRB1*15:145	
		DRB1*15:146	
		DRB1*15:150	
		DRB1*15:151	
		DRB1*15:160	
		DRB1*15:74	
		DRB1*15:85	
		DRB1*15:86	
#3142	DRB1*13	DRB1*13:02	DRB1*13:02:01
			DRB1*13:02:08
			DRB1*13:02:14
			DRB1*13:02:15
			DRB1*13:02:16
			DRB1*13:02:17
		DRB1*13:109	
		DRB1*13:128	
		DRB1*13:208	
		DRB1*13:212	
		DRB1*13:236	
		DRB1*13:239	
		DRB1*13:255N	
		DRB1*13:265	
		DRB1*13:268N	
		DRB1*13:269	
		DRB1*13:275	
#3143	DRB1*16	DRB1*16:02:01	
		DRB1*16:22	
		DRB1*16:35	
		DRB1*16:52	
#3144	DRB1*08	DRB1*08:06	

#3145	DRB1*11	DRB1*11:01	DRB1*11:01:01
			DRB1*11:01:02
			DRB1*11:01:06
			DRB1*11:01:08
			DRB1*11:01:21
			DRB1*11:01:29
			DRB1*11:01:30
			DRB1*11:01:31
			DRB1*11:01:33
		DRB1*11:100	
		DRB1*11:208	
		DRB1*11:234	
		DRB1*11:236	
		DRB1*11:238	
		DRB1*11:239	
		DRB1*11:240	
		DRB1*11:243	
		DRB1*11:250N	
		DRB1*11:252	
		DRB1*11:254	
		DRB1*11:97	
#3147	DRB1*13	DRB1*13:01	DRB1*13:01:01
			DRB1*13:01:08
			DRB1*13:01:17
			DRB1*13:01:18
			DRB1*13:01:19
			DRB1*13:01:20
			DRB1*13:01:21
			DRB1*13:01:24
			DRB1*13:01:25
			DRB1*13:01:26
		DRB1*13:105	
		DRB1*13:112	
		DRB1*13:117	
		DRB1*13:166	
		DRB1*13:186	
		DRB1*13:190	

		DRB1*13:215	
		DRB1*13:218	
		DRB1*13:233	
		DRB1*13:238	
		DRB1*13:251	
		DRB1*13:252N	
		DRB1*13:256	
		DRB1*13:261	
		DRB1*13:263	
		DRB1*13:267	
		DRB1*13:270	
		DRB1*13:271	
		DRB1*13:272	
		DRB1*13:273	
		DRB1*13:274	
**#3148**	**DRB1*13**	DRB1*13:03	DRB1*13:03:01
			DRB1*13:03:07
			DRB1*13:03:08
			DRB1*13:03:09
		DRB1*13:227	
		DRB1*13:253	
**#3149**	**DRB1*03**	DRB1*03:02	DRB1*03:02:01
			DRB1*03:02:03
		DRB1*03:154	
**#3150**	**DRB1*09**	DRB1*09:01:02:01	
		DRB1*09:09	
		DRB1*09:21	
		DRB1*09:25	
		DRB1*09:31	
		DRB1*09:33	
		DRB1*09:35	
		DRB1*09:39	
**#3151**	**DRB1*10**	DRB1*10:01	DRB1*10:01:01
			DRB1*10:01:04
			DRB1*10:01:10
			DRB1*10:01:12
		DRB1*10:19	

		DRB1*10:25	
		DRB1*10:28	
		DRB1*10:31	
		DRB1*10:33	
**#3152**	**DRB1*15**	DRB1*15:03	DRB1*15:03:01
			DRB1*15:03:03
**#3153**	**DRB1*08**	DRB1*08:04	DRB1*08:04:01
			DRB1*08:04:04
		DRB1*08:59	
**#3154**	**DRB1*11**	DRB1*11:04	DRB1*11:04:01
			DRB1*11:04:02
			DRB1*11:04:06
			DRB1*11:04:09
			DRB1*11:04:15
			DRB1*11:04:16
			DRB1*11:04:17
		DRB1*11:187	
		DRB1*11:198	
		DRB1*11:210	
		DRB1*11:211	
		DRB1*11:220	
		DRB1*11:224	
		DRB1*11:230	
		DRB1*11:244	
		DRB1*11:253	
**#3155**	DRB1*11	DRB1*11:02:01	
		DRB1*11:242	
		DRB1*11:251	
**#3156**	**DRB1*12**	DRB1*12:01:01	
		DRB1*12:06	
		DRB1*12:10	
		DRB1*12:17	
		DRB1*12:68	
		DRB1*12:70	
		DRB1*12:72N	
**#3157**	**DRB1*14**	DRB1*14:06	DRB1*14:06:01
			DRB1*14:06:02

		DRB1*14:176	
#3158	DRB1*08	DRB1*08:01	DRB1*08:01:01
			DRB1*08:01:05
		DRB1*08:39	
		DRB1*08:55	
		DRB1*08:64	
		DRB1*08:77	
		DRB1*08:86	
		DRB1*08:88	
#3159	DRB1*15	DRB1*15:02	DRB1*15:02:01
			DRB1*15:02:09
		DRB1*15:105:02	
		DRB1*15:126	
		DRB1*15:140	
		DRB1*15:149	
		DRB1*15:154N	
		DRB1*15:159N	
		DRB1*15:161	
		DRB1*15:19	
#3160	DRB1*04	DRB1*04:02	DRB1*04:02:01
			DRB1*04:02:06
#3161	DRB1*04	DRB1*04:03	DRB1*04:03:01
			DRB1*04:03:03
			DRB1*04:03:13
			DRB1*04:03:15
		DRB1*04:181	
		DRB1*04:195	
		DRB1*04:218	
		DRB1*04:52	
#3163	DRB1*04	DRB1*04:06	DRB1*04:06:01
			DRB1*04:06:02
			DRB1*04:06:07
		DRB1*04:01	DRB1*04:01:01
			DRB1*04:01:08
		DRB1*04:151	
		DRB1*04:192	
		DRB1*04:242	

**#3164**	**DRB1*04**	DRB1*04:243	
		DRB1*04:248	
		DRB1*04:254	
		DRB1*04:257	
		DRB1*04:266N	
		DRB1*04:268	
		DRB1*04:270	
**#3165**	**DRB1*16**	DRB1*16:01:01	DRB1*16:01:01
			DRB1*16:01:08
			DRB1*16:01:10
			DRB1*16:01:15
		DRB1*16:49	
		DRB1*16:51	
**#3167**	**DRB1*07**	DRB1*07:24	
**#3168**	**DRB1*08**	DRB1*08:02	DRB1*08:02:01
			DRB1*08:02:02
			DRB1*08:04:01
			DRB1*08:04:03
			DRB1*08:04:04
		DRB1*08:59	
		DRB1*08:84	
**#3169**	**DRB1*04**	DRB1*04:04	DRB1*04:04:01
			DRB1*04:04:14
			DRB1*04:04:15
		DRB1*04:23	
		DRB1*04:258	
		DRB1*04:272	
**#3170**	**DRB1*11**	DRB1*11:03	DRB1*11:03:01
			DRB1*11:03:02
			DRB1*11:03:03
			DRB1*11:03:04
		DRB1*11:231	
**#3172**	**DRB1*14**	DRB1*14:04	DRB1*14:04:01
			DRB1*14:04:04
		DRB1*14:196	
**#3173**	**DRB1*08**	DRB1*08:02	DRB1*08:02:01
			DRB1*08:02:02

		DRB1*08:84
**#3183**	**DRB1*04**	DRB1*04:08:01
		DRB1*04:252

**HLA-DQA1 Exon 2 sequence number**	**HLA-DQA1 Nominal Alleles**		
	**1st field**	**2nd field**	**3rd field**
**#1**	**DQA1*05**	DQA1*05:01	DQA1*05:01:01
			DQA1*05:01:04
		DQA1*05:03:01	
		DQA1*05:05:01	
		DQA1*05:06:01	
		DQA1*05:07	
		DQA1*05:08	
		DQA1*05:09	
		DQA1*05:11	
		DQA1*05:13	
		DQA1*05:14	
**#2**	**DQA1*01**	DQA1*01:02	DQA1*01:02:01
			DQA1*01:02:02
			DQA1*01:02:03
			DQA1*01:02:04
		DQA1*01:11	
		DQA1*01:19	
		DQA1*01:21	
**#3**	**DQA1*01**	DQA1*01:03:01	
		DQA1*01:10	
**#4**	**DQA1*03**	DQA1*03:01:01	
		DQA1*03:02:01	
		DQA1*03:03	DQA1*03:03:01
			DQA1*03:03:02
		DQA1*03:04	DQA1*03:04
		DQA1*04:01	DQA1*04:01:01

**#5**	**DQA1*04**	DQA1*04:01	DQA1*04:01:02
		DQA1*04:02	
**#6**	**DQA1*01**	DQA1*01:02	DQA1*01:02:01
			DQA1*01:02:02
			DQA1*01:02:03
			DQA1*01:02:04
		DQA1*01:11	
		DQA1*01:19	
		DQA1*01:21	
**#8**	**DQA1*02**	DQA1*02:01:01	
		DQA1*02:02N	
		DQA1*02:03	
**#9**	**DQA1*01**	DQA1*01:01:01	
		DQA1*01:01:02	
		DQA1*01:04	DQA1*01:04:01
			DQA1*01:04:02
		DQA1*01:05	DQA1*01:05:01
			DQA1*01:05:02
		DQA1*01:07Q	
**#10**	**DQA1*01**	DQA1*01:01	DQA1*01:01:01
			DQA1*01:01:02
		DQA1*01:04	DQA1*01:04:01
			DQA1*01:04:02
		DQA1*01:05	DQA1*01:05:01
			DQA1*01:05:02
		DQA1*01:07Q	
**#12**	**DQA1*01**	DQA1*01:02	DQA1*01:02:01
			DQA1*01:02:02
			DQA1*01:02:03
			DQA1*01:02:04
		DQA1*01:11	

		DQA1*01:19	
		DQA1*01:21	

HLA-DQB1 Exon 2 sequence number	HLA-DQB1 Nominal Alleles		
	1st field	2nd field	3rd field
#2899	DQB1*06	DQB1*06:02	DQB1*06:02:01
			DQB1*06:02:04
			DQB1*06:02:12
			DQB1*06:02:23
			DQB1*06:02:26
			DQB1*06:02:27
			DQB1*06:02:28
			DQB1*06:02:29
			DQB1*06:02:32
			DQB1*06:02:34
			DQB1*06:02:36
			DQB1*06:02:37
			DQB1*06:02:38
		DQB1*06:109	
		DQB1*06:11:01	
		DQB1*06:111	
		DQB1*06:112N	
		DQB1*06:115	
		DQB1*06:116	
		DQB1*06:117	
		DQB1*06:127	
		DQB1*06:131	
		DQB1*06:175	
		DQB1*06:176	
		DQB1*06:188	
		DQB1*06:200	
		DQB1*06:216N	
		DQB1*06:219	

		DQB1*06:224	
		DQB1*06:225	
		DQB1*06:226	
		DQB1*06:227	
		DQB1*06:228	
		DQB1*06:237	
		DQB1*06:240	
		DQB1*06:255	
		DQB1*06:256	
		DQB1*06:262	
		DQB1*06:270	
		DQB1*06:273	
		DQB1*06:284	
		DQB1*06:286	
		DQB1*06:289	
		DQB1*06:293	
		DQB1*06:295	
		DQB1*06:296	
		DQB1*06:297	
		DQB1*06:298	
		DQB1*06:300	
		DQB1*06:47	
		DQB1*06:72	
		DQB1*06:84	
		DQB1*05:01	
		DQB1*05:01	DQB1*05:01:21
			DQB1*05:01:22
			DQB1*05:01:23
			DQB1*05:01:24
			DQB1*05:01:26
			DQB1*05:01:27
			DQB1*05:01:28
			DQB1*05:01:29

#2900	DQB1*05	DQB1*05:103
		DQB1*05:104
		DQB1*05:107
		DQB1*05:120
		DQB1*05:128N
		DQB1*05:133
		DQB1*05:137
		DQB1*05:139
		DQB1*05:144
		DQB1*05:148
		DQB1*05:150
		DQB1*05:152
		DQB1*05:155
		DQB1*05:156
		DQB1*05:159
		DQB1*05:160
		DQB1*05:162
		DQB1*05:163
		DQB1*05:164
		DQB1*05:173
		DQB1*05:18
		DQB1*05:182
		DQB1*05:183
		DQB1*05:184
		DQB1*05:185N
		DQB1*05:190
		DQB1*05:193
		DQB1*05:194
		DQB1*05:195
		DQB1*05:197
		DQB1*05:27
		DQB1*05:31
		DQB1*05:32

		DQB1*05:45	
		DQB1*05:62	
		DQB1*05:74	
		DQB1*05:84	
		DQB1*02:01	DQB1*02:01:01
			DQB1*02:01:08
			DQB1*02:01:10
			DQB1*02:01:26
			DQB1*02:01:27
		DQB1*02:02	DQB1*02:02:01
			DQB1*02:02:02
			DQB1*02:02:03
			DQB1*02:02:05
			DQB1*02:02:06
			DQB1*02:02:07
			DQB1*02:02:08
			DQB1*02:02:09
		DQB1*02:04	
		DQB1*02:06	
		DQB1*02:09	
		DQB1*02:10	
		DQB1*02:102	
		DQB1*02:104	
		DQB1*02:105	
		DQB1*02:106	
		DQB1*02:107	
		DQB1*02:108	
		DQB1*02:109	
		DQB1*02:110	
		DQB1*02:111	
		DQB1*02:112	
		DQB1*02:113	
		DQB1*02:114	

#2901	DQB1*02	DQB1*02:118
		DQB1*02:119
		DQB1*02:12
		DQB1*02:120
		DQB1*02:121
		DQB1*02:122
		DQB1*02:125
		DQB1*02:126
		DQB1*02:127
		DQB1*02:128
		DQB1*02:131
		DQB1*02:133
		DQB1*02:136
		DQB1*02:137
		DQB1*02:138
		DQB1*02:139
		DQB1*02:20N
		DQB1*02:29
		DQB1*02:34
		DQB1*02:48
		DQB1*02:59
		DQB1*02:61
		DQB1*02:64
		DQB1*02:65
		DQB1*02:75
		DQB1*02:79
		DQB1*02:80
		DQB1*02:81
		DQB1*02:82
		DQB1*02:89:01
		DQB1*02:89:02
		DQB1*02:96N
		DQB1*02:97

		DQB1*02:98	
		DQB1*02:99	
		DQB1*03:01	DQB1*03:01:01
			DQB1*03:01:04
			DQB1*03:01:05
			DQB1*03:01:09
			DQB1*03:01:10
			DQB1*03:01:11
			DQB1*03:01:12
			DQB1*03:01:20
			DQB1*03:01:26
			DQB1*03:01:31
			DQB1*03:01:32
			DQB1*03:01:35
			DQB1*03:01:36
			DQB1*03:01:39
			DQB1*03:01:40
			DQB1*03:01:41
			DQB1*03:01:43
			DQB1*03:01:44
			DQB1*03:01:46
		DQB1*03:09	
		DQB1*03:115	
		DQB1*03:116	
		DQB1*03:120	
		DQB1*03:127	
		DQB1*03:150	
		DQB1*03:157	
		DQB1*03:158	
		DQB1*03:164	
		DQB1*03:165	
		DQB1*03:169	
		DQB1*03:182	

#2902	DQB1*03	DQB1*03:19	DQB1*03:19:01
			DQB1*03:19:03
			DQB1*03:19:04
		DQB1*03:191	
		DQB1*03:196	
		DQB1*03:198	DQB1*03:198:01
			DQB1*03:198:02
		DQB1*03:206	
		DQB1*03:21	
		DQB1*03:22	
		DQB1*03:236	
		DQB1*03:24	
		DQB1*03:241	
		DQB1*03:243	
		DQB1*03:246	
		DQB1*03:253	
		DQB1*03:256	
		DQB1*03:264	
		DQB1*03:266	
		DQB1*03:276N	
		DQB1*03:281	
		DQB1*03:284	
		DQB1*03:285	
		DQB1*03:286	
		DQB1*03:288	
		DQB1*03:29	
		DQB1*03:290	
		DQB1*03:292	
		DQB1*03:293	
		DQB1*03:294	
		DQB1*03:297	
		DQB1*03:302	
		DQB1*03:309	

		DQB1*03:312	
		DQB1*03:317:0[illegible]	DQB1*03:317:01
			DQB1*03:317:02
		DQB1*03:326	
		DQB1*03:328	
		DQB1*03:329	
		DQB1*03:330	
		DQB1*03:331	
		DQB1*03:338N	
		DQB1*03:340N	
		DQB1*03:342	
		DQB1*03:347	
		DQB1*03:35	
		DQB1*03:350	
		DQB1*03:42	
		DQB1*03:49	
		DQB1*03:50	
		DQB1*03:51	
		DQB1*03:52	
		DQB1*03:84N	
		DQB1*03:94	
		DQB1*04:02	DQB1*04:02:01
			DQB1*04:02:10
			DQB1*04:02:12
			DQB1*04:02:13
			DQB1*04:02:14
			DQB1*04:02:15
			DQB1*04:02:18
		DQB1*04:04	
		DQB1*04:13	
		DQB1*04:23	
		DQB1*04:36N	
		DQB1*04:39	

#2903	DQB1*04	DQB1*04:41N	
		DQB1*04:43	
		DQB1*04:44	
		DQB1*04:45	
		DQB1*04:46N	
		DQB1*04:51	
		DQB1*04:52	
		DQB1*04:54	
		DQB1*04:55	
		DQB1*04:56	
		DQB1*04:57	
		DQB1*04:59N	
		DQB1*04:60	
#2904	DQB1*03	DQB1*03:02	DQB1*03:02:01
			DQB1*03:02:09
			DQB1*03:02:12
			DQB1*03:02:17
			DQB1*03:02:21
			DQB1*03:02:24
			DQB1*03:02:25
			DQB1*03:02:26
			DQB1*03:02:27
			DQB1*03:02:29
		DQB1*03:106	
		DQB1*03:190	
		DQB1*03:215	
		DQB1*03:245	
		DQB1*03:247	
		DQB1*03:251	
		DQB1*03:263	
		DQB1*03:265	
		DQB1*03:273	
		DQB1*03:287	

		DQB1*03:289	
		DQB1*03:295	
		DQB1*03:296	
		DQB1*03:298	
		DQB1*03:310N	
		DQB1*03:315	
		DQB1*03:32	
		DQB1*03:321	
		DQB1*03:324	
		DQB1*03:334N	
		DQB1*03:343	
		DQB1*03:345	
		DQB1*03:348	
		DQB1*03:349	
		DQB1*03:85	
**#2905**	**DQB1*03**	DQB1*03:03	DQB1*03:03:02
			DQB1*03:03:04
			DQB1*03:03:09
			DQB1*03:03:10
			DQB1*03:03:14
			DQB1*03:03:20
		DQB1*03:117	
		DQB1*03:222	
		DQB1*03:248	
		DQB1*03:249	
		DQB1*03:270	
		DQB1*03:30	
		DQB1*03:31	
		DQB1*03:316	
		DQB1*03:319	
		DQB1*03:33	
		DQB1*03:332	
		DQB1*03:39	

		DQB1*03:43	
		DQB1*03:79	
		DQB1*03:87	
		DQB1*03:88	
		DQB1*03:89	
		DQB1*03:91Q	
		DQB1*03:96	
		DQB1*03:97	
		DQB1*03:98	
#2906	DQB1*06	DQB1*06:09	DQB1*06:09:01
			DQB1*06:09:09
			DQB1*06:09:10
		DQB1*06:189	
		DQB1*06:22:02	
		DQB1*06:281	
		DQB1*06:282	
		DQB1*06:299	
		DQB1*06:88	
#2907	DQB1*06	DQB1*06:04	DQB1*06:04:01
			DQB1*06:04:11
		DQB1*06:217	
		DQB1*06:254	
		DQB1*06:265	
		DQB1*06:267	
		DQB1*06:280	
		DQB1*06:283	
		DQB1*06:288	
		DQB1*06:291	
		DQB1*06:34	
		DQB1*06:36	
		DQB1*06:38	
		DQB1*06:39	
		DQB1*06:52	

		DQB1*06:69:01	
		DQB1*06:86	
#2908	DQB1*06	DQB1*06:01	DQB1*06:01:01
			DQB1*06:01:03
			DQB1*06:01:05
			DQB1*06:01:06
			DQB1*06:01:09
			DQB1*06:01:10
			DQB1*06:01:11
			DQB1*06:01:15
		DQB1*06:102N	
		DQB1*06:103	
		DQB1*06:104	
		DQB1*06:105	
		DQB1*06:108	
		DQB1*06:120	
		DQB1*06:205	
		DQB1*06:243	
		DQB1*06:258	
		DQB1*06:285	
#2909	DQB1*06	DQB1*06:03	DQB1*06:03:01
			DQB1*06:03:21
			DQB1*06:03:22
			DQB1*06:03:23
			DQB1*06:03:24
			DQB1*06:03:27
			DQB1*06:03:32
			DQB1*06:03:35
		DQB1*06:110	
		DQB1*06:14:01	
		DQB1*06:185	
		DQB1*06:187	
		DQB1*06:218	

		DQB1*06:221	
		DQB1*06:223	
		DQB1*06:230	
		DQB1*06:238	
		DQB1*06:244	
		DQB1*06:250	
		DQB1*06:253	
		DQB1*06:269	
		DQB1*06:272	
		DQB1*06:279	
		DQB1*06:41	
		DQB1*06:44	
**#2910**	**DQB1*05**	DQB1*05:119	

**HLA-DPB1 Exon 2 sequence number**	**(no 1st Field)**	**HLA-DPB1 Nominal Alleles**	
		**2nd field**	**3rd field**
**#62**		DPB1*05:01	DPB1*05:01:01
			DPB1*05:01:07
		DPB1*135:01	
		DPB1*668:01	
		DPB1*764:01	
**#63**		DPB1*04:02	DPB1*04:02:01
			DPB1*04:02:10
			DPB1*04:02:13
			DPB1*04:02:14
		DPB1*105:01:01	
		DPB1*463:01:01	
		DPB1*665:01	
		DPB1*701:01	
		DPB1*724:01N	
		DPB1*734:01	
		DPB1*735:01	
		DPB1*881:01	
**#64**		DPB1*131:01	
		DPB1*17:01:01	

	DPB1*460:01	
#65	DPB1*107:01	
	DPB1*13:01	DPB1*13:01:01
		DPB1*13:01:03
	DPB1*133:01	
	DPB1*518:01	
	DPB1*519:01	
#66	DPB1*01:01:01	
	DPB1*162:01:02	
	DPB1*733:01	
#67	DPB1*02:01	DPB1*02:01:02
		DPB1*02:01:19
		DPB1*02:01:20
		DPB1*02:01:22
		DPB1*02:01:25
		DPB1*02:01:37
	DPB1*141:01	
	DPB1*352:01:02	
	DPB1*414:01:01	
	DPB1*678:01	
	DPB1*700:01N	
	DPB1*786:01:01N	
	DPB1*819:01	
#68	DPB1*15:01:01	
	DPB1*585:01:01	
#69	DPB1*03:01	DPB1*03:01:01
		DPB1*03:01:10
	DPB1*104:01:01	
	DPB1*124:01:01	
	DPB1*669:01	
	DPB1*675:01	
	DPB1*676:01	
	DPB1*728:01	
#70	DPB1*85:01:01	
		DPB1*04:01:01

**#71**	DPB1*04:01	DPB1*04:01:31
		DPB1*04:01:33
		DPB1*04:01:34
		DPB1*04:01:36
		DPB1*04:01:37
		DPB1*04:01:38
		DPB1*04:01:39
	DPB1*126:01:01	
	DPB1*415:01	
	DPB1*464:01	
	DPB1*670:01	
	DPB1*699:01	
	DPB1*880:01	
	DPB1*882:01	
**#72**	DPB1*01:01:02	
**#73**	DPB1*40:01:01	
**#74**	DPB1*30:01:01	
**#75**	DPB1*63:01	
**#76**	DPB1*10:01:01	
	DPB1*650:01	
	DPB1*673:01	
**#77**	DPB1*11:01:01	
	DPB1*649:01	
	DPB1*672:01	
**#79**	DPB1*45:01	
	DPB1*832:01	
**#80**	DPB1*39:01	DPB1*39:01:01
		DPB1*39:01:02
		DPB1*39:01:03
	DPB1*584:01:01	
**#81**	DPB1*34:01:01	
	DPB1*835:01	
**#82**	DPB1*28:01	
	DPB1*296:01	
**#83**	DPB1*408:01	

#84	DPB1*14:01:01	
	DPB1*498:01	
	DPB1*572:01	
	DPB1*651:01	
	DPB1*671:01	
	DPB1*705:01	
	DPB1*834:01	
#85	DPB1*18:01:01	
#86	DPB1*09:01:01	
#89	DPB1*138:01	
	DPB1*23:01:01	
#92	DPB1*04:01	DPB1*04:01:01
		DPB1*04:01:31
		DPB1*04:01:33
		DPB1*04:01:34
		DPB1*04:01:36
		DPB1*04:01:37
		DPB1*04:01:38
		DPB1*04:01:39
	DPB1*126:01:01	
	DPB1*415:01	
	DPB1*464:01	
	DPB1*670:01	
	DPB1*699:01	
	DPB1*880:01	
	DPB1*882:01	
#94	DPB1*106:01	
	DPB1*19:01:01	
	DPB1*535:01	
#97	DPB1*35:01:01	
#101	DPB1*06:01	DPB1*06:01:01
		DPB1*06:01:04
	DPB1*737:01	
#107	DPB1*49:01:01	
	DPB1*16:01:01	

**#108**		DPB1*652:01
		DPB1*653:01

Supplementary Table S4: Sequences lengths

	DRB1 (Exon2)	DQA1 (Exon2)	DQB1 (Exon2)	DPB1 (Exon2)
**Whole Exon 2**	241	217	242	264
**ARS**	69	60	72	75
**Non-ARS**	171	153	168	186
**5' cover**	4	0	16	0
**3' cover**	25	32	12	0

Lengths (in nucleotides) of the available sequences according to the three nucleotide sets: Whole Exon 2, only nucleotides coding for the ARS (ARS) and only nucleotide not coding for the ARS (non-ARS). 5' cover (resp. 3' cover) gives the number of position not available in 5' (resp. 3') in the full sequence. Note that the sum of ARS and non-ARS for a locus is less than the total sequence size, because this sequence usually starts and ends in the middle of a codon (which is then excluded from the ARS/non-ARS sets).

Supplementary Table S5: Genetic profiles of populations at each locus

DRB1

Region	Population	Sample size	Hardy-Weinberg pValue	DRB1#3135	DRB1#3136	DRB1#3137	DRB1#3138	DRB1#3139	DRB1#3140	DRB1#3142	DRB1#3143	DRB1#3144	DRB1#3145	DRB1#3147	DRB1#3148	DRB1#3149	DRB1#3150	DRB1#3151
C-AFR	BAG	49	0.09	–	0.06	0.15	0.04	0.02	0.07	0.04	0.01	–	0.13	0.02	0.06	0.04	–	–
C-AFR	DAZ	39	0.06	–	0.1	0.09	–	0.01	–	0.28	–	–	0.01	–	0.08	0.01	0.01	0.01
C-AFR	DAN	49	0.27	–	0.05	0.04	–	–	–	0.11	–	–	0.23	0.02	0.01	0.06	0.03	0.06
C-AFR	MAB	41	0.94	–	0.11	0.17	0.01	0.02	–	0.05	–	–	0.02	0.1	–	0.01	–	0.09
E-AFR	AMH	48	0.02*	–	0.12	0.17	0.04	–	0.03	0.2	–	–	–	0.14	0.1	–	–	–
E-AFR	BEJ	47	0.01*	–	0.35	0.12	0.04	–	0.02	0.15	–	–	0.01	0.05	0.02	–	–	0.02
E-AFR	NUB	49	0.90	–	0.14	0.07	0.03	0.01	0.02	0.14	0.01	–	0.03	0.05	0.03	0.02	–	0.07
E-AFR	ORO	21	0.06	–	0.14	0.1	0.05	–	–	0.38	–	–	0.02	–	0.05	–	–	0.1
E-AFR	RAS	39	0.93	0.01	0.36	0.23	0.01	–	0.13	0.05	–	–	0.01	0.03	0.01	–	–	–
E-AFR	SUD	37	0.93	–	0.14	0.12	0.04	0.01	0.04	0.09	0.01	–	0.08	0.05	0.03	–	–	0.04
N-AFR	_ALA_	_108_	_<0.001***_		_0.13_	_0.23_	_0.04_	_0.03_	_0.09_	_0.04_	–	_0.01_	_0.06_	_0.03_	_0.04_	_0.01_	_0.01_	_0.03_
N-AFR	ALC	42	<0.001***	–	0.08	0.18	0.02	–	0.1	0.1	0.01	–	0.08	0.01	0.08	0.01	0.01	0.02
N-AFR	_ALG_	_83_	_0.004**_		_0.07_	_0.04_	–	_0.08_	_0.07_	_0.01_	–	_0.01_	_0.16_	_0.08_	_0.06_	_0.01_	_0.01_	_0.01_
N-AFR	ALT	28	0.48	–	0.07	0.12	0.09	–	0.09	0.09	–	–	0.05	0.09	0.05	0.09	–	0.05
N-AFR	AMI	50	0.02*	–	0.2	0.18	0.02	0.01	0.17	0.07	–	–	0.03	–	0.01	–	–	0.03
N-AFR	ASN	48	0.71	–	0.16	0.24	0.03	–	0.09	0.1	–	0.02	0.06	0.03	0.03	0.02	–	0.03
N-AFR	FIG	97	0.05	0.01	0.13	0.2	0.02	0.04	0.09	0.08	–	–	0.02	0.06	0.03	0.01	–	0.01
W-AFR	BED	45	0.63	0.3	–	0.04	0.02	–	0.01	0.09	0.04	0.01	0.28	0.01	0.01	0.02	0.08	0.01
W-AFR	_DOG_	_147_	_<0.001***_		_0.03_	_0.12_	_0.01_	_0.01_	–	_0.03_	_0.03_	_0.06_	_0.09_	_0.04_	_0.13_	_0.05_	_0.02_	_0.07_
W-AFR	GRS	32	0.96	–	0.08	0.06	0.03	–	–	0.06	0.02	0.06	0.08	–	0.12	0.08	–	0.05
W-AFR	GUR	32	0.49	–	0.08	0.06	–	–	–	0.06	–	–	0.11	0.06	0.09	0.12	–	0.03
W-AFR	MAF	67	0.11	–	0.08	0.30	–	–	–	0.07	–	–	0.02	0.19	0.04	0.01	0.02	0.02
W-AFR	MAN	199	0.12	0.27	0.08	0.1	0.01	0.01	–	0.05	0.04	0.04	0.11	0.03	0.03	0.04	0	0.05
W-AFR	MOS	34	0.04*	–	0.07	0.06	0.01	–	–	0.15	0.03	0.04	0.1	0.07	0.09	0.07	0.03	0.06
W-AFR	SEF	56	0.93	0.02	0.07	0.07	0.12	–	–	0.04	0.01	–	0.04	0.12	0.01	0.05	0.02	0.19
W-AFR	SRR	47	<0.001***	0.29	–	0.05	0.04	0.01	–	0.07	–	0.01	0.05	0.07	0.04	0.06	0.11	0.13

Region	Population	DRB1#3152	DRB1#3153	DRB1#3154	DRB1#3155	DRB1#3156	DRB1#3157	DRB1#3158	DRB1#3159	DRB1#3160	DRB1#3161	DRB1#3163	DRB1#3164	DRB1#3165	DRB1#3167	DRB1#3168	DRB1#3169	DRB1#3170	DRB1#3172	DRB1#3173	DRB1#3183
C-AFR	BAG	0.03	0.2	0.01	0.05	0.05	–	–	–	–	–	–	–	–	–	–	–	–	–	–	–
C-AFR	DAZ	0.03	0.19	–	0.08	0.01	–	–	–	–	0.01	–	0.05	–	–	–	–	–	–	0.01	–
C-AFR	DAN	0.05	0.13	–	0.1	0.04	–	–	–	–	0.05	–	–	–	–	–	–	–	–	–	–
C-AFR	MAB	0.01	0.27	0.01	0.04	0.04	–	–	0.01	0.04	–	–	–	–	–	–	–	–	–	–	–
E-AFR	AMH	0.06	0.09	–	–	–	–	–	–	–	0.01	–	–	–	–	–	0.03	–	–	–	–
E-AFR	BEJ	0.06	0.03	0.04	0.01	–	–	–	–	–	–	0.03	0.02	–	0.01	–	–	–	–	–	–
E-AFR	NUB	0.09	0.05	0.06	0.03	0.01	–	–	0.03	–	0.06	–	0.01	0.01	–	–	–	0.01	–	–	–
E-AFR	ORO	0.07	0.05	–	–	–	–	–	–	–	0.05	–	–	–	–	–	–	–	–	–	–
E-AFR	RAS	0.01	–	–	–	–	–	–	–	0.01	0.03	–	0.04	0.05	0.01	–	–	–	–	–	–
E-AFR	SUD	0.04	0.04	0.04	–	–	–	–	0.08	0.04	0.04	0.01	–	0.03	–	–	–	–	0.01	–	–
N-AFR	_ALA_	_0.01_	_0.02_	_0.04_	_0.02_	_0.01_	–	_0.01_	_0.03_	_0.05_	_0.01_	_0.02_	_0.01_	_0.04_	–	–	_0.01_	–	–	–	–
N-AFR	ALC	0.01	–	0.08	0.02	0.04	0.01	–	0.01	0.05	–	0.02	–	0.01	–	–	0.02	–	–	–	–
N-AFR	_ALG_	–	_0.01_	_0.07_	_0.09_	_0.01_	–	_0.03_	_0.01_	_0.01_	_0.01_	–	_0.01_	_0.02_	–	–	–	–	–	–	–
N-AFR	ALT	0.07	0.04	0.02	0.02	–	–	–	–	–	0.05	–	–	–	–	–	–	–	–	–	–
N-AFR	AMI	–	–	0.03	0.02	–	–	0.04	0.02	0.01	0.07	0.04	0.03	0.02	–	–	–	–	–	–	–
N-AFR	ASN	–	0.02	0.02	0.02	–	–	–	0.02	–	0.03	0.04	–	0.01	–	–	–	0.01	–	–	–
N-AFR	FIG	0.01	–	0.1	0.03	0.01	0.02	0.01	0.01	0.05	–	0.07	0.01	0.01	–	–	–	–	–	–	0.01
W-AFR	BED	–	0.06	–	0.01	–	–	–	–	–	–	–	–	–	–	–	–	–	–	–	–
W-AFR	_DOG_	_0.03_	_0.15_	–	_0.11_	_0.01_	–	–	–	–	–	–	–	–	–	–	–	–	–	–	–
W-AFR	GRS	0.06	0.11	–	0.14	0.03	–	–	–	–	0.02	–	–	–	–	–	–	–	–	–	–
W-AFR	GUR	0.02	0.19	0.03	0.11	0.03	–	–	–	–	–	–	–	–	–	–	–	–	–	–	–
W-AFR	MAF	0.02	0.07	0.01	0.03	–	–	–	–	–	0.01	–	–	–	–	0.01	–	–	–	–	–
W-AFR	MAN	–	0.08	–	0.08	0	–	–	–	–	0	–	–	–	–	0	–	–	–	–	–
W-AFR	MOS	0.07	0.04	–	0.07	0.01	–	–	–	–	–	–	–	–	–	–	–	–	–	–	–
W-AFR	SEF	0.01	0.1	–	0.12	–	–	–	–	–	0.01	–	0.01	–	–	–	–	–	–	–	–
W-AFR	SRR	–	0.01	–	0.04	–	–	–	–	–	–	–	–	–	–	–	–	–	–	–	–

DQA1

Region	Population	Sample size	Hardy-Weinberg pValue	DQA1#1	DQA1#2	DQA1#3	DQA1#4	DQA1#5	DQA1#6	DQA1#8	DQA1#9	DQA1#10	DQA1#12
C-AFR	BAG	51	0.55	0.35	0.09	0.07	0.07	0.04	0.17	0.13	0.03	0.05	0.01
C-AFR	DAZ	40	0.91	0.36	0.24	–	0.09	0.05	0.05	0.09	0.09	0.04	–
C-AFR	DAN	49	0.91	0.29	0.15	0.02	0.09	0.06	0.24	0.05	0.05	0.03	0.01
C-AFR	MAB	42	0.06	0.61	0.01	0.05	0.04	0.04	0.14	0.01	0.04	0.07	–
E-AFR	AMH	82	0.85	0.34	0.15	0.07	0.12	0.05	0.05	0.1	0.11	0.02	–
E-AFR	BEJ	45	0.88	0.22	0.17	0.03	0.09	0.02	0.09	0.3	0.04	0.03	–
E-AFR	NUB	52	0.20	0.25	0.14	0.07	0.11	0.05	0.13	0.13	0.04	0.08	–
E-AFR	ORO	31	0.14	0.27	0.26	0.03	0.08	–	0.06	0.19	0.06	0.03	–
E-AFR	RAS	50	0.43	0.23	0.08	0.06	0.11	–	0.2	0.3	0.01	0.01	–
E-AFR	SUD	46	0.01*	0.33	0.08	0.13	0.11	0.01	0.13	0.1	0.04	0.08	–
N-AFR	_ALA_	_113_	_0.36_	_0.35_	_0.04_	_0.05_	_0.13_	_0.01_	_0.14_	_0.012_	_0.08_	_0.08_	–
N-AFR	ALC	41	0.09	0.39	0.09	0.02	0.13	0.01	0.13	0.07	0.11	0.04	–
N-AFR	_ALG_	_80_	_0.81_	_0.46_	_0.03_	_0.08_	_0.04_	_0.05_	_0.13_	_0.07_	_0.04_	_0.10_	–
N-AFR	ALT	32	0.97	0.28	0.11	0.06	0.08	0.11	0.16	0.11	0.05	0.05	–
N-AFR	AMI	49	0.93	0.24	0.09	0.02	0.16	0.03	0.17	0.16	0.09	0.02	–
N-AFR	ASN	50	0.98	0.35	0.11	0.04	0.1	0.03	0.13	0.17	0.04	0.03	–
N-AFR	FIG	92	<0.001***	0.36	0.09	0.07	0.15	0.02	0.1	0.14	0.02	0.05	–
W-AFR	BED	39	0.96	0.56	0.12	0.01	0.13	0.01	0.12	–	0.04	0.01	–
W-AFR	_DOG_	_150_	_0.06_	_0.53_	_0.05_	_0.03_	_0.05_	_0.1_	_0.08_	_0.03_	_0.04_	_0.09_	–
W-AFR	GRS	33	0.99	0.45	0.06	–	0.06	0.17	0.11	0.08	0.02	0.06	–
W-AFR	GUR	37	0.25	0.42	0.15	0.05	0.01	0.14	0.08	0.07	0.07	0.01	–
W-AFR	MAF	51	0.92	0.39	0.07	0.22	0.11	0.08	0.04	0.1	–	–	–
W-AFR	MAN	197	0.87	0.59	0.06	0.01	0.02	0.07	0.11	0.08	0.01	0.06	–
W-AFR	MOS	35	0.72	0.34	0.14	0.1	0.03	0.06	0.17	0.07	0.03	0.06	–
W-AFR	SEF	55	0.83	0.24	0.04	0.13	0.17	0.13	0.07	0.06	–	0.16	–
W-AFR	SRR	47	0.95	0.43	0.12	0.03	0.15	0.07	0.03	–	0.02	0.15	–

DQB1

Region	Population	Sample size	Hardy-Weinberg pValue	DQB1#2899	DQB1#2900	DQB1#2901	DQB1#2902	DQB1#2903	DQB1#2904	DQB1#2905	DQB1#2906	DQB1#2907	DQB1#2908	DQB1#2909	DQB1#2910
C-AFR	BAG	51	0.03*	0.05	0.11	0.39	0.19	0.04	0.02	0.01	0.05	0.06	0.01	0.08	–
C-AFR	DAZ	41	0.24	–	0.11	0.21	0.34	0.07	0.01	–	0.05	0.18	–	0.02	–
C-AFR	DAN	49	0.32	0.05	0.3	0.15	0.26	0.06	0.06	–	0.04	0.06	–	0.02	–
C-AFR	MAB	42	0.03*	0.04	0.19	0.1	0.56	0.02	0.04	–	0.02	–	–	0.04	–
E-AFR	AMH	66	0.17	0.05	0.12	0.27	0.17	0.06	0.05	0.02	0.1	0.08	–	0.07	–
E-AFR	BEJ	48	0.54	0.06	0.07	0.49	0.08	0.05	0.02	0.03	0.02	0.11	–	0.05	–
E-AFR	NUB	51	0.27	0.06	0.12	0.25	0.23	0.04	0.07	0.01	0.01	0.12	0.03	0.08	–
E-AFR	ORO	23	0.54	0.02	0.13	0.35	0.15	–	0.04	–	0.04	0.22	–	0.04	–
E-AFR	RAS	45	0.01*	0.13	0.02	0.57	0.06	–	0.07	0.01	–	0.04	0.04	0.06	–
E-AFR	SUD	46	0.12	0.08	0.1	0.22	0.22	0.02	0.1	0.03	0.01	0.07	0.07	0.1	–
N-AFR	_ALA_	_109_	_<0.001***_	_0.09_	_0.15_	_0.37_	_0.14_	_0.02_	_0.12_	_0.01_	_0.02_	_0.01_	_0.02_	_0.03_	–
N-AFR	ALC	44	0.02*	0.09	0.17	0.24	0.3	0.07	0.06	–	–	0.05	0.01	0.02	–
N-AFR	_ALG_	_82_	_<0.001***_	_0.09_	_0.07_	_0.27_	_0.39_	_0.04_	_0.03_	–	_0.01_	_0.01_	_0.01_	_0.09_	–
N-AFR	ALT	30	0.95	0.13	0.15	0.23	0.18	0.12	0.03	–	–	0.08	–	0.07	–
N-AFR	AMI	25	0.98	0.1	0.16	0.38	0.1	0.1	0.08	0.02	0.02	0.02	0.02	–	–
N-AFR	_ASN_	_0_	–	_Allele frequencies not computed due to limited sample size_											
N-AFR	FIG	79	0.38	0.05	0.06	0.35	0.24	0.09	0.08	0.02	–	0.08	–	0.01	0.02
W-AFR	BED	3	–	_Allele frequencies not computed due to limited sample size_											
W-AFR	_DOG_	_143_	_<0.001***_	_0.04_	_0.13_	_0.20_	_0.48_	_0.06_	_0.01_	–	_0.04_	–	–	_0.04_	–
W-AFR	GRS	33	0.96	0.06	0.08	0.17	0.53	0.09	0.05	–	0.03	–	–	–	–
W-AFR	GUR	36	0.94	0.03	0.14	0.17	0.47	0.11	–	–	0.04	–	–	0.04	–
W-AFR	_MAF_	_18_	_0.83_	–	_0.09_	_0.58_	_0.22_	–	–	–	–	_0.09_	–	_0.03_	–
W-AFR	MAN	195	0.62	0.06	0.11	0.2	0.54	0.04	0.02	–	0.02	0	–	0.01	0
W-AFR	MOS	35	0.03*	0.11	0.14	0.16	0.29	0.06	0.01	–	0.09	0.01	–	0.13	–
W-AFR	SEF	52	0.93	0.01	0.22	0.26	0.26	0.06	0.04	–	0.01	0.07	–	0.08	–
W-AFR	SRR	48	0.89	0.03	0.24	0.18	0.4	0.06	0.03	–	0.01	0.01	–	0.04	–

DPB1

Region	Population	Sample size	Hardy-Weinberg pValue	DPB1#62	DPB1#63	DPB1#64	DPB1#65	DPB1#66	DPB1#67	DPB1#68	DPB1#69	DPB1#70	DPB1#71	DPB1#72	DPB1#73	DPB1#74
C-AFR	BAG	49	0.06	–	0.02	0.1	0.02	0.05	0.33	0.05	0.08	–	0.21	0.03	0.01	0.01
C-AFR	DAZ	40	0.91	–	0.08	0.11	0.02	0.01	0.38	0.02	0.09	–	0.16	0.02	–	–
C-AFR	DAN	50	0.90	–	0.14	0.06	0.06	0.31	0.12	–	0.1	–	0.04	0.08	–	–
C-AFR	MAB	38	0.07	–	0.13	0.09	0.03	0.04	0.46	–	0.07	–	0.12	0.01	–	–
E-AFR	AMH	73	0.95	–	0.05	0.09	0.02	–	0.26	0.05	0.16	–	0.25	0.01	–	0.01
E-AFR	BEJ	48	0.05	–	0.04	0.12	0.03	–	0.24	0.02	0.16	–	0.18	0.11	–	0.05
E-AFR	NUB	54	0.21	–	0.13	0.08	0.01	0.03	0.26	0.04	0.09	–	0.21	0.04	–	0.05
E-AFR	ORO	33	0.89	–	0.03	0.03	0.03	–	0.32	0.08	0.11	–	0.2	0.03	–	0.02
E-AFR	RAS	48	0.95	–	0.04	0.01	–	0.02	0.1	–	0.28	–	0.46	–	–	0.01
E-AFR	SUD	45	0.35	–	0.08	0.08	0.01	–	0.26	0.04	0.1	–	0.22	0.02	–	0.03
N-AFR	_ALA_	_111_	_0.79_	_0.03_	_0.10_	_0.06_	_0.07_	_0.05_	_0.09_	_0.01_	_0.15_	–	_0.35_	_0.02_	–	–
N-AFR	ALC	42	0.93	0.04	0.11	0.11	0.07	0.04	0.17	–	0.07	0.01	0.25	0.01	–	–
N-AFR	_ALG_	_82_	_0.47_	_0.05_	_0.09_	_0.09_	_0.07_	_0.07_	_0.09_	_0.06_	_0.20_	–	_0.16_	–	–	–
N-AFR	ALT	32	0.37	0.02	0.12	0.08	–	0.19	0.14	–	0.06	–	0.27	–	–	0.02
N-AFR	AMI	48	0.98	0.03	0.02	0.11	0.04	0.06	0.08	0.01	0.11	–	0.44	0.01	–	–
N-AFR	ASN	45	0.99	0.02	0.03	0.06	0.03	0.17	0.14	–	0.09	–	0.36	0.01	–	–
N-AFR	FIG	94	0.04*	0.01	0.13	0.06	0.01	0.06	0.08	–	0.14	–	0.41	–	–	–
W-AFR	BED	48	0.93	–	0.09	0.28	0.19	0.17	0.09	–	–	0.05	0.04	0.04	0.01	–
W-AFR	_DOG_	_151_	_0.87_	–	_0.08_	_0.19_	_0.04_	_0.39_	_0.15_	–	_0.02_	_0.01_	_0.02_	_0.04_	–	_0.01_
W-AFR	GRS	33	0.98	–	0.02	0.14	0.02	0.64	0.02	–	0.02	–	0.06	0.02	0.02	–
W-AFR	GUR	34	0.89	–	0.12	0.12	0.01	0.49	0.07	0.01	0.03	0.06	–	0.04	–	–
W-AFR	MAF	61	0.87	–	0.18	0.08	0.07	0.18	0.07	0.04	0.05	–	0.05	0.01	0.01	0.01
W-AFR	MAN	196	0.89	–	0.12	0.35	0.06	0.12	0.18	0.01	0.04	0.03	0.06	0.02	0.01	–
W-AFR	MOS	34	0.85	–	0.03	0.04	0.07	0.6	0.1	–	0.01	–	0.06	0.06	–	0.01
W-AFR	SEF	56	0.40	–	0.15	0.24	0.1	0.13	0.1	0.05	0.03	0.01	–	0.01	–	0.01
W-AFR	SRR	47	0.96	–	0.07	0.33	0.06	0.29	0.14	–	0.06	0.01	–	–	–	0.01

Region	Population	DPB1#75	DPB1#76	DPB1#77	DPB1#79	DPB1#80	DPB1#81	DPB1#82	DPB1#83	DPB1#84	DPB1#85	DPB1#86	DPB1#89	DPB1#92	DPB1#94	DPB1#97	DPB1#101	DPB1#107	DPB1#108
C-AFR	BAG	–	–	0.02	–	–	–	–	–	0.05	–	–	–	–	0.01	–	–	–	–
C-AFR	DAZ	–	–	0.01	–	–	0.05	–	0.02	0.01	–	–	–	–	–	–	–	–	–
C-AFR	DAN	–	–	0.03	–	–	–	–	–	0.05	–	–	–	–	–	–	–	0.01	–
C-AFR	MAB	–	–	0.05	–	–	–	–	–	–	–	–	–	–	–	–	–	–	–
E-AFR	AMH	–	–	0.03	–	0.02	–	–	–	0.01	–	0.02	–	–	–	–	–	–	–
E-AFR	BEJ	–	–	0.04	–	–	–	–	–	–	–	–	–	–	–	–	–	–	–
E-AFR	NUB	–	0.02	0.03	–	0.01	–	–	–	0.01	–	–	–	–	–	–	–	–	–
E-AFR	ORO	–	–	0.08	–	0.03	0.03	–	–	–	–	0.03	–	–	–	–	–	–	–
E-AFR	RAS	–	0.02	–	–	–	–	0.03	–	0.01	–	0.01	–	–	–	–	–	–	–
E-AFR	SUD	–	0.01	0.04	–	–	0.01	–	0.01	0.02	–	–	0.01	–	0.02	–	–	–	0.02
N-AFR	_ALA_	–	_0.04_	_0.01_	–	–	_0.01_	–	–	_0.03_	–	–	–	–	–	–	–	–	–
N-AFR	ALC	0.02	0.01	0.02	–	–	–	–	–	0.01	–	–	0.04	0.01	–	–	0.01	–	–
N-AFR	_ALG_	–	_0.10_	_0.01_	–	–	–	–	–	_0.01_	–	–	–	–	–	–	–	–	–
N-AFR	ALT	–	0.03	0.05	–	–	0.03	–	–	–	–	–	–	–	–	–	–	–	–
N-AFR	AMI	–	0.03	–	0.01	–	0.02	–	–	–	–	0.01	–	–	–	–	–	–	–
N-AFR	ASN	–	0.08	0.01	–	–	–	–	–	–	–	–	–	–	–	–	–	–	–
N-AFR	FIG	0.01	0.07	–	–	–	0.01	–	–	–	–	–	–	0.01	–	–	–	–	–
W-AFR	BED	–	0.01	–	–	–	–	–	–	–	–	–	–	–	–	0.01	–	0.01	–
W-AFR	_DOG_	–	_0.01_	_0.03_	–	–	–	–	–	–	–	–	–	–	–	_0.01_	–	–	–
W-AFR	GRS	–	–	0.08	–	–	–	–	–	–	–	–	–	–	–	–	–	–	–
W-AFR	GUR	–	–	0.03	–	–	–	–	–	–	0.01	–	–	–	–	–	–	–	–
W-AFR	MAF	–	0.23	0.01	–	–	0.02	–	–	–	–	–	–	–	–	–	–	–	–
W-AFR	MAN	–	0	0.01	–	0.01	–	–	–	–	–	–	–	–	–	–	–	–	–
W-AFR	MOS	–	–	–	–	–	–	–	–	–	–	–	–	–	–	–	–	–	–
W-AFR	SEF	–	0.13	–	–	–	0.04	–	–	–	–	–	–	–	–	–	–	–	–
W-AFR	SRR	–	–	0.02	–	–	–	–	–	–	–	–	–	–	–	–	–	–	–

For each population and each locus, this table provides: the geographic region where these populations dwell (W-AFR: West Africa; C-AFR: Central Africa; E-AFR: East-Africa and N-AFR: North Africa), their short name (see Supplementary Table S1), the pValue of the Hardy-Weinberg equilibrium test (* p ≤ 0.05; ** p ≤ 0.01; *** p ≤ 0.001), the observed heterozygosity (Observed Het.) and the expected heterozygosity under the assumption of Hardy-Weinberg equilibrium (Expected Het.). The following columns correspond to the estimated frequencies of the observed HLA sequences, « – » indicates a sequence not observed in this population. The frequencies shown in italics have not been used in the analyses either because the populations have been considered in HW disequilibrium due to HWE rejection at more than one locus (ALA, ALG, DOG) or because the sample sizes at the corresponding loci are too low (< 20 individuals tested).

Supplementary Table S6 : Frequencies of haplotypes in linkage disequilebrium (LD). For each of the 20 populations, this table provides the observed and expected frequencies (resp. F.obs and F.exp) for the two locus-haplotypes in significant LD. LD was assesed using Gene[rate] tools (Nunes et al., 2014, Nunes 2016) and only haplotypes with an F.obs > 5 and standardized residuals (STRDES) above 1.96 were considered in significant positive LD.

Region	Population	Short name	Haplotype			F.obs	F.exp	diff.	STDRES
W-AFR	Senegal-Mandenka	MAN	DQA1#8	~	DQB1#2901	0.081	0.016	0.065	9.919
W-AFR	Senegal-Mandenka	MAN	DRB1#3136	~	DQB1#2901	0.073	0.015	0.058	9.185
W-AFR	Senegal-Mandenka	MAN	DRB1#3135	~	DQB1#2902	0.263	0.144	0.119	5.666
W-AFR	Senegal-Mandenka	MAN	DRB1#3135	~	DPB1#64	0.186	0.094	0.092	5.608
W-AFR	Senegal-Mandenka	MAN	DRB1#3135	~	DQA1#1	0.267	0.157	0.110	4.965
W-AFR	Senegal-Mandenka	MAN	DQA1#1	~	DQB1#2902	0.484	0.318	0.167	4.774
W-AFR	Senegal-Mandenka	MAN	DQB1#2902	~	DPB1#64	0.258	0.189	0.069	2.801
W-AFR	Senegal-Mandenka	MAN	DRB1#3136	~	DQA1#8	0.073	0.006	0.067	16.663
W-AFR	Senegal-Mandenka	MAN	DQA1#6	~	DQB1#2899	0.063	0.007	0.056	13.507
W-AFR	Senegal-Mandenka	MAN	DRB1#3137	~	DQB1#2901	0.100	0.020	0.080	10.960
W-AFR	Senegal-Serere	SRR	DRB1#3149	~	DQB1#2903	0.067	0.004	0.062	8.834
W-AFR	Senegal-Serere	SRR	DQA1#5	~	DQB1#2903	0.067	0.005	0.062	8.080
W-AFR	Senegal-Serere	SRR	DRB1#3149	~	DQA1#5	0.067	0.005	0.062	8.080
W-AFR	Senegal-Serere	SRR	DRB1#3151	~	DQA1#10	0.133	0.021	0.113	7.337
W-AFR	Senegal-Serere	SRR	DRB1#3150	~	DQA1#4	0.100	0.013	0.087	7.074
W-AFR	Senegal-Serere	SRR	DRB1#3142	~	DQA1#2	0.078	0.010	0.068	6.613
W-AFR	Senegal-Serere	SRR	DRB1#3150	~	DQB1#2901	0.100	0.017	0.083	6.072
W-AFR	Senegal-Serere	SRR	DRB1#3151	~	DQB1#2900	0.133	0.034	0.099	5.012
W-AFR	Senegal-Serere	SRR	DQA1#4	~	DQB1#2901	0.100	0.022	0.078	4.895
W-AFR	Senegal-Serere	SRR	DQA1#1	~	DQB1#2902	0.367	0.160	0.207	4.512
W-AFR	Senegal-Serere	SRR	DRB1#3135	~	DQB1#2902	0.267	0.105	0.162	4.481
W-AFR	Senegal-Serere	SRR	DQA1#10	~	DQB1#2900	0.133	0.040	0.094	4.360
W-AFR	Senegal-Serere	SRR	DRB1#3135	~	DQA1#1	0.278	0.117	0.161	4.177
W-AFR	Senegal-Serere	SRR	DQA1#2	~	DQB1#2900	0.100	0.031	0.069	3.633
W-AFR	Senegal-Serere	SRR	DQA1#2	~	DPB1#66	0.100	0.037	0.063	3.066
W-AFR	Senegal-Serere	SRR	DQB1#2900	~	DPB1#66	0.163	0.077	0.086	2.825
W-AFR	Senegal-Serere	SRR	DRB1#3135	~	DPB1#64	0.173	0.090	0.084	2.538
W-AFR	Senegal-Serere	SRR	DQA1#1	~	DPB1#64	0.233	0.136	0.097	2.321
W-AFR	Senegal-Fulani	SEF	DRB1#3149	~	DQB1#2903	0.059	0.003	0.055	9.489
W-AFR	Senegal-Fulani	SEF	DRB1#3136	~	DPB1#68	0.059	0.004	0.055	8.691
W-AFR	Senegal-Fulani	SEF	DRB1#3151	~	DQB1#2900	0.196	0.044	0.152	7.131
W-AFR	Senegal-Fulani	SEF	DRB1#3147	~	DQB1#2909	0.069	0.008	0.061	6.779
W-AFR	Senegal-Fulani	SEF	DRB1#3155	~	DPB1#66	0.088	0.016	0.072	5.665
W-AFR	Senegal-Fulani	SEF	DRB1#3147	~	DQA1#3	0.088	0.016	0.072	5.649
W-AFR	Senegal-Fulani	SEF	DRB1#3155	~	DQB1#2902	0.118	0.031	0.086	4.872
W-AFR	Senegal-Fulani	SEF	DRB1#3153	~	DQB1#2902	0.098	0.026	0.072	4.460
W-AFR	Senegal-Fulani	SEF	DRB1#3138	~	DQA1#4	0.078	0.019	0.059	4.255
W-AFR	Senegal-Fulani	SEF	DRB1#3138	~	DPB1#63	0.069	0.016	0.053	4.196
W-AFR	Senegal-Fulani	SEF	DRB1#3147	~	DPB1#63	0.068	0.017	0.051	3.890
W-AFR	Senegal-Fulani	SEF	DRB1#3136	~	DQB1#2901	0.069	0.017	0.051	3.870
W-AFR	Senegal-Fulani	SEF	DRB1#3151	~	DQA1#10	0.102	0.033	0.070	3.830
W-AFR	Senegal-Fulani	SEF	DQA1#1	~	DQB1#2902	0.145	0.055	0.090	3.794
W-AFR	Senegal-Fulani	SEF	DRB1#3138	~	DQB1#2901	0.088	0.027	0.061	3.648
W-AFR	Senegal-Fulani	SEF	DQB1#2902	~	DPB1#66	0.097	0.036	0.061	3.165
W-AFR	Senegal-Fulani	SEF	DQA1#10	~	DQB1#2900	0.093	0.038	0.055	2.821
W-AFR	Senegal-Fulani	SEF	DQA1#4	~	DQB1#2901	0.105	0.045	0.060	2.797
W-AFR	Senegal-Fulani	SEF	DQB1#2900	~	DPB1#64	0.108	0.057	0.050	2.058
W-AFR	BurkinaFaso-Gurmantche	GUR	DRB1#3149	~	DQB1#2903	0.109	0.014	0.096	6.504
W-AFR	BurkinaFaso-Gurmantche	GUR	DRB1#3136	~	DQA1#8	0.063	0.006	0.056	5.760
W-AFR	BurkinaFaso-Gurmantche	GUR	DRB1#3136	~	DQB1#2901	0.078	0.013	0.065	4.435
W-AFR	BurkinaFaso-Gurmantche	GUR	DQA1#8	~	DQB1#2901	0.078	0.013	0.065	4.435
W-AFR	BurkinaFaso-Gurmantche	GUR	DRB1#3142	~	DQA1#2	0.063	0.011	0.052	3.973
W-AFR	BurkinaFaso-Gurmantche	GUR	DRB1#3137	~	DQB1#2901	0.063	0.011	0.052	3.973
W-AFR	BurkinaFaso-Gurmantche	GUR	DRB1#3153	~	DQB1#2902	0.172	0.091	0.081	2.052
W-AFR	BurkinaFaso-Gurunsi	GRS	DRB1#3142	~	DQA1#2	0.063	0.004	0.059	7.485
W-AFR	BurkinaFaso-Gurunsi	GRS	DRB1#3152	~	DQB1#2899	0.063	0.004	0.059	7.485
W-AFR	BurkinaFaso-Gurunsi	GRS	DRB1#3136	~	DQA1#8	0.078	0.006	0.072	7.353
W-AFR	BurkinaFaso-Gurunsi	GRS	DRB1#3149	~	DQB1#2903	0.078	0.007	0.071	6.591
W-AFR	BurkinaFaso-Gurunsi	GRS	DQA1#6	~	DQB1#2899	0.063	0.007	0.056	5.367

W-AFR	BurkinaFaso-Gurunsi	GRS	DRB1#3152	~	DQA1#6	0.063	0.007	0.056	5.367
W-AFR	BurkinaFaso-Gurunsi	GRS	DQA1#5	~	DQB1#2903	0.094	0.016	0.078	4.848
W-AFR	BurkinaFaso-Gurunsi	GRS	DQA1#8	~	DQB1#2901	0.078	0.013	0.065	4.435
W-AFR	BurkinaFaso-Gurunsi	GRS	DRB1#3149	~	DQA1#5	0.078	0.013	0.065	4.435
W-AFR	BurkinaFaso-Gurunsi	GRS	DRB1#3136	~	DQB1#2901	0.078	0.013	0.065	4.435
W-AFR	BurkinaFaso-Gurunsi	GRS	DRB1#3153	~	DQA1#5	0.094	0.019	0.075	4.328
W-AFR	BurkinaFaso-Gurunsi	GRS	DRB1#3137	~	DQB1#2901	0.063	0.011	0.052	3.973
W-AFR	BurkinaFaso-Gurunsi	GRS	DRB1#3155	~	DQA1#1	0.141	0.064	0.077	2.358
W-AFR	BurkinaFaso-Gurunsi	GRS	DQA1#1	~	DQB1#2902	0.391	0.234	0.157	2.275
W-AFR	BurkinaFaso-Mossi	MOS	DQA1#5	~	DQB1#2903	0.063	0.004	0.059	7.485
W-AFR	BurkinaFaso-Mossi	MOS	DRB1#3151	~	DQA1#10	0.063	0.004	0.059	7.485
W-AFR	BurkinaFaso-Mossi	MOS	DRB1#3136	~	DQA1#8	0.078	0.006	0.072	7.353
W-AFR	BurkinaFaso-Mossi	MOS	DRB1#3142	~	DQA1#2	0.156	0.024	0.132	6.668
W-AFR	BurkinaFaso-Mossi	MOS	DRB1#3147	~	DQA1#3	0.078	0.007	0.071	6.591
W-AFR	BurkinaFaso-Mossi	MOS	DRB1#3149	~	DQB1#2903	0.063	0.005	0.058	6.582
W-AFR	BurkinaFaso-Mossi	MOS	DRB1#3149	~	DQA1#5	0.063	0.005	0.058	6.582
W-AFR	BurkinaFaso-Mossi	MOS	DQA1#3	~	DQB1#2909	0.094	0.012	0.082	6.028
W-AFR	BurkinaFaso-Mossi	MOS	DRB1#3152	~	DQB1#2899	0.063	0.006	0.057	5.900
W-AFR	BurkinaFaso-Mossi	MOS	DRB1#3147	~	DQB1#2909	0.078	0.010	0.068	5.506
W-AFR	BurkinaFaso-Mossi	MOS	DQA1#6	~	DQB1#2899	0.094	0.015	0.079	5.193
W-AFR	BurkinaFaso-Mossi	MOS	DRB1#3142	~	DQB1#2906	0.094	0.015	0.079	5.186
W-AFR	BurkinaFaso-Mossi	MOS	DQA1#2	~	DQB1#2906	0.094	0.015	0.079	5.186
W-AFR	BurkinaFaso-Mossi	MOS	DRB1#3136	~	DQB1#2901	0.078	0.012	0.066	4.744
W-AFR	BurkinaFaso-Mossi	MOS	DQA1#8	~	DQB1#2901	0.078	0.012	0.066	4.744
W-AFR	BurkinaFaso-Mossi	MOS	DQA1#1	~	DQB1#2902	0.266	0.092	0.173	4.350
W-AFR	BurkinaFaso-Mossi	MOS	DRB1#3151	~	DQB1#2900	0.063	0.010	0.053	4.249
W-AFR	BurkinaFaso-Mossi	MOS	DQA1#10	~	DQB1#2900	0.063	0.010	0.053	4.249
W-AFR	BurkinaFaso-Mossi	MOS	DRB1#3152	~	DQA1#6	0.063	0.010	0.053	4.249
W-AFR	BurkinaFaso-Mossi	MOS	DRB1#3148	~	DQB1#2902	0.078	0.022	0.056	2.997
W-AFR	BurkinaFaso-Mossi	MOS	DRB1#3155	~	DQB1#2902	0.078	0.022	0.056	2.997
W-AFR	BurkinaFaso-Mossi	MOS	DQB1#2902	~	DPB1#67	0.091	0.031	0.061	2.723
W-AFR	BurkinaFaso-Mossi	MOS	DRB1#3148	~	DQA1#1	0.078	0.026	0.052	2.589
W-AFR	BurkinaFaso-Mossi	MOS	DRB1#3155	~	DQA1#1	0.078	0.026	0.052	2.589
W-AFR	BurkinaFaso-Mossi	MOS	DQA1#1	~	DPB1#67	0.094	0.036	0.058	2.397
C-AFR	Chad-BaggaraArabs	BAG	DRB1#3136	~	DQA1#8	0.064	0.009	0.055	5.649
C-AFR	Chad-BaggaraArabs	BAG	DQA1#1	~	DQB1#2902	0.160	0.067	0.092	3.335
C-AFR	Chad-BaggaraArabs	BAG	DQA1#8	~	DQB1#2901	0.138	0.056	0.082	3.282
C-AFR	Chad-BaggaraArabs	BAG	DRB1#3137	~	DQA1#1	0.103	0.052	0.051	2.086
C-AFR	Chad-Dangaleat	DAN	DRB1#3136	~	DQA1#8	0.053	0.003	0.050	9.167
C-AFR	Chad-Dangaleat	DAN	DRB1#3152	~	DQB1#2899	0.053	0.003	0.050	9.167
C-AFR	Chad-Dangaleat	DAN	DRB1#3149	~	DQA1#5	0.064	0.004	0.060	9.058
C-AFR	Chad-Dangaleat	DAN	DQA1#5	~	DQB1#2903	0.064	0.004	0.060	9.058
C-AFR	Chad-Dangaleat	DAN	DRB1#3149	~	DQB1#2903	0.064	0.004	0.060	9.058
C-AFR	Chad-Dangaleat	DAN	DRB1#3142	~	DQA1#2	0.096	0.012	0.083	7.276
C-AFR	Chad-Dangaleat	DAN	DQA1#1	~	DQB1#2902	0.245	0.073	0.171	5.907
C-AFR	Chad-Dangaleat	DAN	DRB1#3145	~	DQA1#6	0.192	0.060	0.132	5.068
C-AFR	Chad-Dangaleat	DAN	DRB1#3155	~	DQB1#2902	0.106	0.027	0.079	4.597
C-AFR	Chad-Dangaleat	DAN	DRB1#3145	~	DQB1#2900	0.190	0.067	0.123	4.432
C-AFR	Chad-Dangaleat	DAN	DRB1#3155	~	DQA1#1	0.106	0.031	0.076	4.142
C-AFR	Chad-Dangaleat	DAN	DRB1#3153	~	DQB1#2902	0.116	0.035	0.080	4.079
C-AFR	Chad-Dangaleat	DAN	DRB1#3153	~	DQA1#1	0.117	0.040	0.077	3.684
C-AFR	Chad-Dangaleat	DAN	DQA1#6	~	DQB1#2900	0.160	0.073	0.086	2.974
C-AFR	Chad-Daza	DAZ	DRB1#3136	~	DQA1#8	0.069	0.006	0.063	6.208
C-AFR	Chad-Daza	DAZ	DRB1#3142	~	DQA1#2	0.259	0.071	0.187	5.145
C-AFR	Chad-Daza	DAZ	DQA1#2	~	DQB1#2907	0.172	0.045	0.128	4.506
C-AFR	Chad-Daza	DAZ	DRB1#3142	~	DQB1#2907	0.172	0.048	0.125	4.254
C-AFR	Chad-Daza	DAZ	DRB1#3136	~	DQB1#2901	0.086	0.018	0.068	3.864
C-AFR	Chad-Daza	DAZ	DQA1#8	~	DQB1#2901	0.069	0.014	0.055	3.463
C-AFR	Chad-Daza	DAZ	DQA1#2	~	DQB1#2906	0.069	0.018	0.051	2.890
C-AFR	Chad-Daza	DAZ	DQA1#1	~	DQB1#2902	0.293	0.144	0.149	2.772
C-AFR	Chad-Daza	DAZ	DRB1#3153	~	DQA1#1	0.172	0.072	0.100	2.747
C-AFR	Chad-Daza	DAZ	DRB1#3153	~	DQB1#2902	0.155	0.072	0.083	2.277
C-AFR	Chad-Maba	MAB	DQA1#6	~	DQB1#2900	0.095	0.026	0.068	3.576
C-AFR	Chad-Maba	MAB	DQA1#10	~	DQB1#2900	0.068	0.018	0.050	3.224
C-AFR	Chad-Maba	MAB	DRB1#3153	~	DPB1#67	0.214	0.118	0.096	2.260

C-AFR	Chad-Maba	MAB	DQA1#1	~	DQB1#2902	0.514	0.344	0.169	2.007
E-AFR	Ethiopia-Amhara-(Keketeya)	AMH	DQA1#9	~	DQB1#2900	0.056	0.003	0.053	8.001
E-AFR	Ethiopia-Amhara-(Keketeya)	AMH	DRB1#3147	~	DQA1#3	0.111	0.012	0.099	7.496
E-AFR	Ethiopia-Amhara-(Keketeya)	AMH	DRB1#3136	~	DQA1#8	0.111	0.015	0.096	6.484
E-AFR	Ethiopia-Amhara-(Keketeya)	AMH	DRB1#3142	~	DQA1#2	0.139	0.023	0.116	6.341
E-AFR	Ethiopia-Amhara-(Keketeya)	AMH	DRB1#3142	~	DQB1#2907	0.083	0.013	0.071	5.274
E-AFR	Ethiopia-Amhara-(Keketeya)	AMH	DRB1#3147	~	DQB1#2909	0.083	0.014	0.069	4.963
E-AFR	Ethiopia-Amhara-(Keketeya)	AMH	DQA1#3	~	DQB1#2909	0.083	0.014	0.069	4.963
E-AFR	Ethiopia-Amhara-(Keketeya)	AMH	DQA1#2	~	DQB1#2906	0.069	0.011	0.059	4.819
E-AFR	Ethiopia-Amhara-(Keketeya)	AMH	DRB1#3148	~	DQB1#2902	0.097	0.022	0.076	4.318
E-AFR	Ethiopia-Amhara-(Keketeya)	AMH	DRB1#3137	~	DQB1#2901	0.167	0.055	0.112	3.915
E-AFR	Ethiopia-Amhara-(Keketeya)	AMH	DQA1#1	~	DQB1#2902	0.194	0.070	0.124	3.836
E-AFR	Ethiopia-Amhara-(Keketeya)	AMH	DRB1#3153	~	DQB1#2902	0.083	0.022	0.062	3.524
E-AFR	Ethiopia-Amhara-(Keketeya)	AMH	DRB1#3137	~	DPB1#71	0.139	0.050	0.089	3.276
E-AFR	Ethiopia-Amhara-(Keketeya)	AMH	DRB1#3137	~	DQA1#1	0.167	0.065	0.101	3.260
E-AFR	Ethiopia-Amhara-(Keketeya)	AMH	DQB1#2906	~	DPB1#71	0.069	0.019	0.050	3.033
E-AFR	Ethiopia-Amhara-(Keketeya)	AMH	DQB1#2902	~	DPB1#67	0.110	0.040	0.069	2.859
E-AFR	Ethiopia-Amhara-(Keketeya)	AMH	DRB1#3148	~	DQA1#1	0.097	0.040	0.057	2.369
E-AFR	Ethiopia-Ethiopians-Oromo	ORO	DRB1#3161	~	DQB1#2904	0.091	0.008	0.083	4.246
E-AFR	Ethiopia-Ethiopians-Oromo	ORO	DRB1#3161	~	DQA1#4	0.091	0.008	0.083	4.246
E-AFR	Ethiopia-Ethiopians-Oromo	ORO	DQA1#4	~	DQB1#2904	0.091	0.008	0.083	4.246
E-AFR	Ethiopia-Ethiopians-Oromo	ORO	DRB1#3136	~	DQA1#8	0.182	0.041	0.140	3.174
E-AFR	Ethiopia-Ethiopians-Oromo	ORO	DQA1#8	~	DQB1#2901	0.182	0.050	0.132	2.715
E-AFR	Ethiopia-Ethiopians-Oromo	ORO	DRB1#3153	~	DQB1#2902	0.091	0.017	0.074	2.691
E-AFR	Ethiopia-Ethiopians-Oromo	ORO	DRB1#3142	~	DQA1#2	0.273	0.101	0.171	2.396
E-AFR	Ethiopia-Ethiopians-Oromo	ORO	DRB1#3153	~	DQA1#1	0.091	0.021	0.070	2.268
E-AFR	Ethiopia-Ethiopians-Oromo	ORO	DRB1#3137	~	DQA1#1	0.091	0.021	0.070	2.268
E-AFR	Ethiopia-Ethiopians-Oromo	ORO	DRB1#3136	~	DQB1#2901	0.182	0.062	0.120	2.186
E-AFR	Ethiopia-Ethiopians-Oromo	ORO	DQA1#1	~	DQB1#2902	0.136	0.041	0.095	2.148
E-AFR	Ethiopia-Ethiopians-Oromo	ORO	DRB1#3142	~	DQB1#2907	0.227	0.087	0.141	2.138
E-AFR	Ethiopia-Ethiopians-Oromo	ORO	DQA1#2	~	DQB1#2907	0.227	0.087	0.141	2.138
N-AFR	Sudan-BejaHadendoa	BEJ	DRB1#3152	~	DQA1#6	0.068	0.006	0.062	7.364
N-AFR	Sudan-BejaHadendoa	BEJ	DQA1#6	~	DQB1#2899	0.068	0.006	0.062	7.364
N-AFR	Sudan-BejaHadendoa	BEJ	DRB1#3142	~	DQA1#2	0.148	0.027	0.121	6.774
N-AFR	Sudan-BejaHadendoa	BEJ	DQA1#2	~	DQB1#2907	0.125	0.021	0.104	6.591
N-AFR	Sudan-BejaHadendoa	BEJ	DRB1#3142	~	DQB1#2907	0.113	0.020	0.093	6.145
N-AFR	Sudan-BejaHadendoa	BEJ	DRB1#3136	~	DQA1#8	0.296	0.094	0.201	5.867
N-AFR	Sudan-BejaHadendoa	BEJ	DRB1#3137	~	DQA1#1	0.125	0.028	0.097	5.298
N-AFR	Sudan-BejaHadendoa	BEJ	DQB1#2907	~	DPB1#72	0.080	0.016	0.064	4.756
N-AFR	Sudan-BejaHadendoa	BEJ	DQA1#1	~	DQB1#2902	0.091	0.021	0.070	4.536
N-AFR	Sudan-BejaHadendoa	BEJ	DQA1#2	~	DPB1#72	0.090	0.021	0.068	4.347
N-AFR	Sudan-BejaHadendoa	BEJ	DRB1#3142	~	DPB1#72	0.080	0.020	0.060	3.926
N-AFR	Sudan-BejaHadendoa	BEJ	DQA1#8	~	DQB1#2901	0.273	0.134	0.138	3.296
N-AFR	Sudan-BejaHadendoa	BEJ	DRB1#3136	~	DQB1#2901	0.273	0.145	0.128	2.922
N-AFR	Sudan-BejaHadendoa	BEJ	DQA1#8	~	DPB1#67	0.149	0.067	0.081	2.847
N-AFR	Sudan-BejaHadendoa	BEJ	DRB1#3136	~	DPB1#67	0.155	0.072	0.083	2.784
N-AFR	Sudan-BejaHadendoa	BEJ	DRB1#3137	~	DQB1#2901	0.125	0.057	0.068	2.606
N-AFR	Sudan-Nubians	NUB	DRB1#3161	~	DQB1#2904	0.067	0.004	0.062	8.834
N-AFR	Sudan-Nubians	NUB	DRB1#3142	~	DQB1#2907	0.133	0.019	0.114	7.723
N-AFR	Sudan-Nubians	NUB	DRB1#3151	~	DQB1#2900	0.066	0.009	0.058	5.858
N-AFR	Sudan-Nubians	NUB	DRB1#3152	~	DQA1#6	0.067	0.013	0.054	4.481
N-AFR	Sudan-Nubians	NUB	DRB1#3142	~	DQA1#2	0.086	0.022	0.063	3.950
N-AFR	Sudan-Nubians	NUB	DRB1#3136	~	DQB1#2901	0.111	0.035	0.075	3.740
N-AFR	Sudan-Nubians	NUB	DQA1#2	~	DQB1#2907	0.077	0.021	0.056	3.661
N-AFR	Sudan-RashaaydaArabs	RAS	DRB1#3137	~	DQA1#1	0.250	0.066	0.184	5.508
N-AFR	Sudan-RashaaydaArabs	RAS	DRB1#3136	~	DQA1#8	0.281	0.088	0.193	4.967
N-AFR	Sudan-RashaaydaArabs	RAS	DQA1#6	~	DQB1#2899	0.156	0.037	0.120	4.907
N-AFR	Sudan-RashaaydaArabs	RAS	DRB1#3140	~	DQB1#2899	0.109	0.022	0.087	4.668
N-AFR	Sudan-RashaaydaArabs	RAS	DRB1#3140	~	DQA1#6	0.141	0.033	0.108	4.665
N-AFR	Sudan-RashaaydaArabs	RAS	DRB1#3137	~	DPB1#69	0.170	0.070	0.100	2.901
N-AFR	Sudan-RashaaydaArabs	RAS	DQA1#1	~	DPB1#69	0.170	0.075	0.095	2.675
N-AFR	Sudan-RashaaydaArabs	RAS	DRB1#3140	~	DPB1#71	0.141	0.066	0.075	2.249
N-AFR	Sudan-RashaaydaArabs	RAS	DQA1#8	~	DQB1#2901	0.297	0.172	0.125	2.201
N-AFR	Sudan-RashaaydaArabs	RAS	DRB1#3137	~	DQB1#2901	0.250	0.145	0.105	2.053
N-AFR	Sudan-SudaneseArabs	SUD	DQA1#2	~	DQB1#2907	0.056	0.004	0.052	7.054

N-AFR	Sudan-SudaneseArabs	SUD	DRB1#3136	~	DQA1#8	0.125	0.017	0.108	6.871
N-AFR	Sudan-SudaneseArabs	SUD	DRB1#3142	~	DQA1#2	0.069	0.007	0.063	6.451
N-AFR	Sudan-SudaneseArabs	SUD	DQA1#4	~	DQB1#2904	0.083	0.009	0.074	6.417
N-AFR	Sudan-SudaneseArabs	SUD	DRB1#3159	~	DQB1#2908	0.069	0.007	0.062	6.340
N-AFR	Sudan-SudaneseArabs	SUD	DRB1#3142	~	DQB1#2907	0.056	0.005	0.050	5.778
N-AFR	Sudan-SudaneseArabs	SUD	DRB1#3147	~	DQB1#2909	0.056	0.005	0.050	5.778
N-AFR	Sudan-SudaneseArabs	SUD	DQA1#3	~	DQB1#2908	0.083	0.014	0.069	4.963
N-AFR	Sudan-SudaneseArabs	SUD	DRB1#3137	~	DQB1#2901	0.111	0.024	0.087	4.667
N-AFR	Sudan-SudaneseArabs	SUD	DQA1#6	~	DQB1#2899	0.069	0.014	0.056	4.054
N-AFR	Sudan-SudaneseArabs	SUD	DRB1#3159	~	DQA1#3	0.069	0.014	0.056	3.970
N-AFR	Sudan-SudaneseArabs	SUD	DQA1#1	~	DQB1#2902	0.153	0.051	0.101	3.704
N-AFR	Sudan-SudaneseArabs	SUD	DRB1#3137	~	DQA1#1	0.111	0.033	0.078	3.589
N-AFR	Sudan-SudaneseArabs	SUD	DQA1#8	~	DPB1#67	0.109	0.033	0.076	3.501
N-AFR	Sudan-SudaneseArabs	SUD	DQA1#8	~	DQB1#2901	0.083	0.024	0.059	3.172
N-AFR	Sudan-SudaneseArabs	SUD	DQA1#3	~	DPB1#71	0.110	0.039	0.070	2.949
N-AFR	Sudan-SudaneseArabs	SUD	DRB1#3136	~	DPB1#67	0.104	0.037	0.068	2.938
N-AFR	Sudan-SudaneseArabs	SUD	DRB1#3136	~	DQB1#2901	0.083	0.027	0.056	2.868
N-AFR	Sudan-SudaneseArabs	SUD	DQA1#1	~	DQB1#2901	0.111	0.051	0.060	2.182
NAFR	Algeria-Constantine	ALC	DRB1#3142	~	DQA1#2	0.081	0.007	0.075	7.879
NAFR	Algeria-Constantine	ALC	DRB1#3140	~	DQB1#2899	0.068	0.005	0.062	7.197
NAFR	Algeria-Constantine	ALC	DRB1#3136	~	DQA1#8	0.081	0.008	0.073	7.184
NAFR	Algeria-Constantine	ALC	DRB1#3160	~	DQB1#2904	0.054	0.004	0.050	7.162
NAFR	Algeria-Constantine	ALC	DQA1#9	~	DQB1#2900	0.068	0.009	0.058	5.239
NAFR	Algeria-Constantine	ALC	DRB1#3140	~	DQA1#6	0.068	0.010	0.058	4.977
NAFR	Algeria-Constantine	ALC	DQA1#4	~	DQB1#2904	0.068	0.010	0.058	4.915
NAFR	Algeria-Constantine	ALC	DRB1#3137	~	DQB1#2901	0.162	0.049	0.114	4.325
NAFR	Algeria-Constantine	ALC	DQA1#8	~	DQB1#2901	0.081	0.021	0.060	3.555
NAFR	Algeria-Constantine	ALC	DRB1#3136	~	DQB1#2901	0.081	0.024	0.057	3.097
NAFR	Algeria-Constantine	ALC	DRB1#3137	~	DPB1#67	0.095	0.031	0.064	3.090
NAFR	Algeria-Constantine	ALC	DRB1#3148	~	DQB1#2902	0.081	0.025	0.056	2.990
NAFR	Algeria-Constantine	ALC	DQA1#1	~	DQB1#2902	0.257	0.130	0.127	2.815
NAFR	Algeria-Constantine	ALC	DQA1#1	~	DPB1#64	0.108	0.045	0.063	2.481
NAFR	Algeria-Constantine	ALC	DRB1#3137	~	DQA1#1	0.162	0.079	0.083	2.432
NAFR	Algeria-Tamanrasset	ALT	DQA1#3	~	DQB1#2909	0.077	0.006	0.071	6.637
NAFR	Algeria-Tamanrasset	ALT	DQA1#6	~	DQB1#2899	0.096	0.013	0.083	5.241
NAFR	Algeria-Tamanrasset	ALT	DQA1#2	~	DQB1#2907	0.077	0.009	0.068	5.184
NAFR	Algeria-Tamanrasset	ALT	DQA1#5	~	DQB1#2903	0.077	0.009	0.068	5.047
NAFR	Algeria-Tamanrasset	ALT	DQB1#2909	~	DPB1#63	0.058	0.007	0.050	4.202
NAFR	Algeria-Tamanrasset	ALT	DQA1#3	~	DPB1#63	0.058	0.007	0.050	4.202
NAFR	Algeria-Tamanrasset	ALT	DQA1#6	~	DPB1#71	0.135	0.039	0.096	3.436
NAFR	Algeria-Tamanrasset	ALT	DQA1#8	~	DQB1#2901	0.096	0.024	0.072	3.314
NAFR	Algeria-Tamanrasset	ALT	DQB1#2902	~	DPB1#64	0.072	0.018	0.054	2.810
NAFR	Algeria-Tamanrasset	ALT	DQA1#1	~	DQB1#2902	0.154	0.059	0.095	2.721
N-AFR	Morocco-Amazigh-(Amizmiz)	AMI	DRB1#3142	~	DQA1#2	0.083	0.007	0.076	6.329
N-AFR	Morocco-Amazigh-(Amizmiz)	AMI	DQA1#6	~	DQB1#2899	0.083	0.009	0.075	5.525
N-AFR	Morocco-Amazigh-(Amizmiz)	AMI	DRB1#3140	~	DQB1#2899	0.104	0.013	0.091	5.499
N-AFR	Morocco-Amazigh-(Amizmiz)	AMI	DRB1#3158	~	DQA1#5	0.063	0.005	0.057	5.487
N-AFR	Morocco-Amazigh-(Amizmiz)	AMI	DRB1#3140	~	DQA1#6	0.083	0.010	0.073	4.923
N-AFR	Morocco-Amazigh-(Amizmiz)	AMI	DQA1#4	~	DQB1#2904	0.083	0.010	0.073	4.923
N-AFR	Morocco-Amazigh-(Amizmiz)	AMI	DQA1#9	~	DQB1#2900	0.146	0.028	0.118	4.836
N-AFR	Morocco-Amazigh-(Amizmiz)	AMI	DRB1#3136	~	DQA1#8	0.167	0.035	0.132	4.821
N-AFR	Morocco-Amazigh-(Amizmiz)	AMI	DQA1#5	~	DQB1#2903	0.063	0.007	0.056	4.791
N-AFR	Morocco-Amazigh-(Amizmiz)	AMI	DRB1#3161	~	DQB1#2904	0.063	0.007	0.056	4.605
N-AFR	Morocco-Amazigh-(Amizmiz)	AMI	DRB1#3158	~	DQB1#2903	0.063	0.009	0.054	3.985
N-AFR	Morocco-Amazigh-(Amizmiz)	AMI	DRB1#3161	~	DQA1#4	0.063	0.010	0.052	3.518
N-AFR	Morocco-Amazigh-(Amizmiz)	AMI	DRB1#3137	~	DQA1#1	0.188	0.061	0.127	3.452
N-AFR	Morocco-Amazigh-(Amizmiz)	AMI	DRB1#3137	~	DPB1#69	0.121	0.035	0.086	3.155
N-AFR	Morocco-Amazigh-(Amizmiz)	AMI	DQA1#1	~	DQB1#2902	0.104	0.030	0.074	2.888
N-AFR	Morocco-Amazigh-(Amizmiz)	AMI	DQA1#8	~	DQB1#2901	0.167	0.063	0.104	2.795
N-AFR	Morocco-Amazigh-(Amizmiz)	AMI	DRB1#3137	~	DQB1#2901	0.188	0.078	0.109	2.604
N-AFR	Morocco-Amazigh-(Amizmiz)	AMI	DRB1#3136	~	DQB1#2901	0.167	0.078	0.089	2.108
N-AFR	Morocco-Amazigh-(Figuig)	FIG	DQA1#2	~	DQB1#2907	0.078	0.008	0.070	9.979
N-AFR	Morocco-Amazigh-(Figuig)	FIG	DRB1#3136	~	DQA1#8	0.162	0.027	0.135	9.975
N-AFR	Morocco-Amazigh-(Figuig)	FIG	DRB1#3163	~	DQB1#2903	0.065	0.006	0.059	9.504
N-AFR	Morocco-Amazigh-(Figuig)	FIG	DRB1#3163	~	DQA1#4	0.065	0.010	0.055	6.727

N-AFR	Morocco-Amazigh-(Figuig)	FIG	DQA1#4	~	DQB1#2904	0.071	0.012	0.059	6.635
N-AFR	Morocco-Amazigh-(Figuig)	FIG	DRB1#3154	~	DQB1#2902	0.110	0.027	0.084	6.305
N-AFR	Morocco-Amazigh-(Figuig)	FIG	DRB1#3137	~	DQB1#2901	0.195	0.071	0.124	5.594
N-AFR	Morocco-Amazigh-(Figuig)	FIG	DQA1#4	~	DQB1#2903	0.065	0.014	0.051	5.253
N-AFR	Morocco-Amazigh-(Figuig)	FIG	DRB1#3137	~	DQA1#1	0.195	0.081	0.114	4.754
N-AFR	Morocco-Amazigh-(Figuig)	FIG	DQA1#8	~	DQB1#2901	0.143	0.057	0.086	4.345
N-AFR	Morocco-Amazigh-(Figuig)	FIG	DQA1#1	~	DQB1#2902	0.208	0.097	0.111	4.211
N-AFR	Morocco-Amazigh-(Figuig)	FIG	DRB1#3136	~	DQB1#2901	0.143	0.059	0.084	4.142
N-AFR	Morocco-Amazigh-(Figuig)	FIG	DRB1#3154	~	DQA1#1	0.110	0.044	0.066	3.795
N-AFR	Morocco-Amazigh-(Figuig)	FIG	DRB1#3142	~	DQB1#2907	0.078	0.007	0.071	10.880
N-AFR	Morocco-Amazigh-(Figuig)	FIG	DRB1#3142	~	DQA1#2	0.084	0.008	0.076	10.384

Supplementary Table S7: Allele diversity statistics per population

Regions	Populations	Allelic richness				Estimated heterozygosity			
		DRB1	DQA1	DQB1	DPB1	DRB1	DQA1	DQB1	DPB1
W-AFR	BED	11.03	7.38	NA	10.64	0.81	0.64	NA	0.83
W-AFR	MAN	12.76	7.63	6.54	10.28	0.88	0.62	0.65	0.81
W-AFR	SRR	11.93	7.81	7.6	8.26	0.86	0.75	0.75	0.78
W-AFR	SEF	12.81	7.96	7.74	10.6	0.9	0.84	0.8	0.86
W-AFR	MAF	11.61	6.98	NA	11.83	0.84	0.77	NA	0.86
W-AFR	GUR	12.27	8.68	6.79	10.82	0.9	0.77	0.71	0.72
W-AFR	GRS	13.99	7.94	6.88	9.82	0.91	0.74	0.67	0.57
W-AFR	MOS	14.78	8.98	8.3	8.88	0.92	0.81	0.83	0.61
C-AFR	BAG	13.53	9.52	9.39	12.55	0.89	0.81	0.78	0.82
C-AFR	DAN	12.48	9.44	8.55	10.57	0.88	0.82	0.81	0.84
C-AFR	DAZ	12.04	7.99	7.33	12.25	0.85	0.78	0.79	0.8
C-AFR	MAB	12.7	8.42	7.33	8.82	0.86	0.6	0.64	0.74
E-AFR	AMH	9.96	8.72	9.57	11.42	0.87	0.82	0.85	0.83
E-AFR	ORO	10	8	8	12.97	0.8	0.8	0.79	0.83
E-AFR	BEJ	12.55	8.84	9.23	9.83	0.83	0.81	0.72	0.85
E-AFR	NUB	16.8	8.96	9.61	12.42	0.92	0.86	0.84	0.85
E-AFR	RAS	11.17	7.24	8.11	9.4	0.79	0.79	0.65	0.7
E-AFR	SUD	17.25	8.66	10.1	15.2	0.93	0.82	0.86	0.86
N-AFR	ALC	16.56	8.69	8.22	15.43	0.91	0.78	0.81	0.87
N-AFR	ALT	14.38	9	7.95	11	0.92	0.85	0.85	0.84
N-AFR	AMI	13.91	8.69	9.68	12.37	0.88	0.84	0.79	0.77
N-AFR	ASN	14.99	8.86	NA	10.3	0.89	0.8	NA	0.8
N-AFR	FIG	14.81	8.5	8.67	9.36	0.9	0.8	0.79	0.77
**Average**		13.2±2.0	8.4±0.7	8.3±1.1	11.1±1.9	0.88±0.04	0.78±0.07	0.77±0.07	0.79±0.08

Regions	Populations	π_n				S_n				D			
		DRB1	DQA1	DQB1	DPB1	DRB1	DQA1	DQB1	DPB1	DRB1	DQA1	DQB1	DPB1
W-AFR	BED	0.06	0.08	NA	0.03	0.22	0.19	NA	0.08	0.95	2.72	NA	3.1
W-AFR	MAN	0.06	0.07	0.07	0.03	0.24	0.2	0.2	0.09	2.02	2.99	3.09	3.5
W-AFR	SRR	0.07	0.09	0.08	0.03	0.21	0.19	0.2	0.09	2.24	3.49	3.07	2.11
W-AFR	SEF	0.08	0.09	0.08	0.03	0.23	0.21	0.2	0.1	2.65	3.71	3.59	2.36
W-AFR	MAF	0.07	0.09	NA	0.03	0.23	0.21	NA	0.1	1.82	3.5	NA	2.62
W-AFR	GUR	0.06	0.08	0.07	0.03	0.22	0.2	0.2	0.09	1.13	2.57	2.51	1.64
W-AFR	GRS	0.07	0.08	0.06	0.02	0.23	0.2	0.2	0.08	1.64	2.4	1.66	0.7
W-AFR	MOS	0.07	0.08	0.08	0.02	0.24	0.2	0.2	0.08	1.66	2.75	2.63	0.9
C-AFR	BAG	0.07	0.09	0.08	0.03	0.22	0.2	0.21	0.09	1.96	3.54	3.14	2.82
C-AFR	DAN	0.07	0.08	0.08	0.03	0.24	0.2	0.2	0.08	1.66	3.09	3.22	2.86
C-AFR	DAZ	0.07	0.09	0.08	0.03	0.24	0.2	0.2	0.1	1.24	3.17	2.93	1.58
C-AFR	MAB	0.08	0.07	0.06	0.03	0.24	0.2	0.2	0.08	1.96	1.99	1.74	1.67
E-AFR	AMH	0.07	0.09	0.08	0.03	0.2	0.2	0.2	0.09	2.74	4.08	3.58	3.09
E-AFR	ORO	0.08	0.09	0.08	0.03	0.22	0.2	0.18	0.1	1.76	3.17	3.14	2.23
E-AFR	BEJ	0.09	0.09	0.08	0.04	0.22	0.2	0.2	0.09	3.16	3.57	3.01	3.2
E-AFR	NUB	0.08	0.09	0.08	0.03	0.24	0.2	0.21	0.09	2.63	3.64	3	2.63
E-AFR	RAS	0.09	0.09	0.07	0.03	0.2	0.21	0.19	0.08	3.75	3.55	2.58	3.05
E-AFR	SUD	0.08	0.09	0.08	0.03	0.23	0.2	0.21	0.1	2.57	3.58	2.67	2.07
N-AFR	ALC	0.08	0.09	0.08	0.03	0.24	0.2	0.21	0.1	2.11	3.36	2.99	1.98
N-AFR	ALT	0.08	0.09	0.08	0.03	0.22	0.2	0.2	0.1	2.02	3.12	3.12	1.94
N-AFR	AMI	0.09	0.09	0.08	0.03	0.23	0.2	0.21	0.1	3.11	3.75	2.71	2.19
N-AFR	ASN	0.08	0.09	NA	0.03	0.22	0.21	NA	0.09	2.79	3.52	NA	2.29
N-AFR	FIG	0.08	0.09	0.08	0.03	0.24	0.2	0.2	0.08	2.84	4.27	3.5	2.88
**Average**		0.07±0.01	0.09±0.01	0.08±0.01	0.03±0.00	0.23±0.01	0.20±0.00	0.20±0.01	0.09±0.01	2.19±0.7	3.28±0.54	2.89±0.51	2.32±0.72

For each population and each locus, this table provides: the geographic region where these populations dwell (W-AFR: West Africa; C-AFR: Central Africa; E-AFR: East-Africa and N-AFR: North Africa); their short name (see Supplementary Table S1); the values of the allelic richness, computed with the rarefaction method (El Mousadik & Petit, 1996) estimating the number of alleles that would be detected if all sample sizes were as small as the smallest sample size used in the study, i.e. of ORO for DRB1 (21), DQA1 (31) and DQB1 (23) and of ALT for DPB1 (32); the estimated heterozygosity, computed with Gene[Rate] (Nunes et al., 2014) ; the average nucleotide diversity index π ; the average number of segregating site S and the value of Tajima's D . « NA » indicates values impossible to compute due to missing data.

Supplementary Table S8

A)

Nucleotide set	Locus (Exon2)	$\pi_n \pm \sigma$	$S_n \pm \sigma$	$D \pm \sigma$
ARS	DRB1	0.162±0.017	0.450±0.025	2.550±0.783
	DQA1	0.099±0.008	0.248±0.007	2.938±0.508
	DQB1	0.157±0.013	0.400±0.009	3.045±0.524
	DPB1	0.043±0.006	0.107±0.000	2.576±0.890
non-ARS	DRB1	0.042±0.005	0.138±0.01	1.563±0.512
	DQA1	0.082±0.007	0.184±0.004	3.087±0.524
	DQB1	0.042±0.004	0.121±0.007	2.273±0.451
	DPB1	0.026±0.003	0.083±0.009	1.721±0.617

A) Average values (± one standard deviation) of the three statistics π_n (average nucleotide diversity per position), Sn (average number of segregating sites per position) and Tajima's D, given for each HLA locus according to the two different nucleotide sets ARS and non-ARS coding and not coding for the antigen recognition site, respectively.

B)

Compared nucleotide sets	Statistic	Locus (Exon 2)	p.Value	adj.pVal
ARS versus non ARS	π_n	DRB1	**6.22e-09**	**1.86e-08**
		DQA1	**5.79e-07**	**8.28e-07**
		DQB1	**6.30e-08**	**9.45e-08**
		DPB1	**5.41e-08**	**9.45e-08**
	S_n	DRB1	**4.34e-09**	**8.33e-09**
		DQA1	**5.50e-10**	**2.06e-09**
		DQB1	**7.03e-09**	**9.88e-09**
		DPB1	**4.75e-10**	**2.06e-09**
	Tajima's D	DRB1	**3.80e-05**	**1.61e-04**
		DQA1	4.23e-01	4.37e-01
		DQB1	**3.93e-05**	**1.61e-04**
		DPB1	**1.06e-04**	**3.52e-04**

B) Results (pValue) of the Kruskal-Wallis rank sum test comparing the distribution of the three statistics πn, Sn and Tajima's D between the ARS and non-ARS nucleotide sets at each HLA locus. The column pValue gives the exact pValue of the Kruskal-Wallis test whereas adj.pValue is the adjusted pValue after correction for multiple testing using the *fdr* method (Benjamini & Hochberg, 1995). Values in bold are significant (adjusted) pValue (α = 0.05) between the two regions for the specific statistic.

C)

Loci pair	Codons	adj. Pvalue		
		π_n	S_n	D
DRB1 / DPB1	ARS	**1.86e-08**	**2.06e-09**	2.67e-01
	non-ARS	**4.01e-08**	**8.33e-09**	1.96e-01
DQB1 / DPB1	ARS	**4.01e-08**	**2.06e-09**	**3.65e-02**
	non-ARS	**4.01e-08**	**1.51e-08**	**1.60e-02**
DQA1 / DPB1	ARS	**1.86e-08**	**1.42e-09**	1.66e-01
	non-ARS	**1.86e-08**	**8.07e-09**	**2.72e-06**
DRB1 / DQB1	ARS	9.61e-01	**8.33e-09**	**1.42e-02**
	non-ARS	7.21e-01	**9.76e-06**	**1.61e-04**
DRB1 / DQA1	ARS	**1.86e-08**	**2.93e-09**	**2.23e-02**
	non-ARS	**1.86e-08**	**7.32e-09**	**5.96e-07**
DQA1 / DQB1	ARS	**4.01e-08**	**2.06e-09**	5.84e-01
	non-ARS	**4.01e-08**	**9.88e-09**	**1.22e-04**

C) Adjusted pVales (adj. pValue) of the Kruskal-Wallis rank sum test after correction for multiple testing using the fdr method (Benjamini & Hochberg, 1995) comparing the distribution of the three statistics πn, Sn and Tajima's D at either ARS or non-ARS nucleotide sets between each pair of HLA loci. Values in bold are significant adjusted pValue (α = 0.05) between loci for the specific statistic and nucleotde set.

Supplementary Table S9.1: dN

Region	Population	DRB1			DQA1			DQB1			DPB1		
		Whole Exon 2	ARS	non-ARS	Whole Exon 2	ARS	non-ARS	Whole Exon 2	ARS	non-ARS	Whole Exon 2	ARS	non-ARS
W-AFR	SRR	7.68e-02	2.04e-01	3.63e-02	7.55e-02	1.02e-01	7.24e-02	9.36e-02	2.28e-01	4.51e-02	3.84e-02	*6.18e-02*	2.93e-02
W-AFR	MAN	6.85e-02	1.89e-01	2.91e-02	6.46e-02	9.07e-02	5.95e-02	7.90e-02	1.91e-01	3.82e-02	3.95e-02	*6.85e-02*	2.83e-02
W-AFR	BED	5.89e-02	1.63e-01	2.55e-02	7.01e-02	9.70e-02	6.66e-02	–	–	–	3.86e-02	*6.27e-02*	2.91e-02
W-AFR	SEF	8.89e-02	2.27e-01	4.45e-02	7.91e-02	1.01e-01	7.57e-02	1.00e-01	2.45e-01	4.75e-02	4.22e-02	*6.58e-02*	3.30e-02
W-AFR	MAF	7.47e-02	1.97e-01	3.51e-02	7.95e-02	1.06e-01	7.42e-02	–	–	–	4.19e-02	*6.31e-02*	3.36e-02
W-AFR	MOS	8.14e-02	2.23e-01	3.61e-02	7.64e-02	1.01e-01	7.37e-02	8.91e-02	2.12e-01	4.39e-02	2.63e-02	*3.55e-02*	2.27e-02
W-AFR	GRS	8.04e-02	2.20e-01	3.58e-02	6.95e-02	9.87e-02	6.32e-02	7.52e-02	1.78e-01	3.68e-02	2.64e-02	*3.80e-02*	2.19e-02
W-AFR	GUR	6.99e-02	1.91e-01	3.16e-02	7.38e-02	1.01e-01	7.00e-02	8.69e-02	2.08e-01	4.26e-02	3.44e-02	*4.83e-02*	2.90e-02
C-AFR	BAG	7.68e-02	2.07e-01	3.47e-02	8.07e-02	1.06e-01	7.56e-02	9.77e-02	2.37e-01	4.69e-02	4.08e-02	*6.15e-02*	3.29e-02
C-AFR	DAN	7.67e-02	2.01e-01	3.68e-02	7.48e-02	9.84e-02	7.19e-02	9.49e-02	2.32e-01	4.55e-02	3.92e-02	*5.70e-02*	3.22e-02
C-AFR	DAZ	7.43e-02	2.09e-01	3.19e-02	7.92e-02	1.06e-01	7.46e-02	9.29e-02	2.23e-01	4.52e-02	3.76e-02	*5.77e-02*	2.99e-02
C-AFR	MAB	8.63e-02	2.32e-01	3.97e-02	6.60e-02	9.06e-02	6.41e-02	7.59e-02	1.82e-01	3.73e-02	3.28e-02	*5.07e-02*	2.59e-02
E-AFR	ORO	8.78e-02	2.43e-01	3.82e-02	8.12e-02	1.04e-01	7.53e-02	9.71e-02	2.35e-01	4.70e-02	4.19e-02	*5.66e-02*	3.63e-02
E-AFR	AMH	7.82e-02	2.23e-01	3.27e-02	8.04e-02	1.06e-01	7.57e-02	9.55e-02	2.29e-01	4.65e-02	4.07e-02	*6.27e-02*	3.23e-02
N-AFR	NUB	9.16e-02	2.54e-01	4.04e-02	7.94e-02	1.02e-01	7.49e-02	9.39e-02	2.26e-01	4.55e-02	3.88e-02	*5.95e-02*	3.08e-02
N-AFR	SUD	9.05e-02	2.51e-01	3.98e-02	8.04e-02	1.04e-01	7.65e-02	8.91e-02	2.13e-01	4.32e-02	4.05e-02	*6.11e-02*	3.26e-02
N-AFR	RAS	9.94e-02	3.03e-01	3.79e-02	8.06e-02	1.02e-01	7.30e-02	8.21e-02	1.96e-01	4.05e-02	3.80e-02	*6.16e-02*	2.88e-02
N-AFR	BEJ	9.86e-02	2.92e-01	3.96e-02	8.09e-02	1.02e-01	7.34e-02	9.26e-02	2.23e-01	4.50e-02	4.31e-02	*6.66e-02*	3.40e-02
N-AFR	AMI	9.66e-02	2.78e-01	4.01e-02	8.04e-02	1.03e-01	7.45e-02	9.96e-02	2.45e-01	4.69e-02	4.01e-02	*6.35e-02*	3.11e-02
N-AFR	ASN	9.13e-02	2.54e-01	4.07e-02	8.19e-02	1.08e-01	7.52e-02	–	–	–	3.92e-02	*5.89e-02*	3.16e-02
N-AFR	FIG	8.67e-02	2.35e-01	3.98e-02	8.12e-02	1.07e-01	7.50e-02	9.16e-02	2.20e-01	4.41e-02	3.70e-02	*5.93e-02*	2.83e-02
N-AFR	ALT	8.51e-02	2.30e-01	3.88e-02	7.98e-02	1.04e-01	7.53e-02	9.82e-02	2.37e-01	4.74e-02	4.01e-02	*5.75e-02*	3.35e-02
N-AFR	ALC	8.46e-02	2.29e-01	3.88e-02	8.00e-02	1.06e-01	7.55e-02	9.63e-02	2.34e-01	4.62e-02	3.93e-02	*6.17e-02*	3.06e-02
W-AFR	Chimpanzee	8.13e-02	2.34e-01	3.26e-02	7.15e-02	8.95e-02	6.56e-02	3.21e-02	4.47e-02	2.76e-02	1.60e-02	*9.54e-03*	1.89e-02

For each population, this table gives the value of dN for the four loci according to the three nucleotides sets: Whole Exon 2, only nucleotides coding for the ARS (ARS) and only nucleotide not coding for the ARS (non-ARS). The final line correspond to those values computed for the Patr genes in the West African chimpanzees population.

Supplementary Table S9.2: dS

Region	Population	DRB1			DQA1			DQB1			DPB1		
		Whole Exon 2	ARS	non-ARS	Whole Exon 2	ARS	non-ARS	Whole Exon 2	ARS	non-ARS	Whole Exon 2	ARS	non-ARS
W-AFR	SRR	5.06e-02	9.13e-02	4.85e-02	9.40e-02	1.38e-01	8.83e-02	5.86e-02	9.27e-02	4.54e-02	9.89e-03	0	1.31e-02
W-AFR	MAN	4.80e-02	4.53e-02	4.96e-02	6.66e-02	1.08e-01	6.08e-02	4.63e-02	7.55e-02	3.50e-02	1.11e-02	0	1.47e-02
W-AFR	BED	4.28e-02	8.88e-02	3.85e-02	8.39e-02	1.13e-01	8.12e-02	–	–	–	9.91e-03	0	1.31e-02
W-AFR	SEF	6.00e-02	6.96e-02	6.91e-02	1.07e-01	1.89e-01	9.68e-02	6.37e-02	1.03e-01	4.88e-02	1.20e-02	0	1.59e-02
W-AFR	MAF	4.61e-02	3.67e-02	5.00e-02	1.06e-01	1.83e-01	9.51e-02	–	–	–	1.28e-02	0	1.70e-02
W-AFR	MOS	5.87e-02	8.50e-02	6.09e-02	8.55e-02	1.31e-01	7.76e-02	6.10e-02	1.05e-01	4.43e-02	1.01e-02	0	1.34e-02
W-AFR	GRS	5.70e-02	8.27e-02	5.99e-02	8.00e-02	1.44e-01	6.92e-02	5.21e-02	8.70e-02	3.86e-02	9.46e-03	0	1.25e-02
W-AFR	GUR	4.49e-02	7.02e-02	4.80e-02	7.96e-02	1.24e-01	7.04e-02	6.08e-02	9.94e-02	4.60e-02	1.19e-02	0	1.58e-02
C-AFR	BAG	5.51e-02	8.22e-02	5.66e-02	9.64e-02	1.68e-01	8.49e-02	6.07e-02	9.99e-02	4.61e-02	1.42e-02	0	1.88e-02
C-AFR	DAN	5.31e-02	7.46e-02	5.77e-02	8.99e-02	1.40e-01	8.08e-02	6.09e-02	1.00e-01	4.58e-02	1.41e-02	0	1.87e-02
C-AFR	DAZ	5.01e-02	7.24e-02	5.41e-02	9.56e-02	1.50e-01	8.22e-02	5.93e-02	1.13e-01	3.90e-02	1.17e-02	0	1.55e-02
C-AFR	MAB	5.59e-02	6.69e-02	6.26e-02	6.41e-02	8.38e-02	6.72e-02	4.43e-02	7.75e-02	3.13e-02	1.08e-02	0	1.43e-02
E-AFR	ORO	6.21e-02	6.97e-02	6.90e-02	1.06e-01	1.90e-01	8.50e-02	5.47e-02	1.01e-01	3.75e-02	1.55e-02	0	2.06e-02
E-AFR	AMH	5.41e-02	8.00e-02	5.70e-02	1.04e-01	1.76e-01	8.99e-02	6.38e-02	1.14e-01	4.51e-02	1.38e-02	0	1.84e-02
N-AFR	NUB	7.07e-02	8.71e-02	7.73e-02	1.04e-01	1.89e-01	8.78e-02	6.25e-02	1.12e-01	4.41e-02	1.39e-02	0	1.85e-02
N-AFR	SUD	7.26e-02	8.90e-02	7.91e-02	1.03e-01	1.77e-01	9.31e-02	6.21e-02	1.13e-01	4.31e-02	1.39e-02	0	1.84e-02
N-AFR	RAS	6.66e-02	5.82e-02	7.00e-02	1.10e-01	2.24e-01	8.79e-02	5.08e-02	8.26e-02	3.90e-02	1.17e-02	0	1.56e-02
N-AFR	BEJ	6.87e-02	5.72e-02	7.33e-02	1.09e-01	2.21e-01	8.40e-02	5.90e-02	9.81e-02	4.46e-02	1.68e-02	0	2.24e-02
N-AFR	AMI	7.21e-02	8.99e-02	7.80e-02	1.09e-01	2.07e-01	9.17e-02	6.92e-02	9.98e-02	5.77e-02	1.20e-02	0	1.60e-02
N-AFR	ASN	6.34e-02	8.25e-02	6.88e-02	1.03e-01	1.88e-01	8.75e-02	–	–	–	1.29e-02	0	1.71e-02
N-AFR	FIG	6.56e-02	8.13e-02	7.10e-02	1.07e-01	1.90e-01	9.36e-02	6.29e-02	1.07e-01	4.65e-02	1.10e-02	0	1.46e-02
N-AFR	ALT	6.26e-02	8.19e-02	6.80e-02	9.76e-02	1.74e-01	8.40e-02	7.09e-02	1.11e-01	5.60e-02	1.34e-02	0	1.79e-02
N-AFR	ALC	6.58e-02	8.77e-02	7.02e-02	9.84e-02	1.60e-01	9.02e-02	6.27e-02	9.91e-02	4.89e-02	1.12e-02	0	1.49e-02
W-AFR	Chimpanzee	5.15e-02	7.96e-02	4.12e-02	1.16e-01	2.19e-01	9.05e-02	4.10e-02	6.33e-02	3.40e-02	8.12e-03	0	1.11e-02

For each population, this table gives the value of dS for the four loci according to the three nucleotides sets: Whole Exon 2, only nucleotides coding for the ARS (ARS) and only nucleotide not coding for the ARS (non-ARS). The final line correspond to those values computed for the Patr genes in the West African chimpanzees population.

Supplementary Table S9.3: dN/dS ratio

Region	Population	DRB1			DQA1			DQB1			DPB1		
		**Whole Exon 2**	**ARS**	**non-ARS**	**Whole Exon 2**	**ARS**	**non-ARS**	**Whole Exon 2**	**ARS**	**non-ARS**	**Whole Exon 2**	**ARS**	**non-ARS**
W-AFR	SRR	1.52	2.23	0.75	0.8	0.74	0.82	1.6	2.46	0.99	**3.88****	*NA*	2.24
W-AFR	MAN	1.43	**4.18****	0.59	0.97	0.84	0.98	1.71	2.52	1.09	**3.55***	*NA*	1.92
W-AFR	BED	1.38	1.84	0.66	0.84	0.86	0.82	–	–	–	**3.89***	*NA*	2.22
W-AFR	SEF	1.48	**3.26***	0.64	0.74	0.54	0.78	1.57	2.38	0.97	**3.52****	*NA*	2.08
W-AFR	MAF	1.62	**5.38****	0.7	0.75	0.58	0.78	–	–	–	**3.27***	*NA*	1.98
W-AFR	MOS	1.39	2.62	0.59	0.89	0.77	0.95	1.46	2.01	0.99	**2.61***	*NA*	1.7
W-AFR	GRS	1.41	2.66	0.6	0.87	0.69	0.91	1.44	2.05	0.95	**2.79***	*NA*	1.75
W-AFR	GUR	1.56	2.71	0.66	0.93	0.81	0.99	1.43	2.09	0.93	**2.89***	*NA*	1.84
C-AFR	BAG	1.39	2.52	0.61	0.84	0.63	0.89	1.61	2.38	1.02	**2.88***	*NA*	1.75
C-AFR	DAN	1.44	2.69	0.64	0.83	0.7	0.89	1.56	2.32	0.99	**2.78***	*NA*	1.72
C-AFR	DAZ	1.48	**2.88***	0.59	0.83	0.7	0.91	1.57	1.96	1.16	**3.22***	*NA*	1.93
C-AFR	MAB	1.54	**3.47***	0.63	1.03	1.08	0.95	1.71	2.35	1.19	**3.03***	*NA*	1.81
E-AFR	ORO	1.41	**3.48***	0.55	0.77	0.55	0.89	1.77	2.33	1.25	**2.7***	*NA*	1.76
E-AFR	AMH	1.45	**2.79***	0.57	0.77	0.6	0.84	1.5	2.02	1.03	**2.94***	*NA*	1.76
E-AFR	NUB	1.29	**2.91***	0.52	0.76	0.54	0.85	1.5	2.02	1.03	**2.79***	*NA*	1.67
E-AFR	SUD	1.25	2.82	0.5	0.78	0.59	0.82	1.43	1.89	1	**2.92***	*NA*	1.77
E-AFR	RAS	1.49	**5.21****	0.54	0.73	0.45	0.83	1.62	2.37	1.04	**3.24***	*NA*	1.85
E-AFR	BEJ	1.44	**5.1****	0.54	0.74	0.46	0.87	1.57	2.28	1.01	**2.56***	*NA*	1.52
N-AFR	AMI	1.34	**3.1***	0.51	0.74	0.5	0.81	1.44	2.46	0.81	**3.34***	*NA*	1.95
N-AFR	ASN	1.44	**3.07***	0.59	0.79	0.58	0.86	–	–	–	**3.05***	*NA*	1.85
N-AFR	FIG	1.32	**2.89***	0.56	0.76	0.57	0.8	1.46	2.06	0.95	**3.36***	*NA*	1.94
N-AFR	ALT	1.36	2.81	0.57	0.82	0.6	0.9	1.39	2.15	0.85	**2.99***	*NA*	1.88
N-AFR	ALC	1.29	2.61	0.55	0.81	0.66	0.84	1.53	2.36	0.95	**3.5***	*NA*	2.06
Nsign. (%)		0 (0%)	**13 (57%)**	0 (0%)	0 (0%)	0 (0%)	0 (0%)	0 (0%)	0 (0%)	0 (0%)	**23 (100%)**		0 (0%)
W-AFR	Chimpanzee	1.58	**2.94***	0.79	0.61	0.41	0.73	0.78	0.71	0.81	1.98	*NA*	1.7

For each population, this table gives the value of the dN/dS ratio for the four loci according to the three nucleotides sets: Whole Exon 2, only nucleotides coding for the ARS (ARS) and only nucleotide not coding for the ARS (non-ARS). « NA » indicates a values impossible to compute (due to dS=0) and « – » a not available value (due to low sample size or untyped locus). Significant values are indicated in bold, with a « * » corresponding to '*': pValue < 0.05, '**': pValue < 0.01 and '***': pValue < 0.001. Nsign. gives the number (and percentage) of populations rejecting the neutrality. The final line correspond to those values computed for the Patr genes in the West African chimpanzees population.

Supplementary Table S10.1: ***Plasmodium falciparum*** **prevalence association with 5 HLA Class II alleles in 20 African populations**

Population	Population	pfpr2000	Region	Distance to Addis Ababa (m)	Alleles frequencies				
					DPB1#66	DRB1#3144	DRB1#3145	DRB1#3149	DRB1#3155
Senegal-Mandenka	MAN	0.62	W-AFR	5'587'076	0.12	0.04	0.11	0.04	0.08
Senegal-Serere	SRR	0.15	W-AFR	6'027'952	0.29	–	0.05	0.06	0.04
Senegal-Fulani	SEF	0.23	W-AFR	5'886'811	0.13	–	0.04	0.05	0.12
BurkinaFaso-Gurmantche	GUR	0.61	W-AFR	4'171'516	0.49	–	0.11	0.13	0.11
BurkinaFaso-Gurunsi	GRS	0.73	W-AFR	4'374'711	0.64	0.06	0.08	0.08	0.14
BurkinaFaso-Mossi	MOS	0.81	W-AFR	4'396'431	0.60	0.04	0.10	0.07	0.07
Chad-BaggaraArabs	BAG	0.18	C-AFR	2'315'664	0.05	–	0.13	0.04	0.05
Chad-Dangaleat	DAN	0.15	C-AFR	2'238'962	0.31	–	0.23	0.06	0.10
Chad-Daza	DAZ	0	C-AFR	2'210'506	0.01	–	0.01	0.01	–
Chad-Maba	MAB	0.13	C-AFR	2'024'661	0.04	–	0.02	–	0.04
Ethiopia-Amhara-(Keketeya)	AMH	0.03	E-AFR	271'572	–	–	–	–	–
Ethiopia-Oromo	ORO	<0.01	E-AFR	268'135	–	–	0.02	–	–
Sudan-BejaHadendoa	BEJ	0.11	E-AFR	775'722	–	–	0.01	–	0.01
Sudan-Nubians	NUB	0	E-AFR	1'574'300	0.03	–	0.03	0.02	0.03
Sudan-RashaidaArabs	RAS	0.10	E-AFR	747'471	0.02	–	0.01	–	–
Sudan-SudaneseArabs	SUD	0.02	E-AFR	1'349'372	–	–	0.08	–	–
Algeria-(Constantine)	ALC	<0.01	N-AFR	4'428'761	0.04	–	0.09	0.01	0.02
Algeria-(Tamanrasset)	ALT	0	N-AFR	3'854'896	0.19	–	0.05	0.09	0.02
Morocco-Amazigh-(Amizmiz)	AMI	0	N-AFR	5'437'490	0.06	–	0.03	–	0.02
Morocco-Amazigh-(Figuig)	FIG	0	N-AFR	4'841'326	0.06	–	0.02	<0.01	0.03

For each population included in the malaria-association analysis, this table provides the short name (see Supplementary Table S1), the value of *P. falciparum* prevalence estimated at the sampling location (pfpr2000), the geographic region, the distance to Addis Ababa (in meters) as well as the estimated frequencies of the 6 HLA Exon 2 sequences identified as strongly correlated with the pfpr2000. The list of all possible nominal HLA alleles associated with each HLA Exon 2 sequence identified in this study is given in SupplementaryTable S3.

Supplementary Table S10.2: Correlation coefficients

	Coefficients			pValues			Adjusted pValues (fdr)		
**Adjusted alleles frequencies**	**Pearson's cor.**	**Kendall's ρ**	**Pearson's τ**	**Pearson's cor.**	**Kendall's ρ**	**Pearson's τ**	**Pearson's cor.**	**Kendall's ρ**	**Pearson's τ**
adj.DPB1#101	-0.24	-0.38	-0.31	0.32	0.09	0.06	0.59	0.26	0.2
adj.DPB1#103									
adj.DPB1#106									
adj.DPB1#107									
adj.DPB1#108	-0.06	0.22	0.19	0.79	0.36	0.24	0.93	0.59	0.45
adj.DPB1#62	-0.52	-0.8	-0.64	**0.02**	**0**	**0**	0.31	**0**	**0.01**
adj.DPB1#63	-0.26	-0.17	-0.17	0.26	0.46	0.29	0.56	0.71	0.5
adj.DPB1#64	0.04	0.21	0.21	0.87	0.37	0.21	0.94	0.59	0.45
adj.DPB1#65	0.01	0.29	0.21	0.98	0.22	0.21		0.47	0.45
adj.DPB1#66	**0.72**	**0.46**	**0.31**	**0**	**0.04**	0.06	**0.01**	0.18	0.2
adj.DPB1#67	-0.23	-0.16	-0.11	0.33	0.51	0.51	0.61	0.71	0.68
adj.DPB1#68	-0.12	-0.08	-0.02	0.61	0.75	0.9	0.86	0.86	0.93
adj.DPB1#69	-0.41	-0.39	-0.29	0.07	0.09	0.08	0.4	0.26	0.23
adj.DPB1#70	**0.34**	0.08	-0.03	0.14	0.72	0.84	0.48	0.86	0.92
adj.DPB1#71	-0.5	-0.73	-0.54	**0.03**	**0**	**0**	0.31	**0.01**	**0.03**
adj.DPB1#72	**0.35**	**0.44**	0.25	0.12	**0.05**	0.13	0.46	0.19	0.35
adj.DPB1#73	0.21	0.02		0.37	0.94		0.67	0.96	
adj.DPB1#74	-0.11	-0.12	-0.08	0.63	0.62	0.65	0.87	0.82	0.8
adj.DPB1#75	-0.29	-0.55	-0.43	0.21	**0.01**	**0.01**	0.56	0.09	0.07
adj.DPB1#76	-0.31	-0.46	-0.37	0.18	**0.04**	**0.03**	0.51	0.18	0.14
adj.DPB1#77	0.18	0.08	0.06	0.45	0.72	0.69	0.74	0.86	0.82
adj.DPB1#79									
adj.DPB1#80	0.01	-0.02	0.03	0.98	0.93	0.84		0.96	0.92
adj.DPB1#81	-0.38	-0.61	-0.53	0.1	**0**	**0**	0.46	**0.04**	**0.03**
adj.DPB1#82	0.04	0.26	0.19	0.88	0.26	0.24	0.94	0.5	0.45
adj.DPB1#83	-0.13	0.12	0.13	0.6	0.62	0.43	0.86	0.82	0.64
adj.DPB1#84	-0.07	0.01	0.03	0.76	0.98	0.84	0.93	0.98	0.92
adj.DPB1#85	**0.32**	-0.14	-0.11	0.17	0.54	0.51	0.5	0.75	0.68
adj.DPB1#86	-0.11	0.09	0.08	0.65	0.71	0.65	0.88	0.86	0.8
adj.DPB1#89	-0.24	-0.39	-0.34	0.3	0.09	**0.04**	0.59	0.26	0.17
adj.DPB1#92	-0.24	-0.38	-0.31	0.32	0.09	0.06	0.59	0.26	0.2
adj.DPB1#94	-0.05	0.26	0.19	0.85	0.26	0.24	0.94	0.5	0.45
adj.DPB1#97									
adj.DQA1#1	**0.33**	**0.33**	0.24	0.16	0.15	0.15	0.5	0.38	0.37
adj.DQA1#10	-0.09	0.22	0.18	0.7	0.35	0.27	0.89	0.59	0.47
adj.DQA1#12		0.3	0.19	0.99	0.2	0.24		0.44	0.45
adj.DQA1#16									
adj.DQA1#2		-0.16	-0.11		0.5	0.51		0.71	0.68
adj.DQA1#3	0.15	**0.35**	0.27	0.53	0.13	0.1	0.81	0.33	0.28
adj.DQA1#4	-0.74	-0.5	-0.37	**0**	**0.03**	**0.03**	**0.01**	0.16	0.14
adj.DQA1#5	**0.49**	**0.47**	**0.36**	**0.03**	**0.04**	**0.03**	0.31	0.18	0.14
adj.DQA1#6	0.06		0.02	0.81	0.98	0.9	0.93	0.98	0.93
adj.DQA1#8	-0.19	-0.28	-0.16	0.43	0.23	0.33	0.72	0.47	0.51
adj.DQA1#9	-0.28	-0.49	-0.36	0.23	**0.03**	**0.03**	0.56	0.16	0.14
adj.DQB1#2899	0.04	-0.11	-0.11	0.88	0.66	0.51	0.94	0.84	0.68
adj.DQB1#2900	-0.19	-0.04		0.42	0.87		0.72	0.93	
adj.DQB1#2901	-0.26	-0.23	-0.16	0.27	0.32	0.33	0.56	0.58	0.51
adj.DQB1#2902	**0.45**	**0.37**	0.28	**0.05**	0.11	0.09	0.32	0.29	0.26
adj.DQB1#2903	0.12	-0.07	-0.04	0.62	0.78	0.79	0.86	0.87	0.92
adj.DQB1#2904	-0.46	-0.35	-0.29	**0.04**	0.13	0.08	0.31	0.33	0.23
adj.DQB1#2905	-0.14	0.16	0.13	0.56	0.51	0.43	0.84	0.71	0.64
adj.DQB1#2906	**0.46**	**0.42**	0.29	**0.04**	0.07	0.08	0.31	0.24	0.23
adj.DQB1#2907	-0.38	-0.49	-0.34	0.1	**0.03**	**0.04**	0.46	0.16	0.17
adj.DQB1#2908	-0.18	-0.07	-0.03	0.46	0.76	0.84	0.74	0.86	0.92
adj.DQB1#2909	0.1	0.07	0.06	0.68	0.76	0.69	0.89	0.86	0.82
adj.DQB1#2910	-0.26	-0.45	-0.36	0.27	**0.04**	**0.03**	0.56	0.18	0.14
adj.DQB1#2915									
adj.DQB1#2918									
adj.DQB1#2919									
adj.DRB1#3135	0.04	0.07	0.02	0.87	0.78	0.9	0.94	0.87	0.93
adj.DRB1#3136	-0.09	-0.07	-0.08	0.71	0.76	0.65	0.89	0.86	0.8
adj.DRB1#3137	-0.41	-0.39	-0.3	0.08	0.09	0.07	0.4	0.26	0.22
adj.DRB1#3138	-0.35	-0.31	-0.21	0.13	0.18	0.21	0.46	0.41	0.45
adj.DRB1#3139	-0.27	-0.25	-0.21	0.25	0.29	0.21	0.56	0.54	0.45
adj.DRB1#3140	-0.47	-0.7	-0.51	**0.04**	**0**	**0**	0.31	**0.02**	**0.03**
adj.DRB1#3142	-0.06	-0.28	-0.16	0.8	0.22	0.33	0.93	0.47	0.51
adj.DRB1#3143	0.26	0.16	0.06	0.26	0.49	0.69	0.56	0.71	0.82
adj.DRB1#3144	**0.74**	**0.31**	0.19	**0**	0.18	0.24	**0.01**	0.41	0.45
adj.DRB1#3145	**0.33**	**0.54**	**0.38**	0.16	**0.01**	**0.02**	0.5	0.1	0.14
adj.DRB1#3147	-0.03	0.22	0.16	0.91	0.35	0.33	0.95	0.59	0.51
adj.DRB1#3148	**0.42**	0.16	0.09	0.06	0.5	0.6	0.4	0.71	0.79
adj.DRB1#3149	**0.51**	**0.45**	**0.34**	**0.02**	**0.04**	**0.04**	0.31	0.18	0.17
adj.DRB1#3150	-0.11	0.04	-0.01	0.66	0.87	0.95	0.88	0.93	0.97
adj.DRB1#3151	-0.05	0.22	0.18	0.82	0.36	0.27	0.94	0.59	0.47
adj.DRB1#3152	0.24	0.03	0.02	0.31	0.88	0.9	0.59	0.93	0.93
adj.DRB1#3153	0.27	**0.43**	0.24	0.24	0.06	0.15	0.56	0.21	0.37
adj.DRB1#3154	-0.36	-0.6	-0.44	0.12	**0.01**	**0.01**	0.46	**0.05**	0.06
adj.DRB1#3155	**0.58**	**0.64**	**0.51**	**0.01**	**0**	**0**	0.17	**0.03**	**0.03**
adj.DRB1#3156	0.28	**0.32**	0.21	0.23	0.18	0.21	0.56	0.41	0.45
adj.DRB1#3157	-0.36	-0.55	-0.44	0.12	**0.01**	**0.01**	0.46	0.09	0.06
adj.DRB1#3158	-0.36	-0.62	-0.49	0.12	**0**	**0**	0.46	**0.04**	**0.04**
adj.DRB1#3159	-0.19	-0.1	-0.03	0.41	0.67	0.84	0.72	0.84	0.92
adj.DRB1#3160	-0.36	-0.66	-0.48	0.12	**0**	**0**	0.46	**0.03**	**0.04**
adj.DRB1#3161	-0.32	-0.4	-0.26	0.17	**0.08**	0.12	0.5	0.26	0.31
adj.DRB1#3163	-0.41	-0.69	-0.52	0.07	**0**	**0**	0.4	**0.02**	**0.03**
adj.DRB1#3164	-0.26	-0.21	-0.11	0.27	0.37	0.51	0.56	0.59	0.68
adj.DRB1#3165	-0.17	0.04	0.08	0.48	0.88	0.65	0.76	0.93	0.8
adj.DRB1#3167	0.06	0.27	0.17	0.8	0.25	0.29	0.93	0.5	0.5
adj.DRB1#3169	-0.15	0.2	0.17	0.52	0.4	0.29	0.81	0.62	0.5
adj.DRB1#3170	-0.1	0.1	0.12	0.69	0.67	0.47	0.89	0.84	0.68
adj.DRB1#3172	-0.06	0.22	0.19	0.79	0.36	0.24	0.93	0.59	0.45
adj.DRB1#3173	-0.13	0.12	0.13	0.6	0.62	0.43	0.86	0.82	0.64
adj.DRB1#3174									
adj.DRB1#3176									
adj.DRB1#3182									
adj.DRB1#3183	-0.26	-0.45	-0.36	0.27	**0.04**	**0.03**	0.56	0.18	0.14
adj.DRB1#3184									
adj.DRB1#3192									
adj.DRB1#3193									
adj.DRB1#3199									
adj.DRB1#3207									

Results of the three correlations tests (Pearson's correlation coefficient, Kendall's ρ and Pearson's τ) computed between the pfpr2000 (Plasmodium falciparum prevalence in year 2000) and the adjusted alleles frequencies taking into account the geographic effect. Columns coefficients gives the value of the correlation coefficients, pValues gives the associated pValues and Adjusted pValues gives the pValues corrected for multiple tests (fdr method). Bold values indicates either coefficients of at least 0.3 and above, or pValues below 0.05.

Supplementary Table S10.3: Detailed results of the linear models fitted on P. falciparum prevalence in year 2000 and DPB1 selective neutrality coefficients

**Model**	Tajima's D ~ pfpr2000			
**Dataset**	*ARS codon*			
Coefficients				
	**Estimate**	**STDER**	**T.value**	**Pr(>|t|)**
Intercept	2.90	0.22	13.12	1.1e-10
pfpr2000	-1.67	0.68	-2.48	0.02
**Res. STDER**	0.79			
**Deg. freedom**	18			
**Mult. R²**	0.25			
**Adj. R²**	0.21			
**F-stat**	6.11			
**pValue**	0.02			

**Model**	Θ_π ~ pfpr2000			
**Dataset**	*ARS codon*			
Coefficients				
	**Estimate**	**STDER**	**T.value**	**Pr(>|t|)**
Intercept	3.41	0.10	32.49	< 2e-16
pfpr2000	-1.09	0.32	-3.38	3.3e-3
**Res. STDER**	0.38			
**Deg. freedom**	18			
**Mult. R²**	0.39			
**Adj. R²**	0.35			
**F-stat**	11.42			
**pValue**	3.3e-3			

**Model**	Θ_S ~ pfpr2000			
**Dataset**	*ARS codon*			
Coefficients				
	**Estimate**	**STDER**	**T.value**	**Pr(>|t|)**
Intercept	1.56	0.03	47.77	<2e-16
pfpr2000	0.02	0.10	0.17	0.86
**Res. STDER**	0.12			
**Deg. freedom**	18			
**Mult. R²**	1.6e-3			
**Adj. R²**	-0.05			
**F-stat**	0.03			
**pValue**	0.86			

**Model**	Tajima's D ~ pfpr2000			
**Dataset**	*non-ARS codon*			
Coefficients				
	**Estimate**	**STDER**	**T.value**	**Pr(>|t|)**
Intercept	1.86	0.17	11.08	1.79e-9
pfpr2000	-0.72	0.52	-1.39	0.18
**Res. STDER**	0.60			
**Deg. freedom**	18			
**Mult. R²**	0.10			
**Adj. R²**	0.05			
**F-stat**	1.92			
**pValue**	0.18			

**Model**	Θ_π ~ pfpr2000			
**Dataset**	*non-ARS codon*			
Coefficients				
	**Estimate**	**STDER**	**T.value**	**Pr(>|t|)**
Intercept	5.12	0.13	41.01	<2e-16
pfpr2000	-1.48	0.38	-3.85	1.2e-3
**Res. STDER**	0.45			
**Deg. freedom**	18			
**Mult. R²**	0.45			
**Adj. R²**	0.42			
**F-stat**	14.81			
**pValue**	1.2e-3			

**Model**	Θ_S ~ pfpr2000			
**Dataset**	*non-ARS codon*			
Coefficients				
	**Estimate**	**STDER**	**T.value**	**Pr(>|t|)**
Intercept	3.12	0.11	27.28	4.29e-16
pfpr2000	-0.54	0.35	-1.53	0.14
**Res. STDER**	0.41			
**Deg. freedom**	18			
**Mult. R²**	0.12			
**Adj. R²**	0.07			
**F-stat**	2.34			
**pValue**	0.14			

Summary statistics of the linear models assessing the relationship between the prevalence of Plasmodium falciparum in year 2000 at sampling sites (pfpr2000) and the values for DPB1 of three molecular diversity indexes related to selection: the Tajima's D, the Θ_π estimator (based on the nucleotide diversity) and the Θ_S estimator (based on the number of segregating sites). The models were fitted on data relative to the codons coding / not coding for the antigen recognition site (ARS codon / non-ARS codon).
